# Fast bootstrap and reliable readout using hidden references for DNA data storage

**DOI:** 10.1002/imt2.70105

**Published:** 2026-01-11

**Authors:** Weigang Chen, Shuang Liu, Quan Guo, Rui Qin, Qi Ge, Tingting Qi, Yingjin Yuan

**Affiliations:** ^1^ School of Microelectronics Tianjin University Tianjin China; ^2^ State Key Laboratory of Synthetic Biology Tianjin University Tianjin China; ^3^ Frontiers Science Center for Synthetic Biology (Ministry of Education), School of Synthetic Biology and Biomanufacturing Tianjin University Tianjin China

**Keywords:** forward–backward algorithm, insertions/deletions, large DNA fragments, multiple‐fold references, watermark sequence, sliding correlation, soft‐decision decoding

## Abstract

Data storage using large DNA fragments enables low‐cost in vivo replication and offers a promising strategy for distributed data applications. However, data readout from massive, unordered sequencing reads requires alignment based on overlapping regions and is complicated by diverse sequencing errors, especially insertion and deletion (indel) errors. Here, we propose a fast and reliable readout framework in a bootstrap manner tailored for data storage using watermarked large DNA fragments. Our scheme transforms the de novo readout into a resequencing‐like workflow through multiple‐fold hidden references, substantially reducing readout complexity. The framework is compatible with sequencing platforms exhibiting diverse error profiles. For technologies with low indel rates, we employ correlation‐based identification and bit‐wise consensus to enable rapid decoding. For indel‐prone platforms, we incorporate progressive read alignment using multiple‐fold hidden references and the forward‐backward algorithm to ensure robust recovery. In vivo experiments on large DNA fragments with different coding efficiencies validated the proposed framework. Error‐free recovery was achieved using Illumina reads (raw error rate of ~0.2%) at a coverage of 0.6–2.5× and using nanopore reads (raw error rate ~5%) at a coverage of 1.6× or 4.3×. These results demonstrate the practicality and scalability of large‐fragment DNA storage for real‐world applications.

## Highlights

- Multiple‐fold hidden references enable a fast and reliable readout scheme in a bootstrap manner for large DNA storage, transforming the *de novo* readout into a resequencing‐like workflow.
- Correlation to the hidden watermark reference identifies low‐error‐rate reads, and bit‐wise consensus generates soft‐decision information, enabling fast data recovery.
- The read‐by‐read forward–backward algorithm corrects indel errors, and alignment to the regenerative reference fills in low‐coverage regions, enabling reliable data recovery.

## Graphical abstract

This study provides a bootstrap readout framework for DNA data storage based on multiple‐fold hidden references. We employ a multi‐stage alignment and error correction strategy, transforming the de novo readout into a resequencing‐like workflow. Correlation to the hidden watermark reference identifies low‐error‐rate reads, and bit‐wise consensus generates soft‐decision information, enabling fast data recovery. In the presence of indels, the read‐by‐read forward–backward algorithm corrects indel errors, and alignment to the regenerative reference fills in low‐coverage regions, enabling reliable data recovery.

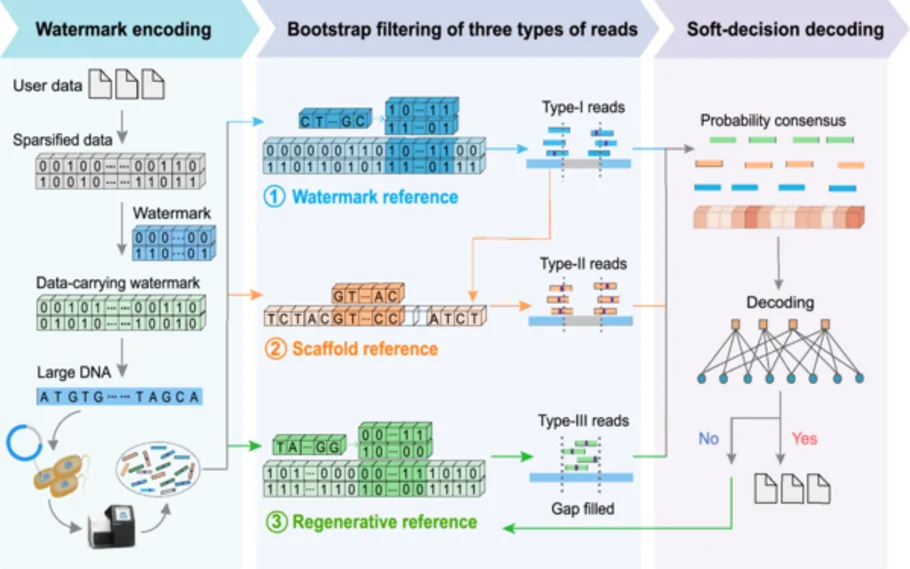

## INTRODUCTION

Synthetic DNA has become a promising data storage medium for data archiving due to its high storage density, long endurance, and low maintenance cost [1, 2, 3, 4]. With the rapid development of DNA synthesis and assembly [5, 6], synthetic DNA fragments of various lengths have been tested for data storage. Compared to data storage using oligo pools [7, 8, 9, 10], large DNA fragments enable low‐cost in vivo replication and are well suited for massive data distribution [11, 12, 13, 14]. For readout, both high‐throughput next‐generation sequencing (NGS) and nanopore sequencing are applicable to large DNA fragments, each offering distinct advantages. NGS provides high throughput and stable performance [15, 16], while nanopore sequencing supports rapid reading of DNA fragments [17, 18, 19], but it suffers from severe insertion and deletion errors, which are typically difficult to correct [20]. Therefore, these two sequencing strategies offer two different routes for the readout of large DNA fragments. However, the assembly complexity and severe sequencing errors are still the main obstacles. First, in current large DNA storage systems, de novo read assembly remains the prevalent strategy for retrieving data from unordered sequencing reads. De novo DNA assembly is often compared to solving a jigsaw puzzle, where sequencing reads are aligned based on overlapping regions [21, 22]. This process demands high sequencing coverage and is computationally intensive [23, 24, 25]. Then, sequencing error profiles vary considerably across different sequencing platforms [26], which exaggerates the difficulty of de novo assembly. For example, Illumina sequencers typically produce a raw error rate of ~0.64% [8]; Ion Torrent sequencers yield ~1.68%, with insertions and deletions (indels) accounting for ~97% of errors [27]; whereas Oxford Nanopore sequencers can reach ~5.2%, with indels accounting for ~62% of errors [28].

Currently, the de Bruijn graph approach is commonly used for short‐read assembly, where reads are usually converted into k‐mers [29, 30, 31], while the overlap‐layout‐consensus paradigm is used for long‐read assembly [32, 33]. In practice, Ping et al. encoded a 54,240‐bp DNA fragment using the Yin‐Yang code and assembled the short reads into contigs with SOAPdenovo [29, 34]. Chen et al. constructed a 254‐kb DNA fragment using low‐density parity‐check (LDPC) codes and superimposed watermarks, and assembled the reads with Velvet at a coverage of 23.5× from NGS reads [30, 35]. They further assembled a 2.5‐Mb ring chromosome by combining Velvet and ABySS using simulation data, achieving error‐free recovery at a coverage of 20× [31, 36]. For nanopore sequencing, the 254‐kb DNA fragment constructed by Chen et al. was assembled using Minimap and Miniasm [32] at a coverage of 16.8×. Sun et al. constructed a 51‐kb DNA fragment using the Mixed Error Processing Coding for Arbitrary Length (MEPCAL) encoding scheme [13] and assembled it using Flye [33] at a minimum coverage of 9.13×. Scaling DNA sequences to larger sizes increases assembly complexity. For example, ABySS2 requires an average of 481 CPU hours to assemble a human genome [37]. In contrast, an end‐to‐end germline workflow can be achieved in just 14 min using the acceleration software Parabricks [38]. The efficiency improvement is largely attributed to resequencing pipelines, where reads are directly aligned to a known reference genome [39, 40, 41]. Therefore, enabling a resequencing‐like pipeline in place of de novo assembly offers a promising way to simplify readout.

In this work, we propose a fast and reliable readout framework for large DNA in a bootstrap manner, based on multiple‐fold hidden references. Inspired by reference‐assisted resequencing pipelines, we suggest the embedded watermark sequence—originally designed to correct indel errors [28, 42]—as a hidden reference to facilitate read alignment. First, the embedded watermark serves as the preliminary reference to rapidly identify low‐error‐rate reads via a correlation‐based method. Bit‐wise consensus was then applied, and soft‐decision decoding was used to enable fast data readout. Second, considering the correlation‐based scheme degrades in the presence of indel errors, we constructed a scaffold reference to identify reads with indel errors, especially when indel errors occurred in the middle of the reads. To address these indel errors, we apply the forward–backward algorithm (FBA) on hidden Markov models (HMMs) to produce indel‐corrected likelihoods for soft‐decision decoding. Third, considering that coverage gaps in the scaffold introduced many erasures, we employed the iterative feedback mechanism to reconstruct a regenerative DNA sequence as the reference. The regenerative reference covered all the DNA sequences and could effectively eliminate erasures.

Recovery results on both Illumina and nanopore sequencing data demonstrated that our method consistently required lower sequencing coverage. For Illumina reads (raw error rate of ~0.2%), the required coverage was reduced from 18–25× to 0.6–2.5× compared with assembly‐based methods under the same dataset. For nanopore reads (raw error rate of ~5%), our method required only 1.6× coverage compared to at least 22× for existing methods under the same nanopore dataset.

## RESULTS

### Multiple‐fold references transform de novo readout to resequencing problem

To retrieve data from large DNA fragments, conventional methods rely on de novo assembly of noisy reads. These approaches require high sequencing coverage and substantial computational resources, and are further complicated by the diversity of sequencing errors (Figure 1A). In our contribution, we constructed multiple‐fold references to support bootstrap and reliable readout, for encoded large DNA fragments with embedded watermark sequences (Figures 1B and S1, Supplementary Note 1, Video S1). The motivation is that this approach transforms de novo assembly into a resequencing problem, reducing the computational complexity and adapting to different types of sequencing errors (Table S1).

**Figure 1:**
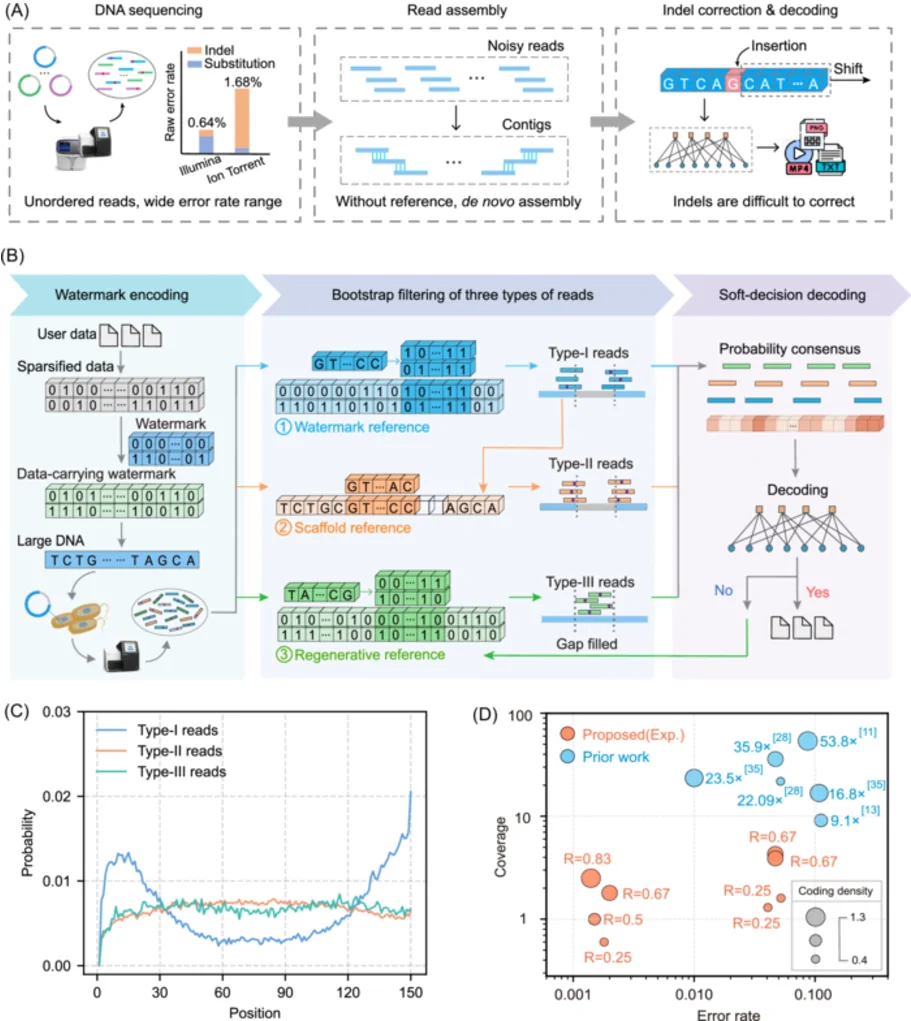
Readout using different filtering schemes according to multiple‐fold hidden references. (A) Existing methods for large DNA fragments after high‐throughput sequencing typically require assembly, which has a high level of complexity. (B) The workflow of multi‐reference‐assisted readout with watermarked large DNA fragments. (C) Distribution of indel error positions across different types of reads in 120 independent experiments. Results are based on nanopore sequencing reads from DNA‐40.5kb‐EM‐ONT‐1 (*R* = 2/3), which exhibit a raw error rate of 4.7%. (D) Comparison with other DNA data storage schemes. Our scheme achieves a lower minimum coverage for data recovery, where *R* denotes the code rate.

On the one hand, multiple‐fold references were used to filter sequencing reads with distinct characteristics. First, the embedded watermark sequences served as the primary reference (watermark reference) to quickly identify high‐correlation reads (Type‐I reads). Type‐I reads either had no indel errors or indel errors occurred near the two ends (Figure 1C). Then, the remaining reads were aligned to a scaffold reference constructed from Type‐I reads, yielding Type‐II reads, which typically carried internal indel errors. Finally, the third reference (regenerative reference) was constructed using a decoding feedback strategy. It was used to recover Type‐III reads—residual reads that failed to align due to gaps in the scaffold reference. In this way, our approach systematically employed all reads in three stages, aligned to three references (Figures S2 and S3).

On the other hand, based on the gradually identified reads, two different soft‐decision information generation methods were proposed. First, we generated bit‐wise probabilistic information for soft‐decision decoding, which enabled rapid data readout. This scheme was developed only for Type‐I reads, which contain fewer errors due to indel errors. Second, the soft‐decision FBA was employed to enable efficient indel correction. It can be used to correct indel errors in all three types of reads.

The proposed readout framework was evaluated through both in vivo experiments and in silico simulations (Figures S4 and S5). Four ~40 kb DNA fragments, encoded with code rates of 1/4, 1/2, 2/3, and 5/6 (Tables S2 and S3), were tested in vivo (Figures S6 and S7). Using 150‐nt Illumina reads with a raw error rate of ~0.2% (Figures S8 and S9), error‐free recovery was achieved at a coverage of 0.6–2.5× (Table S4). Simulation results showed that with a raw error rate of 0.6%, error‐free recovery could be achieved at a coverage of 0.8–3.2×. Using nanopore reads with a raw error rate of ~5%, error‐free recovery was achieved at a coverage of 1.6× or 4.3× with code rates of 1/4 and 2/3, respectively (Figures 1D and S10). Readout performance across different error‐correcting codes evaluated the error‐resilience of our readout scheme, which was reflected by the bit error rate (BER) before error‐free decoding (Table S5). By leveraging consensus among redundant reads, our framework maintained a low BER even under higher raw error rates, enabling reliable performance across both Illumina and Oxford Nanopore sequencing platforms (Table S1).

### Correlation to hidden watermark reference supports rapid read positioning

Assembling unordered reads via overlaps is computationally intensive. For large DNA encoded with the hidden watermark, noisy reads could be directly aligned to the watermark sequence. The proposed method utilized the superimposed watermark sequence as a hidden reference to perform sliding correlation with noisy reads. It enables rapid read positioning according to the correlation peak and avoids the complex de novo assembly.

First, the proposed method could effectively identify the read position under two types of noise (Figure 2A). The watermark sequence in noisy reads suffered from interference from the superposed sparsified data and sequencing errors. On the one hand, the watermark is inherently affected by the superimposed data. To reduce this interference and preserve correlation performance, we apply sparsification to the encoding data. This reduces the correlation peak value from 2*L* to 2*L*(1 − 2*S*), where *L* is the read length, and *S* is the sparsity factor. On the other hand, sequencing errors such as substitutions and indel errors distort the embedded watermark. Correlation is most effective for reads with few substitutions or short bursts of indel errors near the ends (Figure S11). Analysis of the ideal and spurious correlation peaks shows a large difference between them, enabling clear discrimination (Figure 2B).

**Figure 2:**
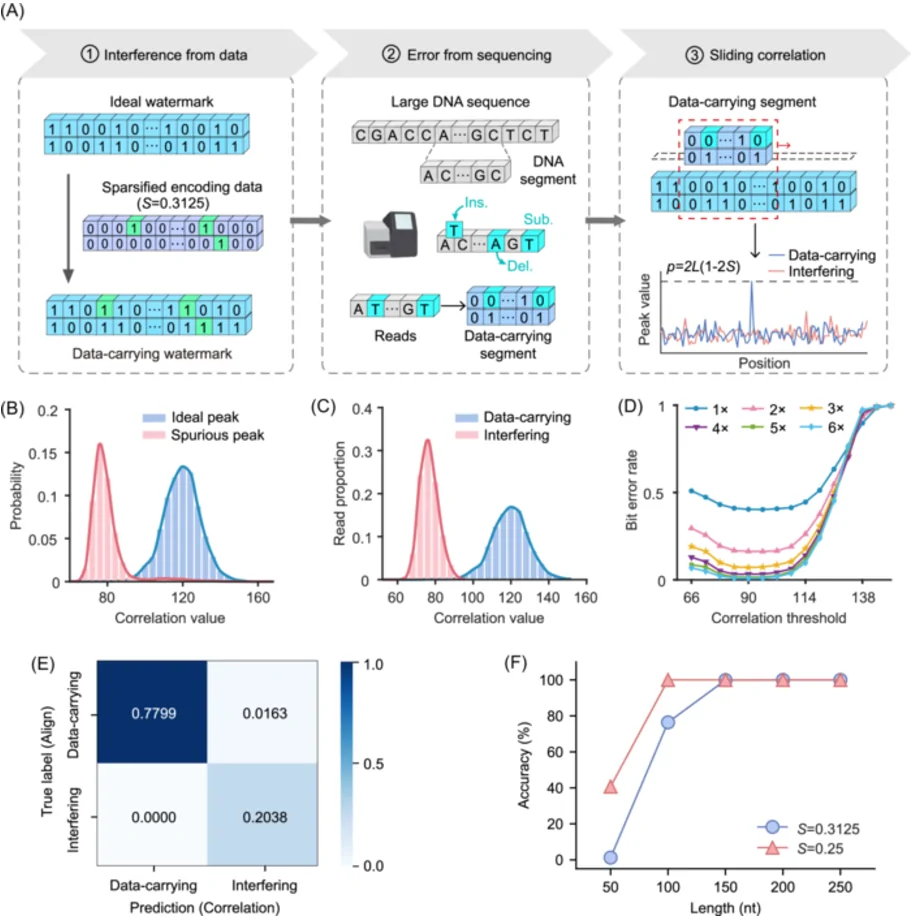
Read positioning using correlation between noisy reads and watermark reference. (A) Workflow of the sliding correlation peak detection for alignment. (B) Distribution of ideal and spurious correlation peaks of data‐carrying segments. The ideal peaks represent the correlation values at the ground‐truth positions (determined by sequence alignment), whereas the spurious peaks denote the maximum correlation values observed at incorrect positions. (C) Comparison of correlation peak values between data‐carrying reads and interfering reads. (D) Bit error rate (BER) of consensus sequences as a function of correlation threshold. (E) The accuracy of the correlation‐based method to distinguish between data‐carrying and interfering reads. (B–E) show results derived from DNA‐40.5kb‐EM‐SE150 (*R* = 2/3). (F) Correlation accuracy for sequencing reads of varying lengths. Results are based on simulated reads from DNA‐2.99Mb‐Sim (*R* = 2/3, *S* = 0.3125) and DNA‐3.59Mb‐Sim (*R* =  2/3, *S* = 0.25), with a raw error rate of 0.3%, where *S* denotes the sparsity ratio.

Then, the proposed method effectively filtered out interfering reads, which typically originate from plasmid backbones, host genome, or severe sequencing errors. These reads show much lower correlation peaks than valid data reads and can be excluded by applying a threshold (Figures 2C and S12). The optimal threshold is determined by minimizing the consensus BER before decoding (Figures 2D and S13, Table S3). The correlation threshold was evaluated using Receiver Operating Characteristic (ROC) and Precision‐Recall (PR) analyses (Supplementary Note 2). Prediction labels were generated from correlation outputs under varying thresholds (66–150, *step* = 1), and ground‐truth labels were determined from sequence alignments (Figure S14). Based on the design consideration that interfering reads degrade BER more severely than the loss of a few data‐carrying reads, the optimal ROC point corresponds to maximal True Positive Rate at False Positive Rate (FPR) = 0, and the optimal PR region to maximal Recall at Precision = 1. The highlighted markers denote the preselected thresholds, which align well with these optimal regions (Figures S15 and S16, Table S6). We assessed the accuracy of threshold‐based classification using alignment‐derived ground‐truth labels. Classification accuracy reached ~99% with plasmid backbone interference (~20% of reads) and remained comparable under host genome interference (~99.5% of reads) (Figures 2E, S17, and S18). This accurate classification contributed to a high read usage ratio (Figure S19), which is defined as the proportion of correctly identified data‐carrying reads. In this context, potential in vivo interferents—including chimeric reads, repeat‐induced spurious correlations, and rearrangements—were not misclassified as valid reads [43]. Further analysis showed that most filtered reads contained no indel errors, with only ~0.64% affected (Figure S20 and Table S7), confirming that correlation enables the extraction of high‐fidelity reads directly from raw sequencing reads.

Finally, we evaluated the impact of read length, coding sparsity, and sequencing error rate on correlation accuracy (Figure 2F, Supplementary Note 3). A large DNA fragment encoded with an LDPC code of the rate 2/3 was evaluated using simulations (Figure S21). For a sparsity ratio of 0.3125, the identification accuracy improved from 75% to 100% when the read length increased from 100 to 150 nt. It indicated that longer reads had enhanced correlation accuracy. We further evaluated the effect of coding sparsity. Lower sparsity ratios spread the original information across longer watermarks, reducing interference and enabling more precise read localization. The 4‐bit‐to‐5‐bit scheme was ultimately chosen for an optimal tradeoff between information density and robustness. We examined performance under error rates from 0.4% to 1.2%. Although the overall read utilization decreased with higher error rates, the successfully identified reads consistently exhibited high positional accuracy (Figure S22).

### Bit‐wise consensus and probability generation for soft‐decision recovery

After Type‐I read positioning, consensus bit sequences were obtained by performing bit‐wise majority voting. Then, error correction was performed. However, modern error correction codes primarily rely on soft‐decision decoding with probabilities as input. Hard‐decision decoding cannot achieve optimal error correction capability due to the loss of probabilistic information for each bit. To address this issue, we developed a pragmatic bit‐wise consensus and probability generation method. It guaranteed the soft‐decision inputs to the LDPC decoder and enhanced the accuracy of data recovery.

First, we developed a rapid recovery method based on bit‐wise consensus and probability generation (Figure 3A). Bit‐wise consensus was obtained through low‐complexity majority voting. Watermark removal and desparsification were then applied to reconstruct the probability consensus. This was followed by the computation of Log‐Likelihood Ratios (LLRs) to generate soft information for LDPC decoding. Across four code rates, the original data were successfully recovered at a coverage of 0.6–2.5× (Figure 3B and Table S8). This was further supported by the distribution of minimum coverage, which confirmed that reliable recovery can be achieved even at low coverage levels (Figure 3C). In addition, independent experiments on multiple datasets yielded consistent results, indicating stable and reliable performance (Figure S23 and Tables S9–S11).

**Figure 3:**
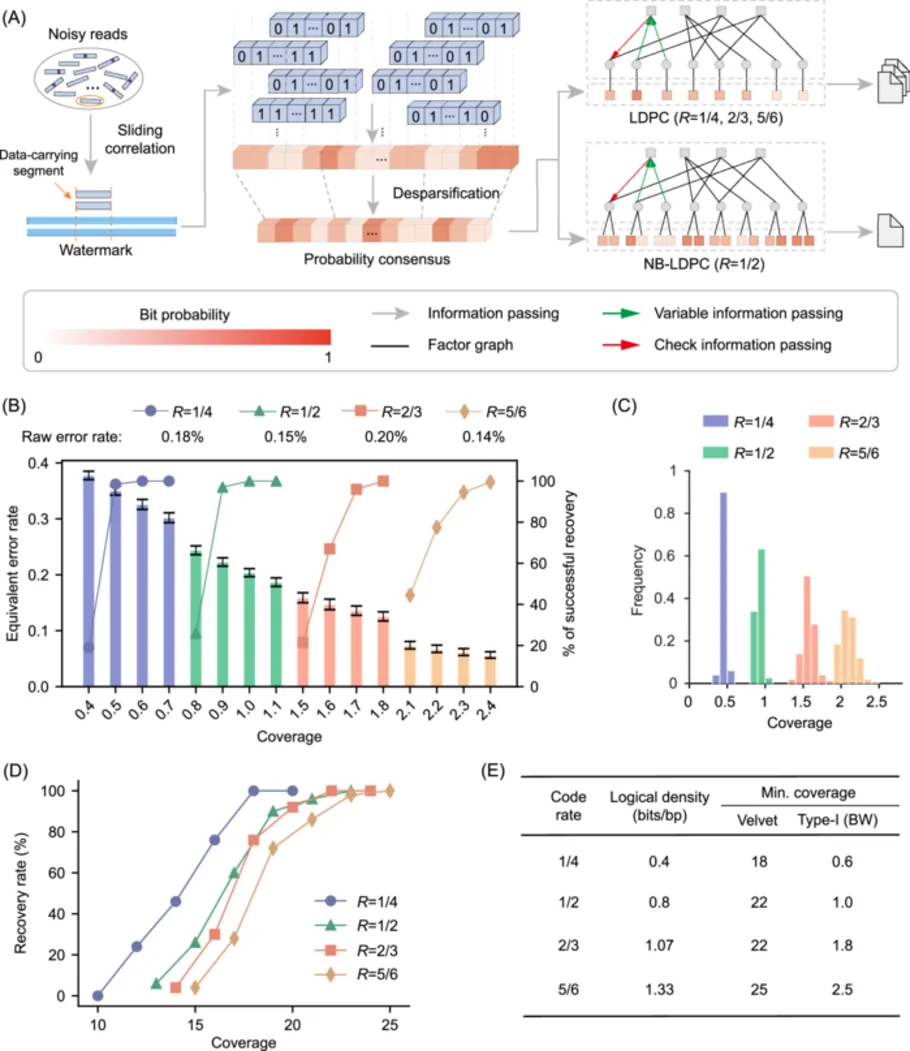
Soft‐decision data recovery using bit‐wise consensus. (A) Workflow of the proposed data recovery method. (B) Recovery performance without genome interference. Each point represents 200 trials with 150‐nt reads. *Equivalent error rate calculated as *E*_erasure_ /2 + *E*_error_. (C) Minimum sequencing coverage required for error‐free recovery across 150 trials at different low‐density parity‐check (LDPC) code rates. (D) Recovery performance of the Velvet‐based method. (E) Comparison of the proposed recovery scheme with an assembly‐based approach (Velvet). The table summarizes the corresponding logical density and the minimum coverage required for error‐free recovery at different code rates. (B–E) show results derived from DNA‐40.5kb‐DR‐SE150 (*R* = 1/4), DNA‐40.32kb‐ER‐SE150 (*R* = 1/2), DNA‐40.5kb‐EM‐SE150 (*R* = 2/3), and DNA‐40.5kb‐MC‐SE150 (*R* = 5/6).

Then, we conducted pooled‐fragment experiments to empirically verify the robustness of the correlation‐based readout scheme against cross‐fragment interference (Figure S24). The correlation‐based classification accurately distinguished reads originating from the two plasmids—each embedded with distinct watermark sequences—with an accuracy of 99.78% (Figure S25 and Table S12). Analysis of the misclassified reads revealed that they typically contained severe base errors.

Finally, we compared our readout scheme with the assembly‐based method (Figure 3D,E, Supplementary Note 4). Velvet is a highly efficient de novo genome assembler. Compared with other assemblers (e.g., SPAdes [44] and MEGAHIT [45]), it achieved shorter runtime and a lower indel error rate in the assembled sequences, making it well suited for DNA data storage readout applications [35]. Therefore, we selected Velvet as the baseline for comparison [46]. Consensus sequences generated by assembly exhibited more gaps, leading to severe erasures before decoding. This is due to the high error sensitivity of de Bruijn graph assemblers, which filter uncertain read segments aggressively and often produce fractional assemblies [47]. Typically, the assembly software Velvet required higher sequencing coverage to compensate for missing or trimmed reads. In contrast, our method consistently achieved error‐free data recovery at lower sequencing coverage using the same datasets and computational environment (Tables S4 and S13). This demonstrates the practical advantages of our scheme in reducing sequencing cost.

### Read‐by‐read forward–backward algorithm corrects insertions and deletions

While bit‐wise consensus enabled rapid data readout, it was incapable of correcting indel errors. Indel errors typically cause burst substitution errors, especially when indel errors occur in the middle of the read. To address this problem, we proposed an enhanced strategy to identify and correct reads with indel errors (Figure 4A). This strategy integrates a read‐by‐read FBA with scaffold‐guided alignment to effectively rescue indel‐containing reads.

**Figure 4:**
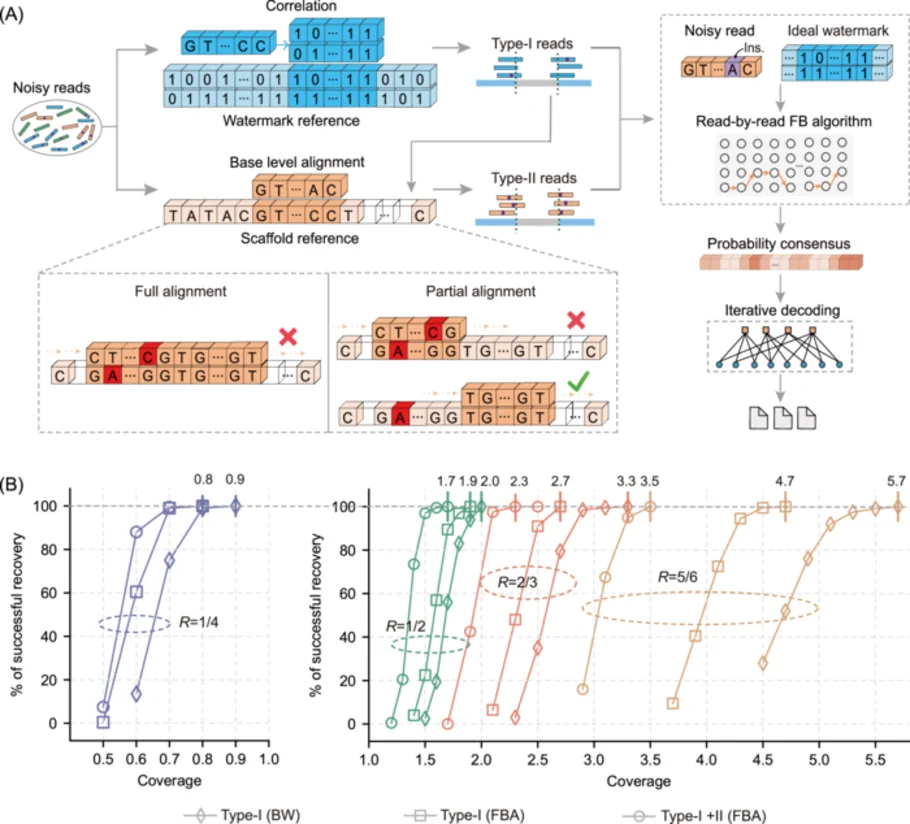
The forward–backward algorithm (FBA) corrects indel errors. (A) Workflow of indel correction. Partial‐length alignment improves the proportion of reads identified. (B) Comparison of recovery results using different types of reads and soft‐decision information generation schemes (BW: Bit‐wise probability generation; FBA: Probability generation with FBA). Recovery tests were performed on datasets including DNA‐40.5 kb‐DR‐Sim‐1 (*R* =  1/4), DNA‐40.32kb‐ER‐Sim‐1 (*R* = 1/2), DNA‐40.5 kb‐EM‐Sim‐1 (*R* = 2/3), and DNA‐40.5 kb‐MC‐Sim‐1 (*R* = 5/6), all of which were simulated with a raw error rate of 0.6%.

First, we developed a read‐by‐read FBA to correct indel errors, reducing error propagation (Supplementary Note 5). Specifically, a soft‐decision FBA on HMMs was employed to learn the watermark degradation pattern and to infer the true encoded symbols (Figure S26). This approach enabled the correction of indel errors and generated high‐quality soft information. Then, to recover additional usable reads, we constructed scaffold references using Type‐I reads through base‐wise consensus. Since the scaffold reference contained both erasure and substitution errors, direct alignment of noisy reads was challenging (Figures S27 and S28). To address this, a partial‐length alignment strategy was employed, where read segments were independently aligned to the scaffold reference (Figure S29, Supplementary Note 6). Once a segment was successfully aligned, the inferred position of the read was determined. Reads successfully aligned in this manner were classified as Type‐II reads, which typically contained indel errors in their middle regions.

We evaluated the impact of FBA and the incorporation of Type‐II reads on data recovery performance. Validating the robustness of readout methods using simulated datasets has proven to be an effective approach [28, 48, 49]. The evaluation was based on simulated reads designed to mimic the high indel error rates of Ion Torrent sequencers, which pose challenges to conventional recovery schemes [27, 50]. We first evaluated recovery performance with FBA correction applied to Type‐I reads. Compared to direct bit‐wise probability generation (BW), probability generation with FBA reduced the number of substitution errors before decoding (Figure S30). We then examined the additional benefits of incorporating Type‐II reads recovered through scaffold alignment. At a raw error rate of approximately 0.6%, partial alignment identified 24.79% more reads (Figure S31A,B), which slightly increased to 25.3% at an error rate of 1.2% (Figure S32A,B). Notably, higher sequencing coverage improved scaffold quality, resulting in more reads being successfully aligned to the scaffold (Figures S31C and S32C). Building on the gains from FBA, the incorporation of Type‐II reads further reduced substitution and erasure rates before decoding. As a result, error‐free recovery was achieved at a coverage of 0.8–3.5× at an error rate of 0.6% (Figures 4B and S33), and 0.8–4.2× at an error rate of 1.2% (Figure S34).

### Iterative alignment to regenerative reference fills in low‐coverage gap

Scaffold references constructed from Type‐I reads typically contain gaps, which correspond to low‐coverage regions. Type‐I and Type‐II reads generally span the same overlaps. These gaps lead to erasure errors under low sequencing coverage. To address this problem, we fed the decoding results back to generate a regenerative reference, which helped identify reads to fill the codeword gaps (Figure 5A, Supplementary Note 7). Iterative decoding of LDPC codes on a factor graph allowed probability information to pass between nodes, thereby eliminating erasures within the codeword. The decoded codeword was then reconstructed into a long DNA fragment, retaining only substitution errors after sparsification and watermark superposition. Gaps in the scaffold reference were then filled, enabling the identification of reads from low‐coverage regions (Figure S35). Bit‐level alignment was employed to prevent error accumulation caused by misalignment of bit‐error positions (Figures S36 and S37).

**Figure 5:**
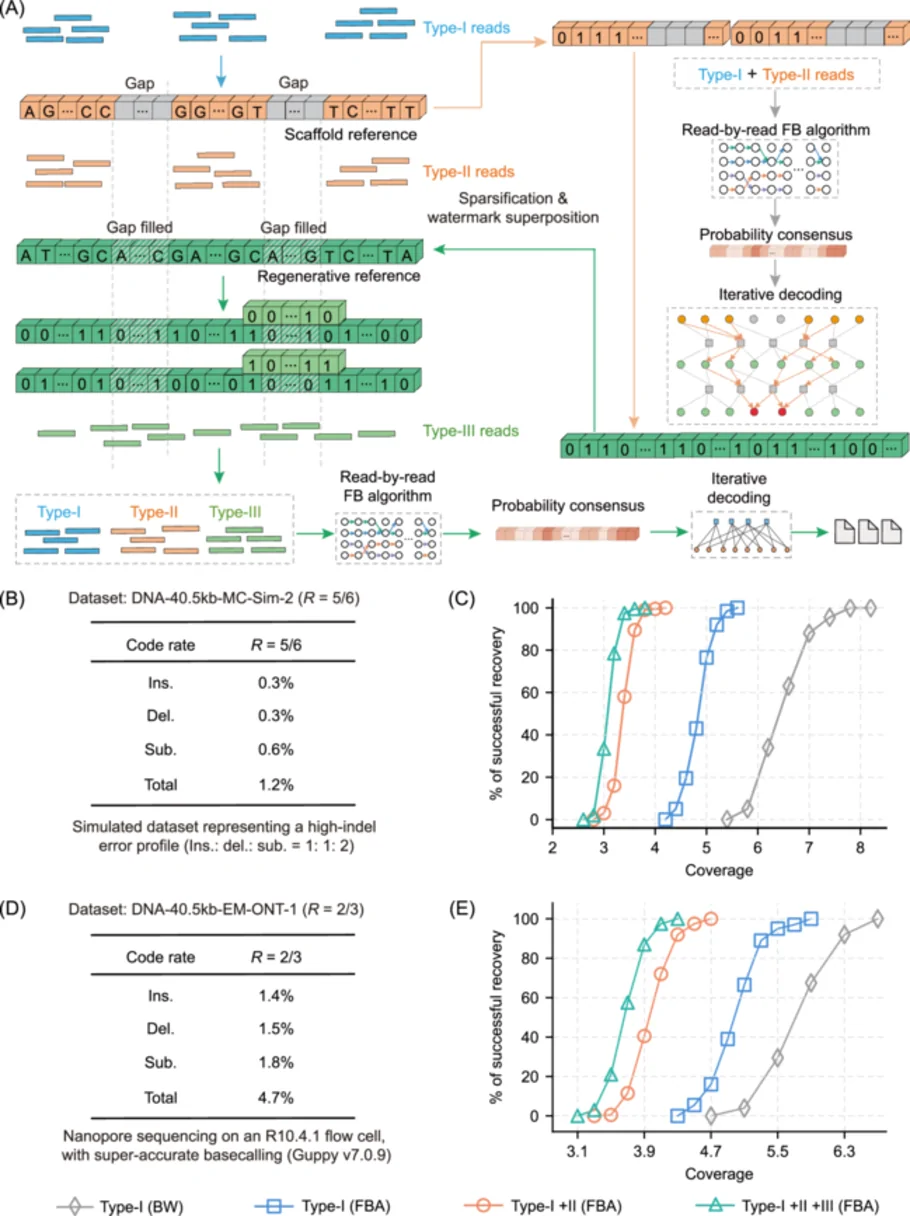
Gap‐filling scheme with regenerative reference. (A) Workflow of the gap‐filling scheme. Gaps in the scaffold reference are filled through read alignment to regenerative reference. (B) Error profile of the simulated dataset (DNA‐40.5kb‐MC‐Sim‐2, *R* = 5/6). (C) Recovery performance under different sequencing coverages for the dataset in (B). (D) Error profile of the nanopore sequencing dataset (DNA‐40.5kb‐EM‐ONT‐1, *R* = 2/3). (E) Recovery performance under different sequencing coverages for the dataset in (D). In (C) and (E), each data point represents 200 independent trials.

First, we performed simulations under varying error rates to validate the effectiveness of the complete readout framework operating in a bootstrap manner. Simulation results showed that progressively recovering Type‐I, Type‐II, and Type‐III reads substantially improved read utilization. Distinguished by their error profiles and positional indel distributions, Type‐I reads have relatively low error rates, whereas Type‐II and Type‐III reads exhibit higher total error rates with indel errors enriched in the middle regions (Figures S38 and S39). Type‐II reads aligned to scaffold‐supported regions refined consensus quality in covered areas, while Type‐III reads from scaffold gaps further reduced residual erasures (Figure S40). With a raw error rate of 0.6%, error‐free recovery was achieved at 0.8–3.2× coverage (Figures S41–S44), increasing only slightly to 0.8–3.7× at an error rate of 1.2%", demonstrating the robustness of the readout in a bootstrap manner (Figure 5B,C and Figures S45–S48). We observed that higher sequencing coverage led to improved scaffold and regenerative references quality and a higher read utilization. Consequently, readout in a bootstrap manner contributed more substantially in high‐efficiency coding scenarios (*R* = 0.93), where greater coverage is typically required (Figures S49 and S50).

Then, we further validated our method on real sequencing data. For nanopore reads—which are variable in length and exhibit higher error rates—direct correlation with the watermark is impractical. Therefore, we divided the long reads into short uniform‐length fragments (150 nt in practice), allowing ~20% of reads to be successfully identified—primarily those with indel errors near the read ends (Figures S51–S53). These identified reads subsequently initiated the decoding process in a bootstrap manner. At a raw error rate of ~5%, partial‐length alignment identified 17.4% more reads than full‐length alignment, but also introduced an additional misidentification rate of 1.2%. Error‐free recovery was achieved at a coverage of 1.6× or 4.3× with code rates of 1/4 and 2/3 (Figure 5D,E and Figures S52–S55). A parallel nanopore sequencing experiment on the same plasmids yielded similar results, achieving error‐free recovery at 1.3× and 3.9× coverage (Figures S56–S59). Both nanopore experiments showed a sizable unused‐read fraction. For DNA‐40.5kb‐EM‐ONT‐1 (*R* = 2/3), ~30% of sequenced reads were unused (Figure S55), comprising in vivo interferents (e.g., vector backbone and *Escherichia coli*) and high‐error data‐carrying reads (Figure S60). Interferents are inherent to in vivo storage, whereas the elevated error rate chiefly limits read‐utilization efficiency. By contrast, evaluation on Illumina sequencing data showed that over 90% of reads could be accurately identified through correlation alone, substantially reducing the marginal benefit of subsequent refinement using scaffold and regenerative references (Figures S61–S65). These findings confirm that the proposed framework remains lightweight for low‐indel data while offering substantial gains in high‐error contexts.

Finally, we evaluated the computational efficiency of our readout scheme. Observed wall‐time and peak random access memory (RAM) across various datasets can serve as empirical indicators of time and space complexity (Tables S14–S20). We noted that accelerated sequence alignment algorithms developed in bioinformatics could potentially speed up the alignment stage [51, 52]. Accordingly, Minimap2 was integrated to enhance alignment efficiency through optimized indexing and seeding algorithms [51]. The readout pipeline using Minimap2 achieved tens‐fold acceleration in the identification of Type‐II and Type‐III reads, while maintaining comparable overall performance to the Edlib implementation [53] (Figures S66 and S67, Table S21).

## DISCUSSION

We propose a fast and reliable readout framework operating in a bootstrap manner for DNA data storage based on multiple‐fold hidden references. This approach integrates a multi‐stage alignment and soft‐decision error correction strategy, transforming the de novo readout into a resequencing‐like workflow.

First, for the watermark encoded DNA, the hidden watermark sequences were used as primary references to rapidly identify Type‐I reads. A bit‐wise method was used to generate consensus soft information for LDPC decoding, enabling rapid data recovery. Then, in the presence of indel errors, we constructed an indel‐resilient scaffold reference to identify Type‐II reads. Since gaps in the scaffold can leave some reads unaligned, we refine the reference using decoding results to identify reads in low‐coverage regions. Furthermore, we proposed a method to generate consensus soft information based on the FBA. It effectively avoided the burst errors caused by indel errors and reduced the required sequencing coverage. Rapid data recovery was achieved on Illumina sequencing data across four code rates at 0.6–2.5× coverage, lower than the ~20× typically required by assembly‐based methods (Figure 3D). Under high‐indel conditions with nanopore reads (raw error rate of ~5%), the framework achieved error‐free recovery at 1.6× and 4.3× with code rates of 1/4 and 2/3, respectively.

Large DNA fragment storage can leverage efficient DNA replication and large‐scale manufacturing [35]. Combined with the proposed efficient readout strategy, it represents a highly promising mode for DNA data storage. The proposed scheme can adapt to various sequencing technologies. For equipment with a low indel error rate, our approach chooses the correlation and bit‐wise consensus to enable rapid data recovery. For equipment with more indel errors (e.g., Nanopore and Ion Torrent sequencers), our approach searches completely all the reads using three references and the FBA to achieve reliable data recovery [54].

Nowadays, synthesis cost remains the main obstacle for data storage using large DNA. In data distribution scenarios, many readouts can share the high cost of synthesizing large DNA fragments. We expect that our contribution will promote the feasibility of data storage using large DNA, especially for data distribution scenarios. Also, with the development of the synthesis technology and operation automation, the cost is always decreasing [55, 56]. Therefore, the proposed schemes should become more popular with the new development of DNA synthesis.

## METHODS

### Workflow of data storage using large DNA

This study adopts a data storage framework based on watermark superposition coding, supporting fast and bootstrap‐based readout workflows for large DNA fragments. A total of three image files and one text file were encoded and synthesized into four ~40 kb DNA sequences using different LDPC code rates (Figure S4 and Table S2). DNA synthesis and assembly were outsourced to Tsingke Biological Technology.

The readout process is divided into two workflows tailored for different error scenarios. The fast readout workflow targets low‐indel platforms (e.g., Illumina) and leverages sliding correlation and bit‐wise consensus for rapid data recovery. In contrast, data readout in a bootstrap manner handles indel‐prone conditions (e.g., Nanopore and Ion Torrent) by progressively identifying reads through multiple‐fold hidden references (watermark reference, scaffold reference, and regenerative reference), combined with FBA to ensure reliable recovery (Figures S1 and S2). Supplementary Note 8 provides detailed steps for reproducibility using a pre‐built Docker image.

### Large DNA design and synthesis

In vivo experiments were performed on four large DNA fragments, including DNA‐40.5kb‐DR (*R* = 1/4), DNA‐40.32kb‐ER (*R* = 1/2), DNA‐40.5kb‐EM (*R* = 2/3), and DNA‐40.5kb‐MC (*R* = 5/6), where DR, EM, ER, and MC are abbreviations of the source data *Dream.txt*, *Emblem.jpg*, *Earth rise.jpg*, and *Milk coronet.jpg*, respectively. DNA‐40.5kb‐DR (*R* = 1/4) and DNA‐40.5kb‐EM (*R* = 2/3) were synthesized by Beijing Tsingke Biotech Co., Ltd. and cloned into the pCC1413 vector, yielding 51,339 and 51,331 bp circular plasmids (Figure S7). The plasmids were extracted using alkaline lysis and alcohol precipitation, followed by electrotransformation into *Escherichia coli* EPI300. NGS was performed to verify the accuracy of the data chunks. DNA‐40.32kb‐ER (*R* = 1/2) and DNA‐40.5kb‐MC (*R* = 5/6) were synthesized in our previous work [35].

### Shotgun sequencing of individual large DNA fragments

For both DNA‐40.5kb‐DR (*R* = 1/4) and DNA‐40.5kb‐EM (*R* = 2/3), 0.2 μg of input plasmid DNA was used for library preparation. The DNA was randomly fragmented by sonication to the desired size range, followed by end polishing, A‐tailing, and ligation with Illumina‐compatible adapters. The ligation products were then PCR‐amplified to enrich the library. Amplified products were purified using the AMPure XP system (Beverly, USA). Library quality was assessed using the Agilent 5400 system (Agilent, USA), and concentrations were determined using qPCR, yielding a final library concentration of 1.5 nM. Qualified libraries were sequenced on the Illumina HiSeq platforms using both the Single‐End 150 bp (SE150) and Single‐End 250 bp (SE250) strategies.

### Shotgun sequencing of pooled large DNA fragments

For both DNA‐40.5kb‐DR (*R* = 1/4) and DNA‐40.5kb‐EM (*R* = 2/3), 0.14 μg of input plasmid DNA from each sample was diluted and mixed prior to library preparation. Library construction followed the same protocol as described above, including random fragmentation, end polishing, adapter ligation, and PCR amplification. The final libraries were quality‐checked using the Agilent 5400 system (Agilent, USA) and quantified by qPCR to a concentration of 1.5 nM. Qualified libraries were pooled and sequenced on the Illumina HiSeq platforms using the SE150 strategy.

### Data quality control

To ensure the reliability and accuracy of data analysis, quality control of the raw sequencing reads was performed using Fastp (v0.23.1) [57, 58]. Raw sequencing reads were subjected to standard quality control using the following criteria:

(i) Reads containing more than 10% unrecognizable bases (N) were removed.
(ii) Reads in which low‐quality bases (Phred quality ≤ 5) accounted for more than 50% of the total read length were removed.
(iii) Reads containing adapter sequences were also removed.

### Definitions of read types

Sequencing reads were classified into three categories according to a hierarchical alignment against multiple hidden references. Each type was defined based on its alignment behavior and characteristic error patterns (Figures S53, S55, S57, S59, and S64), as follows:

(i) **Type‐I reads:** Rapidly identified through correlation with the watermark reference; these reads exhibit relatively low error rates, with indel errors either absent or confined to the read ends.
(ii) **Type‐II reads:** Fail to align with the watermark reference but can be aligned to the scaffold reference; they typically have higher overall error rates, with indel errors enriched in the middle regions.
(iii) **Type‐III reads:** Fail to align with both the watermark and scaffold references but align to the regenerative reference; these reads usually originate from the gap regions of the scaffold reference and exhibit error rates and indel distributions similar to those of Type‐II reads.

### Fast data readout from NGS reads

A fast data recovery method was proposed, mainly consisting of sliding correlation and bit‐wise consensus. The data recovery workflow using NGS reads is divided into three steps:

(i) Sliding correlation between noisy reads and watermark sequences was used to identify read positions based on correlation peak values, and the aligned reads were grouped accordingly.
(ii) The corrupted codeword fragments were combined using bit‐wise majority voting to generate a reconstructed codeword containing both erasures and substitution errors.
(iii) Soft‐decision decoding was applied to correct the residual errors and recover the original data.

### Read identification via correlation to watermark reference

Read identification was performed using sliding correlation against the watermark reference. The sliding correlation procedures are as follows:

(i) The noisy reads were demapped into double‐layer bit sequences according to the mapping rule {A → (00), T → (01), G → (10), C → (11)}. In this process, the first bit of each pair was extracted to form the first layer, while the second bit formed the second layer.
(ii) The correlation values between the blurred watermark sequences $\underline{r}=\left\{{\underline{r}}_{1},{\underline{r}}_{2}\right\}$ and the ideal double‐layer watermark sequences $\underline{w}=\left\{{\underline{w}}_{1},{\underline{w}}_{2}\right\}$were computed bit by bit using a sliding window with a length of l bits. The correlation value $V_{i}$ between the sequencing reads and the ideal watermark sequence at the position i was calculated as follows:
(1) $$V_{i}=\sum_{j=0}^{j=l-1}{{\underline{r}}_{1}}^{\left(j\right)}\cdot {{\underline{w}}_{1}}^{\left(i+j\right)}+{{\underline{r}}_{2}}^{\left(j\right)}\cdot {{\underline{w}}_{2}}^{\left(i+j\right)},0\leq i\leq L-l$$
  where L and l denote the lengths of the ideal watermark sequence and the sequencing reads, respectively.
(iii) The correlation values $V_{0},V_{1},\cdots ,V_{L-l}$ at L−l+1 positions for each read were obtained by sliding correlation. By comparing the correlation values of each read at different positions, the correlation peak value $V_{\text{peak}}=\;\max(V_{0},V_{1},\cdots ,V_{L-l})$ and peak position were determined. The peak position indicated the starting location of the noisy reads on the watermarked DNA fragment, enabling precise read localization without the need for sequence assembly.

### Bit‐wise consensus and probability generation

After sliding correlation was used to determine the precise read position, bit‐wise consensus was applied to generate soft information for decoding. The method involves the following steps:

(i) Data fragments were combined bit by bit to reconstruct a probability consensus. If no consensus was reached, the position was marked as an erasure.
(ii) The probability consensus was superposed with the watermark sequence by exclusive OR (XOR) operation, and joint probability across multiple bits was calculated to recover the original sparse representation.
(iii) For erased positions, LLR was set to 0; otherwise, it was computed as $\log\left({b_{i}}^{1}/{b_{i}}^{0}\right)$, where ${b_{i}}^{1}$ and ${b_{i}}^{0}$ are the probabilities of bit 1 and bit 0 at position i, respectively.
(iv) The recovered LLRs were used as inputs for soft‐decision decoding.

### Data readout in a bootstrap manner from reads with indel errors

Bootstrap readout is defined as a progressive data recovery process that sequentially identifies sequencing reads through correlation and alignment, integrating each read type for error correction and decoding in a self‐driven manner.

(i) **Identification of low‐error reads for initial data recovery.**
  Noisy reads were first subjected to sliding correlation against the watermark reference to identify Type‐I reads, which typically exhibit low error rates. These reads were corrected using FBA, and the resulting symbol probabilities were used for soft‐decision decoding, enabling initial data recovery.
(ii) **Identification of indel‐containing reads for enhanced data recovery.**
  A scaffold reference was constructed by base‐wise majority voting across Type‐I reads. This scaffold was then used to identify additional reads containing indel errors, labeled as Type‐II reads. Soft information obtained from the FBA of Type‐I and Type‐II reads was integrated to refine soft‐decision decoding.
(iii) **Identification of gap‐filling reads for complete data recovery.**
  The decoded codeword obtained from the previous stage was sparsified and superposed with the watermark to form a regenerative reference, which was subsequently used to identify Type‐III reads located in scaffold gaps. Their FBA‐corrected outputs were merged with those from Type‐I and Type‐II reads to achieve complete data recovery.

### Read‐by‐read forward‐backward algorithm

To correct indel errors, the FBA on the HMMs was applied to each noisy read (Figure S26). The inferred soft information was used for soft‐decision LDPC decoding. The procedure is described as follows (see Supplementary Note 5 for details):

(i) The noisy reads $\underline{r}$ with length of L were demapped into the upper and lower blurred watermark sequences ${\underline{r}}_{1}$ and ${\underline{r}}_{2}$, which serve as the observation vectors of the HMMs.
(ii) The watermark fragments ${\underline{w}}_{1}$ and ${\underline{w}}_{2}$ were extracted from the complete ideal watermarks, based on the position determined by correlation or alignment.
(iii) The recursive computation of forward probabilities $F_{j}(y)$ and backward probabilities $B_{j}(y)$ was performed for each state *y* at position index *j*. The intermediate probability was then computed, which was represented as $M_{k}(y,d_{i})$ at position k within one symbol, where $d_{i}\in GF\left(q\right)$, and *q* denotes the alphabet size of the encoding field.
(iv) The forward, backward and intermediate probabilities were integrated to derive the soft information *P* for each sparsified symbol with a length of *m* bits. The probability matrix of the symbols for each read was $v=\{P_{1},P_{2},\cdots ,P_{N_{L}}\}$, where $P_{j}$ denotes the probability distribution of the *j*‐th symbol, and *N*_*L*_ = *L*/*m* represents the total number of symbols within a read.

### Probability‐wise consensus and soft‐decision decoding

To improve symbol‐level accuracy in the presence of overlapping aligned reads, we employed a symbol probability merging strategy. The detailed procedure is described as follows:

(i) The symbol probability distributions $V=\{v_{1},v_{2},\cdots ,v_{c}\}$ for *c* reads were generated by the FBA, where each $v_{i}\in {\mathbb{R}}^{N_{L}\times 2^{m}}$ represents the symbol probability matrix of the *i*th read, *m* is the symbol length, and *N*_s_ is the number of encoded symbols. The probabilities at the same position were sequentially multiplied and normalized to generate consensus probability $v_{\text{cons}}=\{P_{1}^{\prime },P_{2}^{\prime },\cdots ,P_{N_{s}}^{\prime }\}$, each $P_{j}^{\prime }=(P_{j,0}^{\prime },P_{j,1}^{\prime },\cdots ,P_{j,2^{m}-1}^{\prime })$ denotes the probability distribution of the *j*th symbol, where $\sum_{k=0}^{2^{m}-1}P_{j,k}^{\prime }=1$.
(ii) Positions that remained uncovered after merging were assigned a probability of 0.5, indicating erasures in the consensus.
(iii) According to the symbol‐to‐bit mapping defined by the sparsification table, each merged symbol probability $P_{j}^{\prime }=(P_{j,0}^{\prime },P_{j,1}^{\prime },\cdots ,P_{j,2^{m}-1}^{\prime })$ was converted into a bit‐wise marginal probability: $b_{j}^{0}=\{b_{j,1}^{0},b_{j,2}^{0},\cdots ,b_{j,m}^{0}\}$ and $b_{j}^{1}=\{b_{j,1}^{1},b_{j,2}^{1},\cdots ,b_{j,m}^{1}\}$, where $b_{i}^{0}$ and ${b_{i}}^{1}$ denote the marginal probability of the i‐th bit being 0 and 1, respectively.
(iv) The consensus bit‐wise posterior probabilities were converted into LLRs as $S=\;\log(b^{1}/b^{0})$, where $b^{1}=\{b_{1}^{1},b_{2}^{1},\cdots ,b_{N_{S}}^{1}\}$ and $b^{0}=\{b_{1}^{0},b_{2}^{0},\cdots ,b_{N_{S}}^{0}\}$ denote the probability that each of the *N*_s_ symbols corresponds to bit 1 and bit 0, respectively.
(v) The consensus LLRs were fed into the LDPC decoder for soft‐decision decoding, which corrected the residual errors.

### Read identification via alignment to scaffold reference

Reads that could not be aligned to the watermark reference were subject to scaffold‐reference alignment (Figure S29). The procedure is described as follows:

(i) A scaffold reference was constructed from Type‐I reads by majority voting. Positions lacking sequencing data were marked as *‘X’*.
(ii) Each correlation‐failed read was segmented into *N* equal‐length subsequences (*N* = 2 in practice).
(iii) Each subsequence was independently aligned to the scaffold reference. An alignment was considered successful if the normalized edit distance $ED_{\mathrm{norm}}$ was below a threshold *T* ^1^ (*T* ^1^ = 0.2 in practice). Here, $ED_{\mathrm{norm}}=ED/L_{\mathrm{aligned}}$, where ED is edit distance and $L_{\mathrm{aligned}}$ is the aligned length.
(iv) If any subsequence was successfully aligned to the scaffold reference, the corresponding read was classified as a Type‐II read.

### Read identification via alignment to regenerative reference

To identify reads that fill codeword gaps, a regenerative reference was constructed from decoding results using the following steps:

(i) The decoded codeword was sparsified by converting every 4 bits into 5 bits. The sparsified codeword was bit‐wise superimposed with equal‐length watermark sequence to generate the regenerative reference.
(ii) Reads that failed to align with the scaffold reference were demapped into dual‐layer bit sequences.
(iii) The dual‐layer bit sequences were aligned to the dual‐layer regenerative reference.
(iv) Reads whose starting positions differed by no more than 10 between the two layers were considered successfully aligned to the regenerative reference and were classified as Type‐III reads.

### Data readout in a bootstrap manner from nanopore reads

For nanopore reads, direct correlation to the watermark reference is impractical (raw error rate of ~5%). Therefore, our readout framework incorporates a read segmentation process, summarized as follows:

(i) Long reads suffering from severe indel errors (total length *L*) were split into shorter segments of length *l*. Segments shorter than a predefined threshold *l*₁ were excluded from the data recovery process (*l*₁ = 40 in practice).
(ii) Correlation was performed between fragments longer than the threshold *l*₂ and the watermark reference to identify Type‐I reads (*l*₂ = 100 in practice). Empirically, fragments shorter than 100 nt exhibited markedly reduced correlation accuracy (Figure 2F). The correlation threshold was dynamically adjusted according to the fragment length, defined as the product of the read length and a scaling factor. Based on statistical analysis, the scaling factor was determined to be 0.32 (Figure S13).
(iii) The scaffold and regenerative references were progressively constructed to identify Type‐II and Type‐III reads, respectively, enabling hierarchical read integration. The read‐by‐read FBA was then applied to correct indel errors, and the probabilistic outputs were merged for soft‐decision decoding.

### Error rate computation of sequencing reads

The error rate was computed as the edit distance between each noisy read and the reference sequence, normalized by the aligned length (Algorithm 3). Both the edit distance and alignment length were obtained using Edlib (v1.2.6) [53] under local alignment mode. For circular plasmid sequences, the reference sequence was duplicated and concatenated to allow full‐length local alignments. The error rate was calculated as *P*_err_ = (*N*_ins_ + *N*_del_ + *N*_sub_)/*L*_aligned_. Here, *N*_ins_, *N*_del_, and *N*_sub_ denote the numbers of insertion, deletion, and substitution errors, respectively, and *L*_aligned_ is the aligned sequence length. Only reads with error rates below a predefined threshold were included in the overall error rate estimation. In practice, this threshold was empirically set to 0.2.

### Error modeling and read simulation

In silico simulation experiments were conducted to further evaluate the performance of the readout framework under diverse error conditions. Error modeling was based on empirical statistics derived from real sequencing data. Substitution errors were assigned according to base‐quality score distributions, and a quality score profile was constructed using the ART profiler (v2.5.8) [59]. Insertion and deletion errors were simulated following empirical indel distributions obtained from training datasets.

During simulation, single‐end reads of 150 nt were generated with predefined insertion, deletion, and substitution error rates. Two representative error profiles were simulated:

(i) Profile 1—Overall error rate of 0.6%, with *P*_i_: *P*_d_: *P*_s_ = 10: 5: 2, mimicking indel‐rich error patterns commonly observed in Ion Torrent platforms.
(ii) Profile 2—Overall error rate of 1.2%, with *P*_i_: *P*_d_: *P*_s_ = 1: 1: 2, representing a higher‐indel scenario, where the indel error rate was approximately 0.6%.

All simulations were performed on a server equipped with Intel® Xeon® Gold 5220 R@2.20 GHz (48 cores) and 256 GB of RAM. The simulated reads were subsequently used in data readout experiments to assess the recovery performance of the proposed framework. For each simulated dataset, 200 independent data recovery trials were performed. The BER is presented with error bars denoting mean ± standard deviation (mean ± SD) across the 200 trials, and the recovery rate is defined as the fraction of trials with successful recovery.

**Video S1:** Workflow of fast readout in a bootstrap manner using hidden references for DNA data storage.

## AUTHOR CONTRIBUTIONS

**Weigang Chen**: Conceptualization; writing—original draft; writing—review and editing; funding acquisition; project administration; resources; supervision. **Shuang Liu**: Data curation; software; validation; visualization; writing—original draft; writing—review and editing. **Quan Guo**: Writing—original draft; writing—review and editing; visualization; software; data curation; validation. **Rui Qin**: Investigation; writing—original draft; writing—review and editing. **Qi Ge**: Software; writing—original draft; writing—review and editing. **Tingting Qi**: Visualization; writing—original draft; writing—review and editing. **Yingjin Yuan**: Conceptualization; funding acquisition; project administration; supervision; writing—review and editing. All authors have read the final manuscript and approved it for publication.

## CONFLICT OF INTEREST STATEMENT

Weigang Chen, Shuang Liu, Quan Guo, Qi Ge, and Tingting Qi have applied for two Chinese patents related to readout methods based on multiple‐fold hidden reference sequences for large‐fragment DNA data storage (ZL 2025106927508, ZL 2024115923893). The remaining authors declare no conflicts of interest.

## ETHICS STATEMENT

No animals or humans were involved in this study.

## Supporting information


**Figure S1.** Workflow of readout in a bootstrap manner for large DNA fragments using multiple‐fold hidden references.
**Figure S2.** Data recovery framework in a bootstrap manner using multiple‐fold hidden references.
**Figure S3.** Comparison of the proposed assembly‐free readout with traditional assembly‐based methods.
**Figure S4.** Experimental verification of the proposed framework.
**Figure S5.** Simulation verification of the proposed framework.
**Figure S6.** Overview of the coding schemes and sparsification rules.
**Figure S7.** Schematic diagrams of the two data‐carrying plasmids.
**Figure S8.** Raw error rates of real sequencing reads.
**Figure S9.** Base‐coverage distribution of Illumina reads.
**Figure S10.** Comparison with other DNA data storage schemes.
**Figure S11.** Correlation‐based read identification under different types of noise.
**Figure S12.** Distribution of correlation peak values for data‐carrying and interfering reads.
**Figure S13.** Bit error rate distribution before decoding under different correlation thresholds.
**Figure S14.** Proportions of data‐carrying and interfering reads in sequencing data.
**Figure S15.** ROC curves of correlation‐based filtering under different thresholds across datasets.
**Figure S16.** PR curves of correlation‐based filtering under different thresholds across datasets.
**Figure S17.** Data recovery under host genome interference.
**Figure S18.** Accuracy of correlation‐based classification of data‐carrying and interfering reads.
**Figure S19.** Read utilization of sliding correlation.
**Figure S20.** Proportion and error characteristics of data‐carrying reads.
**Figure S21.** Large DNA encoding workflow used in simulation.
**Figure S22.** Impact of error profiles on positioning accuracy and read utilization.
**Figure S23.** Data recovery performance using 250‐bp NGS reads.
**Figure S24.** Distinguishing cross‐fragment interference in pooled‐fragment experiments using watermark‐based correlation.
**Figure S25.** Recovery performance under pooled sequencing.
**Figure S26.** Workflow of the read‐by‐read forward‐backward algorithm.
**Figure S27.** Base erasure rate and base substitution error rate of the scaffold reference (simulated data, raw error rate = 0.6%).
**Figure S28.** Base erasure rate and base substitution error rate of the scaffold reference (simulated data, raw error rate = 1.2%).
**Figure S29.** Workflow of the partial alignment method.
**Figure S30.** Evaluation of the probability generation with FBA.
**Figure S31.** Evaluation of partial alignment on DNA‐40.32kb‐ER‐Sim‐1 (*R* = 1/2).
**Figure S32.** Evaluation of partial alignment on DNA‐40.32kb‐ER‐Sim‐2 (*R* = 1/2).
**Figure S33.** Evaluation of data recovery using different types of reads and soft‐decision information generation schemes (raw error rate = 0.6%).
**Figure S34.** Evaluation of data recovery using different types of reads and soft‐decision information generation schemes (raw error rate = 1.2%).
**Figure S35.** Mapping positions of simulated reads on the large‐fragment DNA.
**Figure S36.** Error rates of the regenerative reference under varying sequencing coverages (raw error rate = 0.6%).
**Figure S37.** Error rates of the regenerative reference under varying sequencing coverages (raw error rate = 1.2%).
**Figure S38.** Distribution of indel error positions across types of reads (raw error rate = 0.6%).
**Figure S39.** Distribution of indel error positions across types of reads (raw error rate = 1.2%).
**Figure S40.** Error distribution across reconstructed codewords using different types of reads.
**Figure S41.** Evaluation of gap‐filling scheme on DNA‐40.5kb‐DR‐Sim‐1 (*R* = 1/4).
**Figure S42.** Evaluation of gap‐filling scheme on DNA‐40.32kb‐ER‐Sim‐1 (*R* = 1/2).
**Figure S43.** Evaluation of gap‐filling scheme on DNA‐40.5kb‐EM‐Sim‐1 (*R* = 2/3).
**Figure S44.** Evaluation of gap‐filling scheme on DNA‐40.5kb‐MC‐Sim‐1 (*R* = 5/6).
**Figure S45.** Evaluation of gap‐filling scheme on DNA‐40.5kb‐DR‐Sim‐2 (*R* = 1/4).
**Figure S46.** Evaluation of gap‐filling scheme on DNA‐40.32kb‐ER‐Sim‐2 (*R* = 1/2).
**Figure S47.** Evaluation of gap‐filling scheme on DNA‐40.5kb‐EM‐Sim‐2 (*R* = 2/3).
**Figure S48.** Evaluation of gap‐filling scheme on DNA‐40.5kb‐MC‐Sim‐2 (*R* = 5/6).
**Figure S49.** Evaluation of gap‐filling scheme on DNA‐20kb‐RB‐Sim‐1 (*R* = 0.93).
**Figure S50.** Evaluation of gap‐filling scheme on DNA‐20kb‐RB‐Sim‐2 (*R* = 0.93).
**Figure S51.** Workflow of nanopore sequencing with read segmentation and dynamic‐threshold correlation.
**Figure S52.** Read length distribution and error rates for DNA‐40.5kb‐DR‐ONT‐1 (*R* = 1/4, raw error rate = 5.2%).
**Figure S53.** Evaluation of gap‐filling scheme on DNA‐40.5kb‐DR‐ONT‐1 (*R* = 1/4, raw error rate = 5.2%).
**Figure S54.** Read length distribution and error rates for DNA‐40.5kb‐EM‐ONT‐1 (*R* = 2/3, raw error rate = 4.7%).
**Figure S55.** Evaluation of gap‐filling scheme on DNA‐40.5kb‐EM‐ONT‐1 (*R* = 2/3, raw error rate = 4.7%).
**Figure S56.** Read length distribution and error rates for DNA‐40.5kb‐DR‐ONT‐2 (*R* = 1/4, raw error rate = 4.1%).
**Figure S57.** Evaluation of gap‐filling scheme on DNA‐40.5kb‐DR‐ONT‐2 (*R* = 1/4, raw error rate = 4.1%).
**Figure S58.** Read length distribution and error rates for DNA‐40.5kb‐EM‐ONT‐2 (*R* = 2/3, raw error rate = 3.5%).
**Figure S59.** Evaluation of gap‐filling scheme on DNA‐40.5kb‐EM‐ONT‐2 (*R* = 2/3, raw error rate = 3.5%).
**Figure S60.** Read length distribution and error profile of unused reads from DNA‐40.5kb‐EM‐ONT‐1 (*R* = 2/3).
**Figure S61.** Base erasure rate and base substitution error rate of the scaffold reference (real Illumina data).
**Figure S62.** Error rates of the regenerative reference under varying sequencing coverages (real Illumina data).
**Figure S63.** Proportions of different read types used in data recovery (real Illumina data).
**Figure S64.** Distribution of indel error positions and error rates across types of reads (real Illumina data).
**Figure S65.** Data recovery using different types of reads (real Illumina data).
**Figure S66.** Evaluation of the accelerated readout scheme using Minimap2 on DNA‐40.5kb‐DR‐ONT‐1 (*R* = 1/4, raw error rate = 5.2%).
**Figure S67.** Evaluation of the accelerated readout scheme using Minimap2 on DNA‐40.5kb‐EM‐ONT‐1 (*R* = 2/3, raw error rate = 4.7%).

**Table S1.** Comparison of large‐fragment DNA data storage schemes.
**Table S2.** Summary of data files encoded in the designed large‐fragment DNA.
**Table S3.** Real sequencing datasets and experimental setup in this work.
**Table S4.** Comparison between the proposed method and the assembly method.
**Table S5.** Minimum coverage required for diverse sequencing error profiles.
**Table S6.** Classification metrics with 95% confidence intervals for correlation‐based filtering.
**Table S7.** Raw error rates of data‐carrying reads.
**Table S8.** Recovery performance with 150‐nt NGS reads.
**Table S9.** Recovery performance with 250‐nt NGS reads.
**Table S10.** Independent recovery performance across datasets (150‐nt reads).
**Table S11.** Independent recovery performance across datasets (250‐nt reads).
**Table S12.** Recovery performance under pooled sequencing.
**Table S13.** Hardware and software configurations of the computational environment.
**Table S14.** Runtime and peak RAM (single‐thread) using Illumina data.
**Table S15.** Runtime and peak RAM (single‐thread) using nanopore sequencing data.
**Table S16.** Runtime and peak RAM (DNA‐40.5kb‐DR‐Sim‐1, *R* = 1/4, raw error rate = 0.6%, single‐thread).
**Table S17.** Runtime and peak RAM (DNA‐40.32kb‐ER‐Sim‐1, *R* = 1/2, raw error rate = 0.6%, single‐thread).
**Table S18.** Runtime and peak RAM (DNA‐40.5kb‐EM‐Sim‐1, *R* = 2/3, raw error rate = 0.6%, single‐thread).
**Table S19.** Runtime and peak RAM (DNA‐40.5kb‐MC‐Sim‐1, *R* = 5/6, raw error rate = 0.6%, single‐thread).
**Table S20.** Runtime and peak RAM (DNA‐20kb‐RB‐Sim‐1, *R* = 0.93, raw error rate = 0.6%, single‐thread).
**Table S21.** Recovery performance of the recovery pipeline using Minimap2.

## Data Availability

Raw sequencing reads of four large DNA fragments obtained from Illumina platforms are available in the Sequence Read Archive under accession number PRJNA1304189 (https://www.ncbi.nlm.nih.gov/bioproject/PRJNA1304189). Raw sequencing reads generated from two independent nanopore sequencing and pooled‐fragment experiments are available in the Sequence Read Archive under accession number PRJNA1357870 (https://www.ncbi.nlm.nih.gov/bioproject/PRJNA1357870). The original data files, encoded codewords, encoded sequences, pseudo‐noise sequences, and raw sequencing reads are publicly available via Zenodo at https://doi.org/10.5281/zenodo.17545286. The source code, along with the Docker‐based environment, is available in the GitHub repository (https://github.com/dna-storage-lab/Bootstrap-readout-using-hidden-references). The specific version of the code associated with this publication is archived in Zenodo and is accessible via 10.5281/zenodo.17545118. Supplementary materials (figures, tables, graphical abstract, slides, videos, Chinese translated version, and update materials) may be found in the online DOI or iMeta Science (http://www.imeta.science).
