# Supplementary material for: Fast bootstrap and reliable readout using hidden references for DNA data storage
Source: Imeta. 2026 Jan 11;5(1):e70105. doi: 10.1002/imt2.70105 (PMC13726579; doi:10.1002/imt2.70105)
Supplement: Supplementary file 1 [file IMT2-5-e70105-s002.pdf]

Supplementing information to

Fast bootstrap and reliable readout using hidden references for DNA data storage

Running Title: Bootstrap readout of DNA data storage

Weigang Chen[1,2,3,*,#], Shuang Liu[1,#], Quan Guo[1,#], Rui Qin[1], Qi Ge[1], Tingting Qi[1], Yingjin Yuan[2,3,*]

[1]School of Microelectronics, Tianjin University, Tianjin 300072, China

[2]State Key Laboratory of Synthetic Biology, Tianjin University, Tianjin 300072, China

[3]Frontiers Science Center for Synthetic Biology (Ministry of Education), School of Synthetic Biology and Biomanufacturing, Tianjin University, Tianjin 300072, China

[#]These authors contributed equally: Weigang Chen, Shuang Liu, Quan Guo

[*]Correspondence: chenwg@tju.edu.cn (Weigang Chen), yjyuan@tju.edu.cn (Yingjin Yuan)

Supplementary Note 1: Encoding and decoding of watermark-superimposed LDPC codes

Modern data storage systems, including DNA-based platforms, demand exceptionally high reliability in data retrieval. To this end, error-correcting codes (ECCs) are routinely employed.

(1) Motivation for watermark-superimposed LDPC code design

Data readout from massive, unordered sequencing reads typically requires overlap-based alignment, which is complicated by diverse sequencing errors. To address this problem, we constructed large DNA fragments using watermark superposition coding. In this scheme, watermark sequences are embedded as concealed components within the DNA payloads and serve two critical purposes. First, function as hidden references that facilitate the rapid alignment of noisy sequencing reads. Second, they enable the inference of soft information for sparse codewords through hidden Markov models (HMMs) [1−4].

(2) Encoding of watermark-superimposed LDPC codes

Digital files were initially encoded using low-density parity-check (LDPC) codes to produce binary codewords. To improve robustness against burst errors and enhance bit dispersion, the codewords were interleaved. These interleaved sequences were then sparsified by mapping every 4 bits to 5 bits using “sparsification table 1”. This transformation reduced the theoretical maximum correlation peak from $2L$ to $2L(1 - 2S)$, where L denotes the read length and S represents the sparsity factor. The sparsified codewords were subsequently bit-wise XOR operation with watermark sequences of equal length. Finally, the resulting bitstream was transcoded into a DNA sequence by converting each pair of bits into a nucleotide according to the rule {(00)→A, (01)→T, (10)→G, (11)→C}.

The proposed framework was evaluated through *in vivo* experiments and *in silico* simulations across multiple code rates (Figures S4 and S5), enabling a systematic assessment of redundancy and robustness. For *in vivo* validation, various digital files were encoded with rate-1/4, rate-1/2, rate-2/3, and rate-5/6 codes. For instance, Tagore’s poem *Dreams* was encoded with a code rate of 1/4, yielding a 40,500-bp DNA sequence. Similarly, the emblem of Tianjin University was encoded with a code rate of 2/3, also producing a 40,500-bp payload. In addition, the image files *Milk Coronet* and *Earthrise* were encoded with rates of 5/6 and 1/2 [5], resulting in sequences of 40,500 bp and 40,320 bp, respectively [6]. In

addition to the four experimental code rates, this study also included LDPC (32,000, 29,760) with a code rate of 0.93 in simulations, aiming to comprehensively assess the error-correcting capabilities of binary LDPC codes under high-rate encoding conditions.

(3) Decoding of watermark-superimposed LDPC codes

Modern error correction codes primarily rely on soft-decision decoding, which leverages probabilistic inputs to improve decoding accuracy. In this study, we developed a soft-decision decoding scheme tailored to watermark-superimposed codewords. Based on the progressive identification of sequencing reads, we designed two complementary methods for generating soft-decision information.

The first method produces bit-wise probabilistic inputs for rapid soft-decision decoding. This approach is optimized for reads with low error rates, allowing fast and efficient reconstruction. The second method applies a soft-decision forward-backward algorithm (FBA) to enhance the handling of reads affected by insertion and deletion (indel) errors. This strategy enables effective correction of more complex sequencing distortions and improves the overall decoding robustness.

Supplementary Note 2: Threshold selection criterion for correlation-based filtering

(1) Notation and threshold selection by minimizing BER

For each read $i = 1, \ldots, N$, we have:

- Ground-truth label $y_i \in \{0,1\}$, obtained from alignment (1 = target data-carrying read; 0 = non-target/contaminant).
- A continuous score s_i (the normalized watermark-correlation peak; larger means more likely positive).

Given a decision threshold τ, the classifier is:

$$\hat{y}_i(\tau) = \begin{cases} 1, & s_i \geq \tau, \\ 0, & s_i < \tau \end{cases}$$

The optimal threshold is determined by minimizing the consensus bit error rate (BER) before decoding (Figures 2D and S13). The BER at threshold τ as:

$$\text{BER}(\tau) = \frac{\text{\#bit errors before LDPC decoding at } \tau}{\text{\#bits compared at } \tau}.$$

Operating threshold (selected on a validation set):

$$\tau = \arg\min_{\tau} \text{BER}(\tau).$$

(2) Confusion matrix and point metrics

In our context, positive samples ($y_i = 1$) correspond to data-carrying reads, whereas negative samples ($y_i = 0$) correspond to interfering reads filtered out by the correlation-based method. To evaluate the rationality of threshold selection, for any τ, define the confusion-matrix counts:

$$TP(\tau) = \sum_i \mathbf{1}\{y_i = 1, \hat{y}_i(\tau) = 1\},$$
$$FP(\tau) = \sum_i \mathbf{1}\{y_i = 0, \hat{y}_i(\tau) = 1\},$$
$$TN(\tau) = \sum_i \mathbf{1}\{y_i = 0, \hat{y}_i(\tau) = 0\},$$
$$FN(\tau) = \sum_i \mathbf{1}\{y_i = 1, \hat{y}_i(\tau) = 0\}.$$

Here, *TP* (true positives) denotes correctly identified data-carrying reads; *FP* (false positives) denotes interfering reads misclassified as data-carrying; *TN* (true negatives) represents correctly excluded interferents; and *FN* (false negatives) denotes data-carrying reads incorrectly filtered as interferents. Let $P = TP + FN$ (data-carrying reads), $N = TN + FP$ (interfering reads). At the optimal threshold τ = τ*, we report the following point metrics:

$$\text{TPR (Sensitivity)} = \frac{TP}{TP + FN}$$
$$\text{TNR (Specificity)} = \frac{TN}{TN + FP}$$
$$\text{FPR} = \frac{FP}{FP + TN} = 1 - \text{Specificity}$$
$$\text{FNR} = \frac{FN}{TP + FN} = 1 - \text{Sensitivity}$$
$$\text{Precision} = \frac{TP}{TP + FP}$$
$$\text{Recall=} = \frac{TP}{TP + FN}$$

These point metrics describe the classifier's intrinsic detection ability and are sensitive to class proportions. To provide an overall, class-balanced evaluation, we further analyzed the

F1-score and Youden's J.

$$\mathrm{F1} = \frac{2 \times \mathrm{Precision} \times \mathrm{Sensitivity}}{\mathrm{Precision} + \mathrm{Sensitivity}}$$

$$\mathrm{Youden's\ J} = \mathrm{Sensitivity} + \mathrm{Specificity} - 1$$

All performance metrics were reported with 95% confidence intervals. For Sensitivity, Specificity, FPR, FNR, Precision, and Accuracy, the Wilson score interval was used to estimate the 95% confidence bounds. For F1 and Youden's J, confidence intervals were obtained using the bootstrap percentile method with 10,000 resamples.

(3) ROC/PR curves for selectivity check

To assess the rationality of the correlation threshold, we plotted ROC and PR curves by sweeping τ over the correlation scores: ROC curve: $x = \mathrm{FPR}(\tau),\ y = \mathrm{TPR}(\tau)$, PR curve: $x = \mathrm{Recall}(\tau),\ y = \mathrm{Precision}(\tau)$.

Supplementary Note 3: Evaluation of correlation performance across coding sparsity and error profiles

To enable rapid read positioning, the proposed method applies sliding correlation between noisy reads and embedded watermark sequences. We evaluated how correlation performance is influenced by two key factors: coding sparsity and sequencing error characteristics.

First, we assessed the effect of different coding sparsity ratios. Sparsification can reduce interference and preserve correlation performance. However, lower sparsity ratios enhance positioning accuracy at the cost of reduced information density. Therefore, a trade-off exists between information density and correlation robustness. Second, we evaluated the impact of distinct sequencing error profiles, examining both positioning accuracy and read utilization under different insertion, deletion, and substitution ratios.

(1) Evaluation metrics for sliding correlation

The performance of the sliding correlation algorithm was evaluated based on two key metrics: positioning accuracy and read utilization.

Positioning accuracy measures how closely the estimated position of a read matches its true location on the DNA reference. The true position of each read (P_{align}) was determined by standard sequence alignment. The predicted position (P_{corr}) was obtained from the sliding

correlation peak. A read was considered successfully positioned when the absolute difference between the predicted and true positions was less than a predefined threshold:

$$|P_{\text{align}} - P_{\text{corr}}| < T_{\text{position}}$$

where T_{position} is a predefined threshold reflecting the tolerance for positional deviation. In this study, we set $T_{\text{position}} = 1$, which imposes a strict criterion for successful positioning.

Read utilization was defined as the proportion of data-carrying reads that were successfully identified by sliding correlation. Ground-truth labels for evaluating these metrics were derived from reference-guided alignment.

(2) Evaluation of coding sparsity on correlation performance

To evaluate correlation performance, we constructed large DNA fragments by encoding digital data using LDPC codes with a code rate of 2/3. Two sparsification modes were employed to examine their effects (Figure S6B). In the first mode, codewords were sparsified by converting every 4 bits into 5 bits using "sparsification table 1". In the second mode, every 4 bits were converted into 6 bits using "sparsification table 2". The resulting sparse codewords were then bit-wise XOR operation with watermark sequences of equal length, followed by 2-bit-to-1-base transcoding to generate the final DNA payloads.

Sequencing reads were simulated from the constructed payloads using the ART simulator, configured for the NGS platform [7]. Single-end reads of varying lengths (50–250 nt) were generated at a fixed total error rate of 0.6%, using quality profiles derived from real NGS datasets. These reads were used to assess both the accuracy of read positioning and the overall read utilization.

(3) Evaluation of sequencing error profiles on correlation performance

To investigate the influence of sequencing error characteristics, simulated NGS reads were generated with ART using DNA-40.5kb-EM (R = 2/3). Specifically, substitution errors were assigned according to base quality scores derived from real sequencing data. Insertion and deletion errors were simulated from empirical distributions derived from the training data. A comparative analysis was conducted using two representative error models:

- Model 1: P_i: P_d: P_s = 10:5:2, mimicking indel-rich profiles commonly observed in Ion Torrent–like platforms.

- Model 2: P_i: P_d: P_s = 1:1:2, representing a more balanced indel-to-substitution distribution.

Here, P_i, P_d, and P_s denote the insertion, deletion, and substitution error rates, respectively, with the total base error rate $P_{err}=P_i+P_d+P_s$. Reads were simulated at total error rates of 0.4%, 0.6%, 0.8%, 1.0%, and 1.2%.

Supplementary Note 4: Assembly-based readout of large DNA fragments

The de Bruijn graph approach is commonly used for short-read assembly, where reads are converted into *k*-mers, which are error-corrected and simplified to generate contigs, followed by scaffold construction. We used Velvet (v1.2.09) [8] as the baseline tool to perform assembly-based recovery on real sequencing datasets. The baseline pipeline comprises three main steps:

(1) Contig assembly with Velvet

Velvet was used to construct a de Bruijn graph over a range of k-mer values and assemble short reads into contigs. The assembly with the longest contig was selected as the baseline output for payload localization and data recovery. In our experiments, we used single-end reads, scanned k-mers from 25 to 33 with a step of 2, and set the minimum contig length to 5000 nt. The following commands were used to implement the contig assembly:

```
velveth_de ${assembly_dir} 25,33,2 -short -fastq ${FASTQ}
velvetg_de ${assembly_dir} -cov_cutoff auto -min_contig_lgth 5000
```

(2) Payload localization

To determine the boundaries of the payload region, Edlib was used to align contigs to the vector reference sequences [9]. Given the circular structure of the plasmid, the reference sequences were defined as ideal vector fragments of 100 nt in length, located adjacent to both ends of the designed payload region. If the edit distance between a contig and the reference sequence meets a preset threshold, the payload region within the contig is trimmed.

(3) Base-wise consensus and decoding

Based on the alignment start positions, the trimmed contigs were combined to generate a consensus sequence through base-wise majority voting. The original data were recovered through iterative LDPC decoding.

Supplementary Note 5: Details of the forward-backward algorithm for indel correction

(1) Model definition

In DNA data storage systems, synthesis and sequencing processes inevitably introduce indel errors, leading to base-position drift that can severely affect data recovery. To model this process using HMMs, let P_i, P_d, and P_s denote the insertion, deletion, and substitution probabilities, respectively. Considering the sparsified codewords and channel substitutions, define the effective substitution probability P_f as

$$P_f = f(1-P_s)+(1-f)P_s$$

where f is average sparsity of codewords.

We defined the hidden state x_j as the drift variable at position j, representing the cumulative difference between the numbers of inserted and deleted bases prior to transmission of symbol t_j. The hidden state variable x_j takes discrete values from the finite set

$$X = \{-x_{\max},\ldots,-1,0,1,\ldots,x_{\max}\},$$

where $x_{\max}$ is the maximum drift, bounded by

$$x_{\max} = \sqrt{\frac{NP_d}{1-P_d}}$$

where N is the length of the raw sequencing read. This bound is introduced to limit the model complexity.

(2) Initialization of forward and backward probabilities

The initial forward and backward probabilities are defined as follows. For forward probability initialization, the prior probabilities at position t_0 were initialized as:

$$F_0(x) = \begin{cases} 1, x = 0 \\ 0, others \end{cases}$$

where $F_0(x)$ denotes the initial forward probability of drift state x, and $N_x = 2x_{\max} + 1$ is the total number of hidden drift states. This corresponds to a uniform prior distribution over all feasible drift states at the beginning of decoding.

For backward initialization, let L_t and L_r denote the lengths of the transmitted and received sequences, respectively. The terminal backward probabilities are defined as:

$$B_{L_t}(x)=\begin{cases}1, & \text{if } L_\mathrm{r}=L_\mathrm{t} \text{ and } x=0,\\ \dfrac{1}{x_{\max}}, & \text{if } L_\mathrm{r}<L_\mathrm{t} \text{ and } -x_{\max}\le x<0,\\ \dfrac{1}{x_{\max}}, & \text{if } L_\mathrm{r}>L_\mathrm{t} \text{ and } 0<x\le x_{\max}.\end{cases}$$

This initialization assumes a uniform distribution across feasible drift ranges, given the observed read length.

(3) Transition probabilities

Transition probabilities represent the likelihood of moving from state $x_i = a$ to state $x_{i+1} = b$, defined as

$$P_{a,b}=\begin{cases}P_\mathrm{d}, & b=a-1\\ \alpha_I P_\mathrm{i} P_\mathrm{d}+P_\mathrm{t}, & b=a\\ \alpha_I (P_\mathrm{i})^{b-a+1}P_\mathrm{d}+\alpha_I (P_\mathrm{i})^{b-a}P_\mathrm{t}, & a<b<a+I\\ \alpha_I (P_\mathrm{i})^{I}P_\mathrm{t}, & b=a+I\\ 0, & \mathit{others}\end{cases}$$

where $\alpha_I = 1/(1-(P_\mathrm{i})^I)$, and I denotes the maximum length of consecutive insertion errors ($I = 2$ in practice). P_t denotes the probability of correct transmission, defined as $P_\mathrm{t} = 1 - P_\mathrm{i} - P_\mathrm{d} - P_s$. The parameters P_i, P_d, and P_s, as well as I, were estimated from statistics of sequencing data generated in our preliminary experiments.

(4) Emission probabilities

Emission probabilities represent the likelihood of observing an output sequence u when the hidden state transitions from $x_j = a$ to $x_{j+1} = b$. The emission probability is defined as

$$Q_{j,a,b}(u)=P(u|\ x_j=a, x_{j+1}=b, w_j),$$

and is expressed as

$$Q_{j,a,b}(u)=\begin{cases}\dfrac{\alpha_I (P_\mathrm{i})^{b-a+1}\left(\frac{1}{2}\right)^{b-a+1}P_\mathrm{d}+\alpha_I (P_\mathrm{i})^{b-a}\left(\frac{1}{2}\right)^{b-a}P_\mathrm{t}(1-P_\mathrm{f})}{P_{a,b}}, & u^*=w_j,\\ \dfrac{\alpha_I (P_\mathrm{i})^{b-a+1}\left(\frac{1}{2}\right)^{b-a+1}P_\mathrm{d}+\alpha_I (P_\mathrm{i})^{b-a}\left(\frac{1}{2}\right)^{b-a}P_\mathrm{t}P_\mathrm{f}}{P_{a,b}}, & u^*\ne w_j,\end{cases}$$

where $u^* = u_{b-a+1}$ is the received bit associated with the watermark w_j, and

$$\alpha_I = \frac{1}{1-(P_{\mathrm{i}})^I}$$

is the normalization factor ensuring that the transition probabilities sum to one. The emission probability function $Q_{j,a,b}(u)=1$ satisfies the normalization constraint:

$$\sum_u Q_{j,a,b}(u)=1$$

(5) Stopping rules

The stopping rule of the FBA is defined with respect to the complete DNA read. The algorithm performs one full forward pass through the observed read to compute the cumulative likelihoods of all feasible drift states, followed by one full backward pass to propagate probabilities in reverse. The iteration terminates after both passes are completed, and the posterior probabilities are then obtained by combining the forward and backward metrics for each drift state.

Supplementary Note 6: Partial-length alignment guided by scaffold reference

A scaffold reference was constructed to position reads with indel errors, especially those reads with indel errors occurring in the middle region. Since the scaffold reference contained both erasure and substitution errors, direct alignment of full-length reads was challenging. To address this, a partial-length alignment strategy was employed, where partial segments were independently aligned to the scaffold reference. The workflow consisted of the following steps:

(1) Scaffold reference construction

Base-wise majority voting was performed on the Type-I reads to generate a consensus sequence, which served as the scaffold reference. If no base was available at a given position, or if the voting result was non-unique, the base at that position was marked with “X”.

(2) Partial-length alignment

The correlation-failed reads were divided into *m* equal-length read segments $\{q_i^1, q_i^2, ..., q_i^m\}$, where q_i^j denotes the *j*-th segment of the *i*-th correlation-failed read and $j \in [1, m]$. Each equal-length read segment q_i^j was aligned to the scaffold reference of length *L* using the Edlib library [9]. If the minimum edit distance $ED_{\min}$ between the read segment q_i^j and the

scaffold reference was below a threshold T_1, the i-th correlation-failed read q_i was labeled as a Type-II read (Figure S29).

Based on the starting position L_i^j of segment q_i^j on the scaffold reference, the estimated start position L_i of the read q_i was calculated as $L_i = L_i^j - (i-1)\times(l/m)$, where l denotes the length of the correlation-failed read.

(3) Simulation performance and analysis

We evaluated the effectiveness of the partial-length alignment approach using simulated data from DNA-40.32kb-ER (R = 1/2). Simulations were conducted at raw sequencing error rates of 0.6% and 1.2%. The performance analysis focused on two key aspects: (i) the effectiveness of partial-length alignment in rescuing correlation-failed reads, (ii) the downstream decoding benefits gained from incorporating these rescued Type-II reads.

(i) Rescue performance of partial-length alignment:

Partial-length alignment enhanced the identification of reads that failed sliding correlation. Compared to full-length alignment, the proposed method recovered 24.79% more reads at an error rate of 0.6% (Figure S31A,B). This improvement further increased to 25.3% at a higher error rate of 1.2% (Figure S32A,B), highlighting the robustness of the approach in handling indel-rich scenarios. Notably, many of the additional reads rescued by partial-length alignment exhibited a characteristic pattern where indel errors were concentrated on only one side of the read. This made them unsuitable for full-length alignment, but they could be successfully localized when aligned segment by segment.

(ii) Decoding benefit from rescued Type-II reads:

Incorporating rescued Type-II reads lowered the required coverage for achieving error-free data recovery. The decoding improvements stemmed from two aspects. For low code rates, the main benefit arose from a reduction in the erasure rate prior to decoding (Figures S33 and S34). For high code rates (*e.g.*, R = 5/6 and R = 0.93), Type-II reads not only helped reduce erasures but also improved the accuracy of the consensus, as reflected by a lower substitution error rate before decoding. As coverage increased, the scaffold reference became more accurate, with both substitution and erasure rates decreasing (Figures S27 and S28). Consequently, more correlation-failed reads were correctly identified as Type-II reads (Figures S31C and S32C), enhancing decoding performance.

Supplementary Note 7: Gap filling through bit-level alignment with regenerative reference

A regenerative reference alignment strategy was employed to identify reads from low-coverage regions and to fill codeword gaps. The workflow followed several key stages:

(1) Regenerative reference construction

The codeword sequences from failed decoding were subjected to a 4-to-5-bit sparsification process following the "sparsification table 1". The sparsified codeword was superimposed with the equal-length watermark sequence. This process generated a two-layer regenerative reference $\underline{F} = \{\underline{F}_1, \underline{F}_2\}$, where $\underline{F}_1$ and $\underline{F}_2$ denote the upper-layer sequence and the lower-layer sequence, respectively.

(2) Bit-level alignment with regenerative reference

The reads that failed to align with the scaffold reference were demapped into two-layer bit sequences. The upper-layer bit sequence $U = \{u^1, u^2, \cdots, u^c\}$ and the lower-layer bit sequence $W = \{w^1, w^2, \cdots, w^c\}$ were obtained, where c denotes the number of unaligned reads. For each unaligned read indexed by $i \in [1, c]$, the upper-layer bit sequence u^i and the lower-layer bit sequence w^i were aligned at the bit level to the upper-layer regenerative reference $\underline{F}_1$ and the lower-layer regenerative reference $\underline{F}_2$, respectively. If the minimum edit distance between u^i and $\underline{F}_1$ was less than the threshold T_2, the starting alignment position P^i_{upper} of u^i relative to $\underline{F}_1$ was recorded. Similarly, the starting alignment position P^i_{below} of w^i relative to $\underline{F}_2$ was determined. The alignment offset was defined as $P^i_{\text{diff}} = |P^i_{\text{upper}} - P^i_{\text{below}}|$. If $P^i_{\text{diff}} < T_3$, the i-th read was labeled as a Type-III read. Its approximate alignment position relative to the regenerative reference was estimated as $P^i_{\text{start}} = (P^i_{\text{upper}} + P^i_{\text{below}})/2$.

Supplementary Note 8: Reproducibility using a pre-built Docker image

To ensure full computational reproducibility, a Docker image and the exact command scripts used in the recovery experiments are publicly available via Zenodo at https://doi.org/10.5281/zenodo.17545118. We provide an overview of the repository and describe how to run the recovery experiments using Docker, as outlined below.

(1) Repository structure

The repository contains the following directories:

Sequencing_data/ – Stores sequencing reads used in the experiments.

Recovery_code/ – Includes the source code used to reproduce the recovery results corresponding to the main and supplementary figures.

Docker_image/ – Provides a pre-built Docker image along with environment setup scripts for consistent and reproducible execution.

(2) Environment setup

All experiments were executed in a controlled Docker environment to ensure full reproducibility. We provide a pre-built Docker image *bootstrap_readout:v1.0* containing all dependencies and exact versions used in the study.

```
# Step 1: Install Docker (if not already installed)
sudo apt-get install -y \
   docker-ce \
   docker-ce-cli \
   containerd.io \
   docker-buildx-plugin \
   docker-compose-plugin
# Step 2: Load local image archive (.tar)
cd Docker_image/
docker load -i bootstrap_readout_v1.0.tar
# Step 3: Set the working directory (local path on your host machine)
parent_dir="$(dirname "$(pwd)")"
work_dir="$parent_dir"
# Step 4: Run the Docker container
docker run -it \
   -v $work_dir:/workspace \
   -w /workspace \
   bootstrap_readout:v1.0 \
   /bin/bash
```

(3) Running recovery experiments

Each dataset is organized in a dedicated folder within *Recovery_code/*, containing the exact commands used to reproduce its corresponding results. For example, the fast recovery experiment on *DNA-40.5kb-DR-SE150.fastq* can be reproduced as follows:

```
# Step 1: Navigate to the experiment directory
cd ./Recovery_code/Figure3/R0.25/
# Step 2: Compile the source program
./build.sh
# Step 3: Execute the complete recovery pipeline
./recover.sh
```

The program performs sliding correlation on *DNA-40.5kb-DR-SE150.fastq*, generating *correlation_result.txt* with four columns: sequence, correlation value, position, and strand. Bit-wise majority voting produces *soft_info.txt*, which records the probability of "0" and "1" at each bit position. Soft-decision LDPC decoding then reconstructs the recovered bitstream named *recovery_bitstream.txt* and restores the original data as *recovery_poem.txt*.

REFERENCES

1. Ezpeleta, Joaquín, Flavia J. Krsticevic, Pilar Bulacio, Elizabeth Tapia. 2017. "Designing robust watermark barcodes for multiplex long-read sequencing." *Bioinformatics* 33: 807-813. https://doi.org/10.1093/bioinformatics/btw322
2. Kracht, David, Steffen Schober. 2015. "Insertion and deletion correcting DNA barcodes based on watermarks." *BMC Bioinformatics* 16: 50. https://doi.org/10.1186/s12859-015-0482-7
3. Davey, Matthew C., and David J. C. MacKay. 1998. "Low density parity check codes over GF (q)." *IEEE Communications Letters* 2: 165-167. https://doi.org/10.1109/4234.686413
4. Achari, Shamin, Ling Cheng. 2023. "Watermark-based code construction for finite-state Markov channel with synchronisation errors." *Digital Signal Processing* 137: 104023. https://doi.org/10.1016/j.dsp.2023.104023
5. Chen, Weigang, Charly Poulliat, David Declercq, Laura Conde-Canencia, Ali Al-Ghouwayel, and Emmanuel Boutillon. 2009. "Non-binary LDPC codes defined over the general linear group: finite length design and practical implementation issues." In *VTC Spring 2009 - IEEE 69th Vehicular Technology Conference*, 1-5. Barcelona, Spain: IEEE. https://doi.org/10.1109/VETECS.2009.5073713

6. Chen, Weigang, Mingzhe Han, Jianting Zhou, Qi Ge, Panpan Wang, Xinchen Zhang, Siyu Zhu, Lifu Song, Yingjin Yuan. 2021. “An artificial chromosome for data storage.” *National Science Review* 8: nwab028. https://doi.org/10.1093/nsr/nwab028
7. Huang, Weichun, Leping Li, Jason R. Myers, Gabor T. Marth. 2012. “ART: a next-generation sequencing read simulator.” *Bioinformatics* 28: 593-594. https://doi.org/10.1093/bioinformatics/btr708
8. Zerbino, Daniel R, Ewan Birney. 2008. “Velvet: algorithms for de novo short read assembly using de Bruijn graphs.” *Genome Research* 18: 821-829. https://doi.org/10.1101/gr.074492.107
9. Šošić, Martin, Mile Šikić. 2017. “Edlib: a C/C++ library for fast, exact sequence alignment using edit distance.” *Bioinformatics* 33: 1394-1395. https://doi.org/10.1093/bioinformatics/btw753
10. Li, Ruiqiang, Hongmei Zhu, Jue Ruan, Wubin Qian, Xiaodong Fang, Zhongbin Shi, Yingrui Li, et al. 2010. “De novo assembly of human genomes with massively parallel short read sequencing.” *Genome Research* 20: 265-272. https://doi.org/10.1101/gr.097261.109
11. Simpson, Jared T, Kim Wong, Shaun D Jackman, Jacqueline E Schein, Steven J M Jones, Inanc Birol. 2009. “ABySS: a parallel assembler for short read sequence data.” *Genome Research* 19: 1117-1123. https://doi.org/10.1101/gr.089532.108
12. Yazdi, S. M. Hossein Tabatabaei, Ryan Gabrys, Olgica Milenkovic. 2017. “Portable and error-free DNA-based data storage.” *Scientific Reports* 7: 5011. https://doi.org/10.1038/s41598-017-05188-1
13. Lopez, Randolph, Yuan-Jyue Chen, Siena Dumas Ang, Sergey Yekhanin, Konstantin Makarychev, Miklos Z. Racz, Georg Seelig, Karin Strauss, Luis Ceze. 2019. “DNA assembly for nanopore data storage readout.” *Nature Communications* 10: 2933. https://doi.org/10.1038/s41467-019-10978-4
14. Zhang, Qiao, Kai Xia, Meng Jiang, Qingting Li, Weigang Chen, Mingzhe Han, Wei Li, et al. 2023. “Catalytic DNA-assisted mass production of arbitrary single-stranded DNA.” *Angewandte Chemie International Edition* 62: e202212011. https://doi.org/10.1002/anie.202212011
15. Sun, Fajia, Yiming Dong, Ming Ni, Zhi Ping, Yuhui Sun, Qi Ouyang, Long Qian. 2023. “Mobile and self-sustained data storage in an extremophile genomic DNA.” *Advanced Science* 10: 2206201. https://doi.org/10.1002/advs.202206201
16. Ge, Qi, Rui Qin, Shuang Liu, Quan Guo, Changcai Han, Weigang Chen. 2025. “Pragmatic soft-decision data readout of encoded large DNA.” *Briefings in Bioinformatics* 26:

bbaf102. https://doi.org/10.1093/bib/bbaf102

17. Ping, Zhi, Shihong Chen, Guangyu Zhou, Xiaoluo Huang, Sha Joe Zhu, Haoling Zhang, Henry H. Lee, et al. 2022. "Towards practical and robust DNA-based data archiving using the yin–yang codec system." *Nature Computational Science* 2: 234-242. https://doi.org/10.1038/s43588-022-00231-2
18. Chen, Weigang, Qi Ge, Panpan Wang, Mingzhe Han, and Jian Guo. 2021. "Multiple interleaved RS codes for data storage using up to Mb-scale synthetic DNA in living cells." *Synthetic Biology Journal* 2: 428-443. https://doi.org/10.12211/2096-8280.2021-023

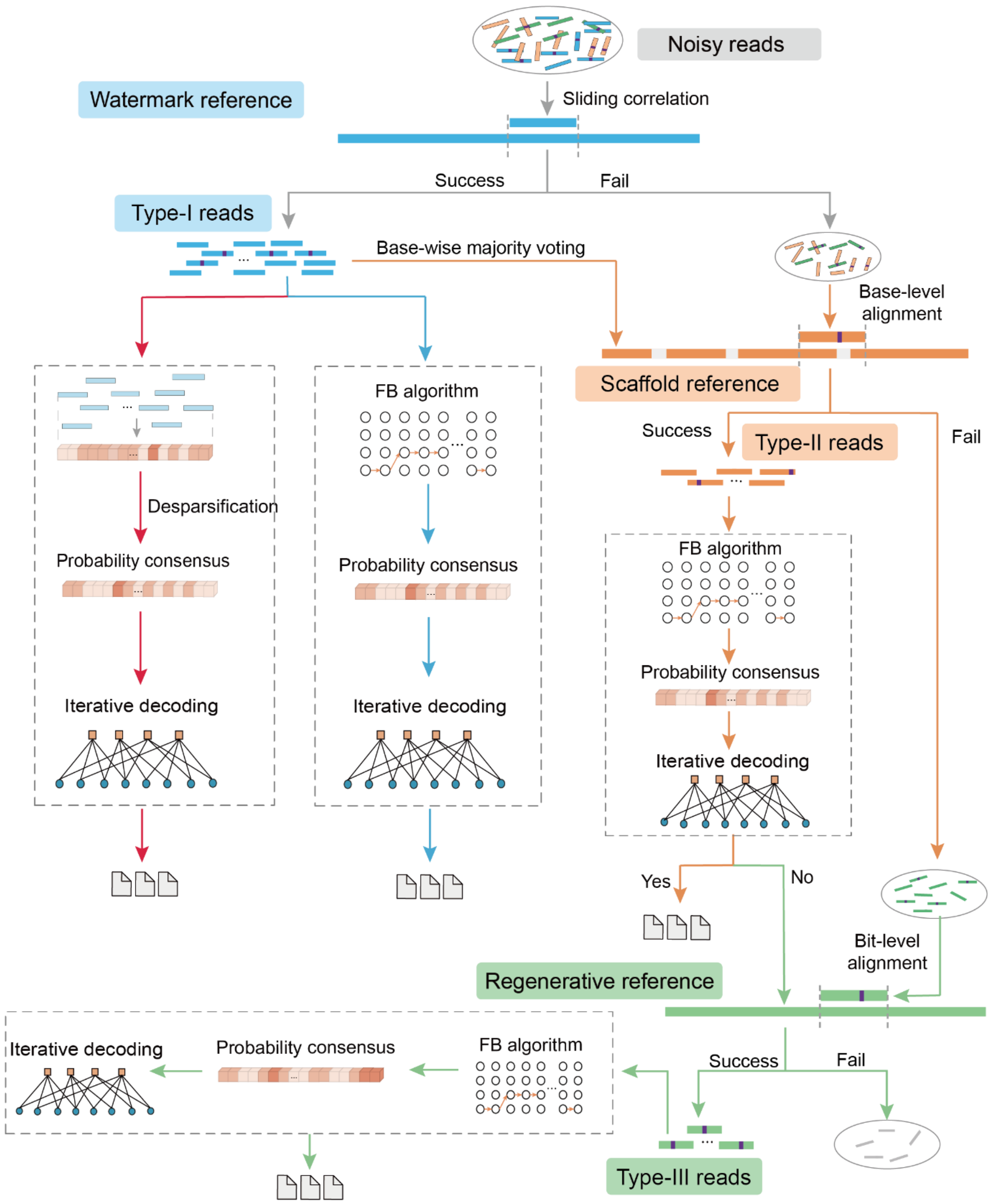

Figure S1 Workflow of readout in a bootstrap manner for large DNA fragments using multiple-fold hidden references. The red path represents the fast readout scheme, where low-error reads are identified via correlation to the watermark reference. These Type-I reads are directly used for bit-wise consensus for soft-decision decoding. In the presence of indel errors, scaffold and regenerative references are subsequently constructed to identify Type-II and Type-III reads (orange and green paths, respectively), thereby maximizing read utilization and ensuring robust decoding under varying error conditions.

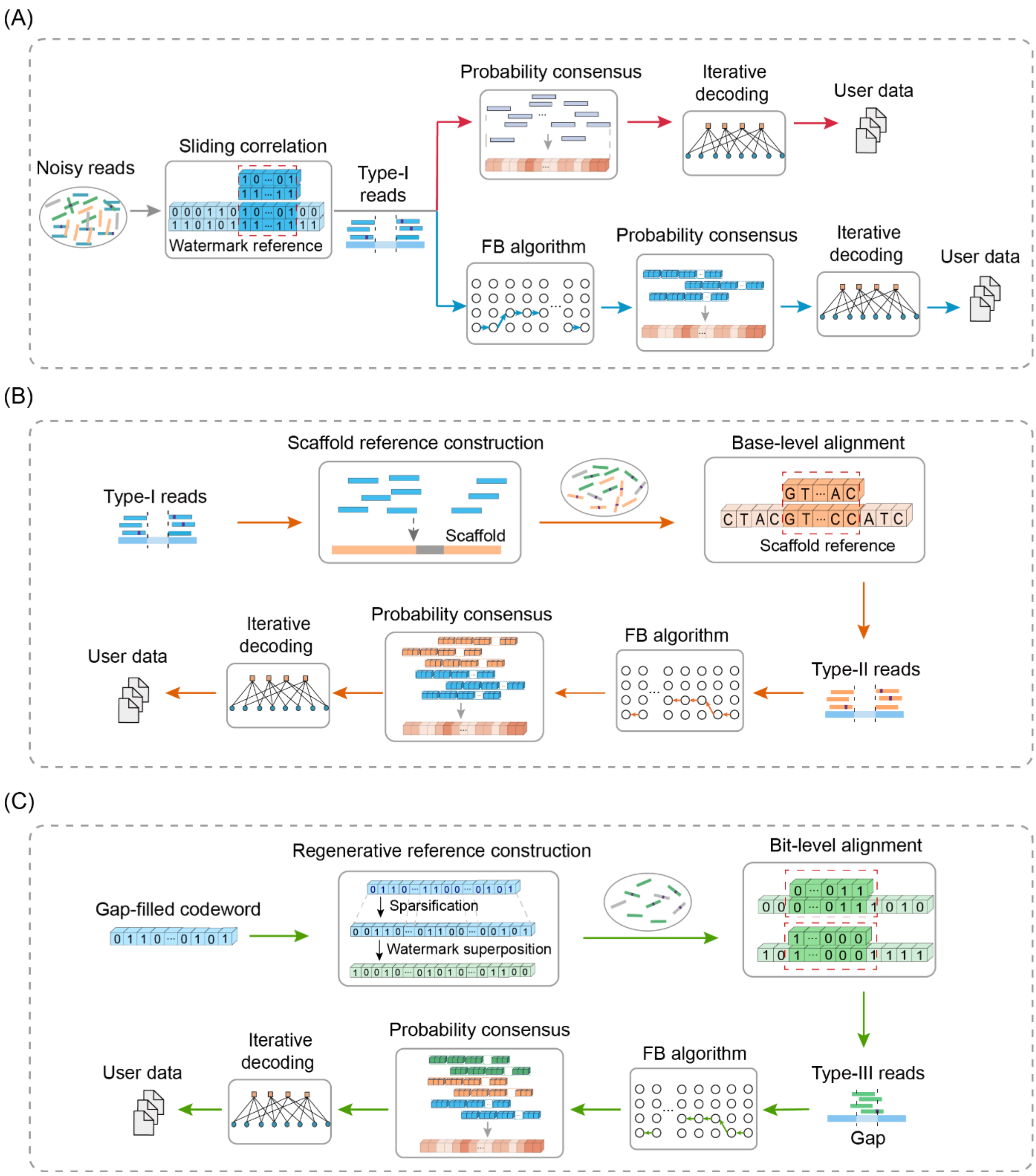

Figure S2 Data recovery framework in a bootstrap manner using multiple-fold hidden references. The proposed approach systematically searches all reads in stages, guided by three types of references. (A) Watermark references enable rapid identification of low-error reads for early decoding. (B) The scaffold reference, combined with the FBA, enables recovery of reads affected by indel errors. (C) The regenerative reference fills low-coverage regions, maximizing read utilization and ensuring reliable recovery.

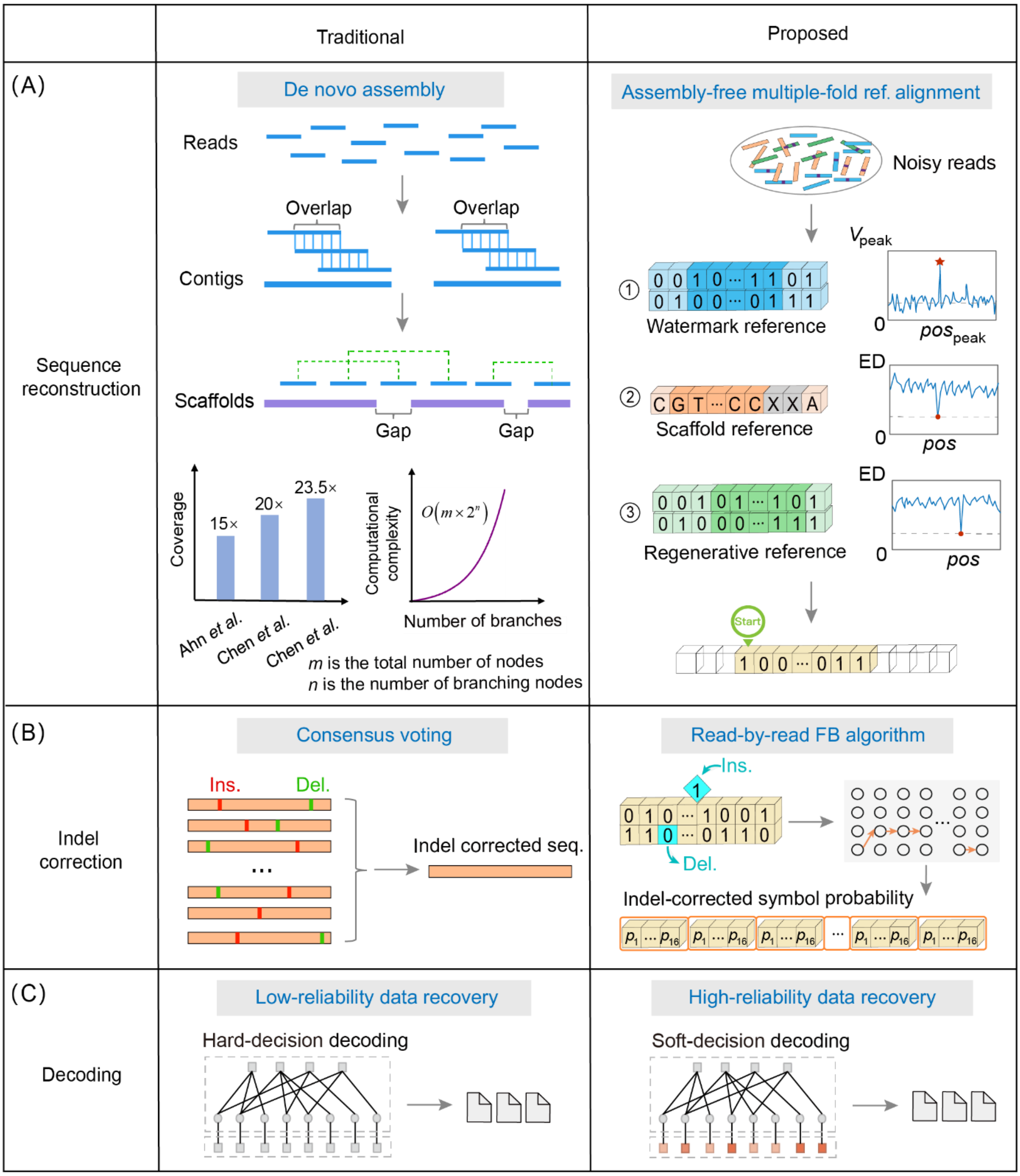

Figure S3 Comparison of the proposed assembly-free readout with traditional assembly-based methods. Traditional pipelines rely on *de novo* assembly of short reads into contigs and scaffolds, which requires high coverage and leaves gaps [8,10,11]. In contrast, our method uses multiple-fold hidden references to directly anchor reads, transforming the process into a resequencing-like workflow. For indel correction, conventional approaches depend on high-coverage consensus [12,13], whereas our method applies a read-by-read FBA for more efficient correction. Additionally, soft-decision decoding is employed instead of hard-decision decoding, improving robustness under noisy conditions.

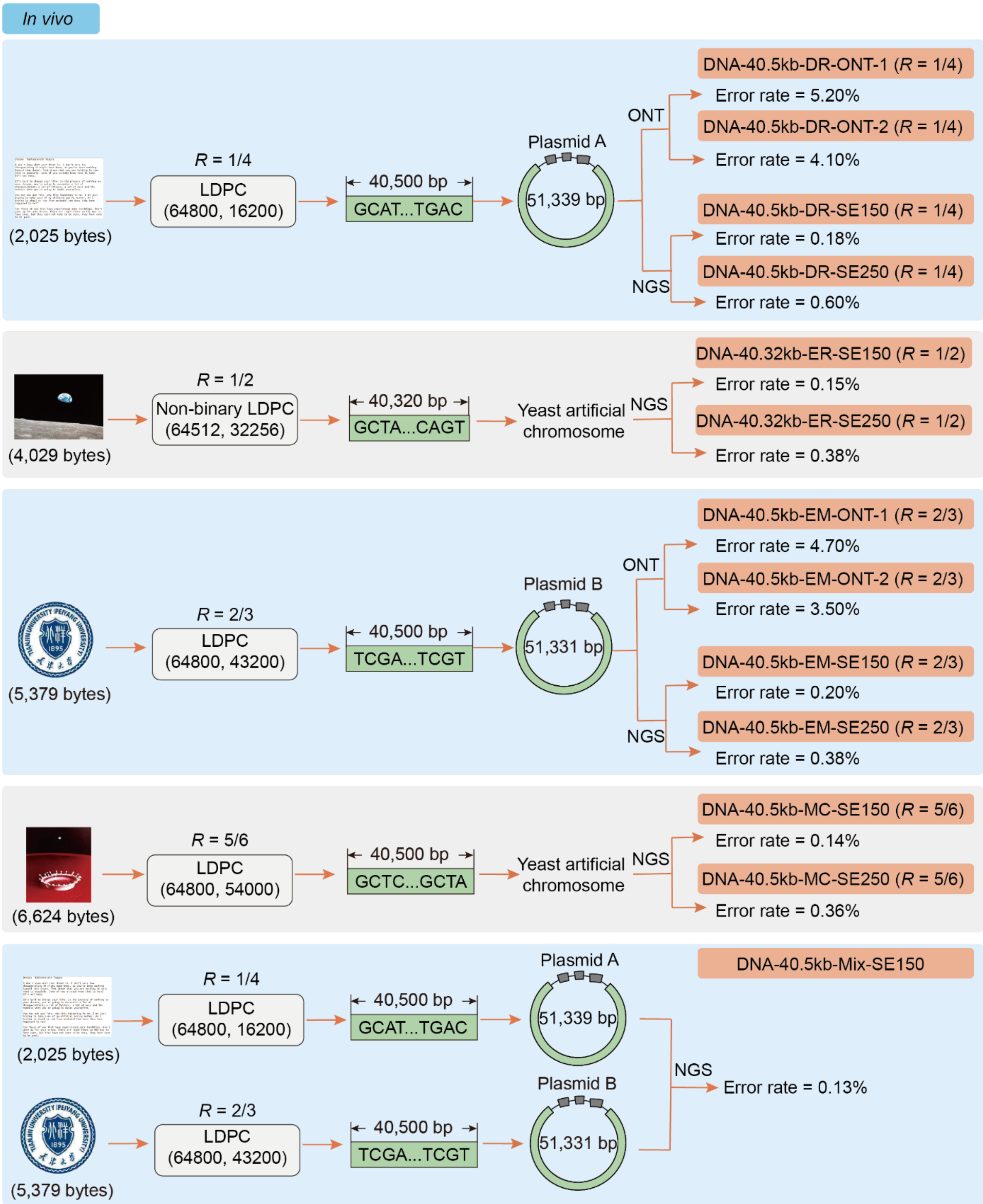

Figure S4 Experimental verification of the proposed framework. Four large DNA fragments with code rates of 1/4, 1/2, 2/3, and 5/6 were synthesized and sequenced using Illumina for *in vivo* validation. Two independent ONT sequencing runs on large DNA fragments (with code rates of 1/4 and 2/3) yielded higher raw error rates than Illumina sequencing. Where DR, EM, ER, and MC are abbreviations of the source data *Dream.txt*, *Emblem.jpg*, *Earth rise.jpg*, and *Milk coronet.jpg*, respectively.

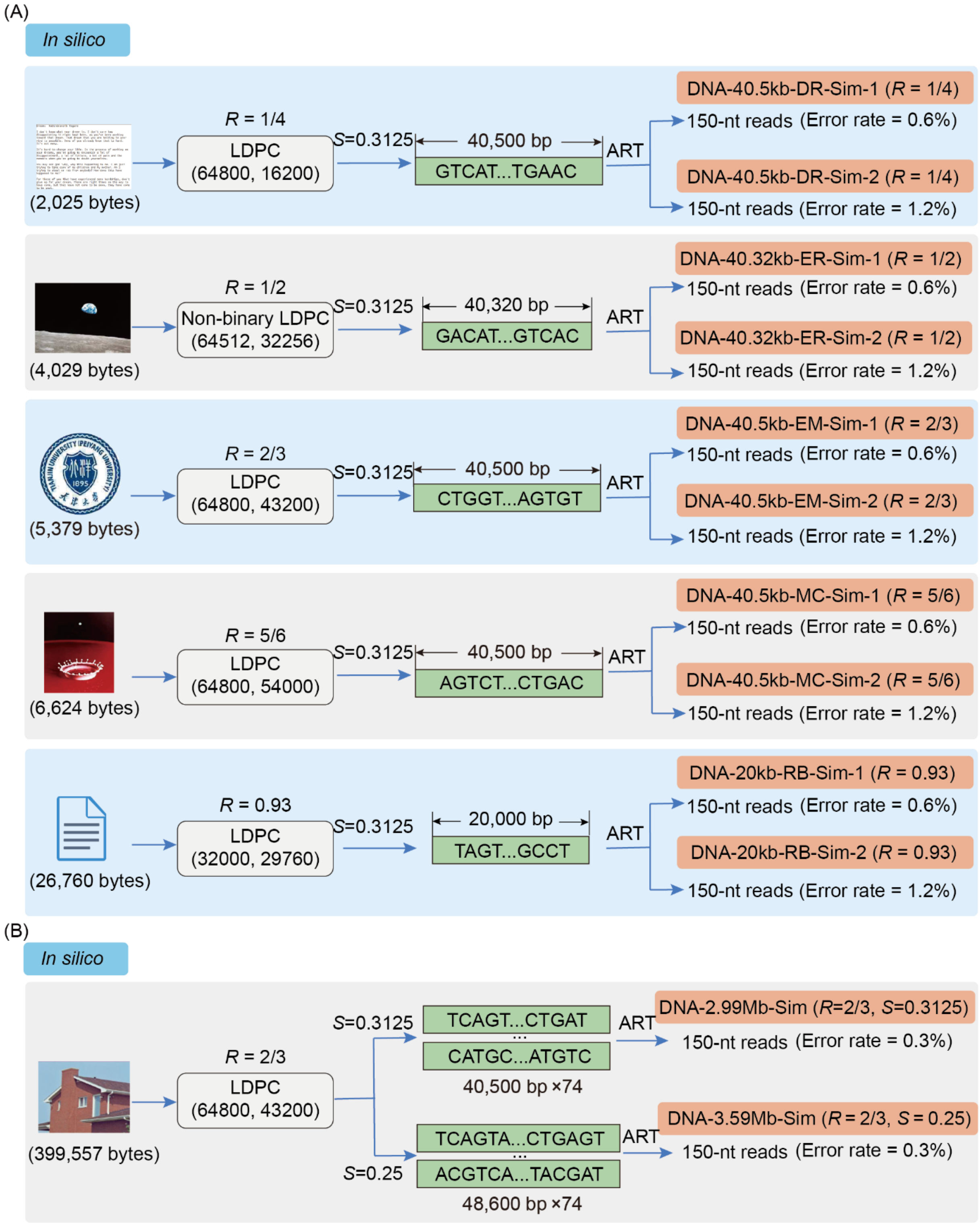

Figure S5 Simulation verification of the proposed framework. (A) *In silico* simulations with code rates of 1/4, 1/2, 2/3, 5/6, and 0.93 assessed the scalability of the proposed method. (B) *In silico* simulations (R = 2/3) with sparsity ratios of 0.3125 and 0.25 evaluated the effect of coding sparsity on correlation accuracy. Where DR, EM, ER, MC, and RB are abbreviations of the source data *Dream.txt*, *Emblem.jpg*, *Earth rise.jpg*, *Milk coronet.jpg*, and *random bits*, respectively.

(A)

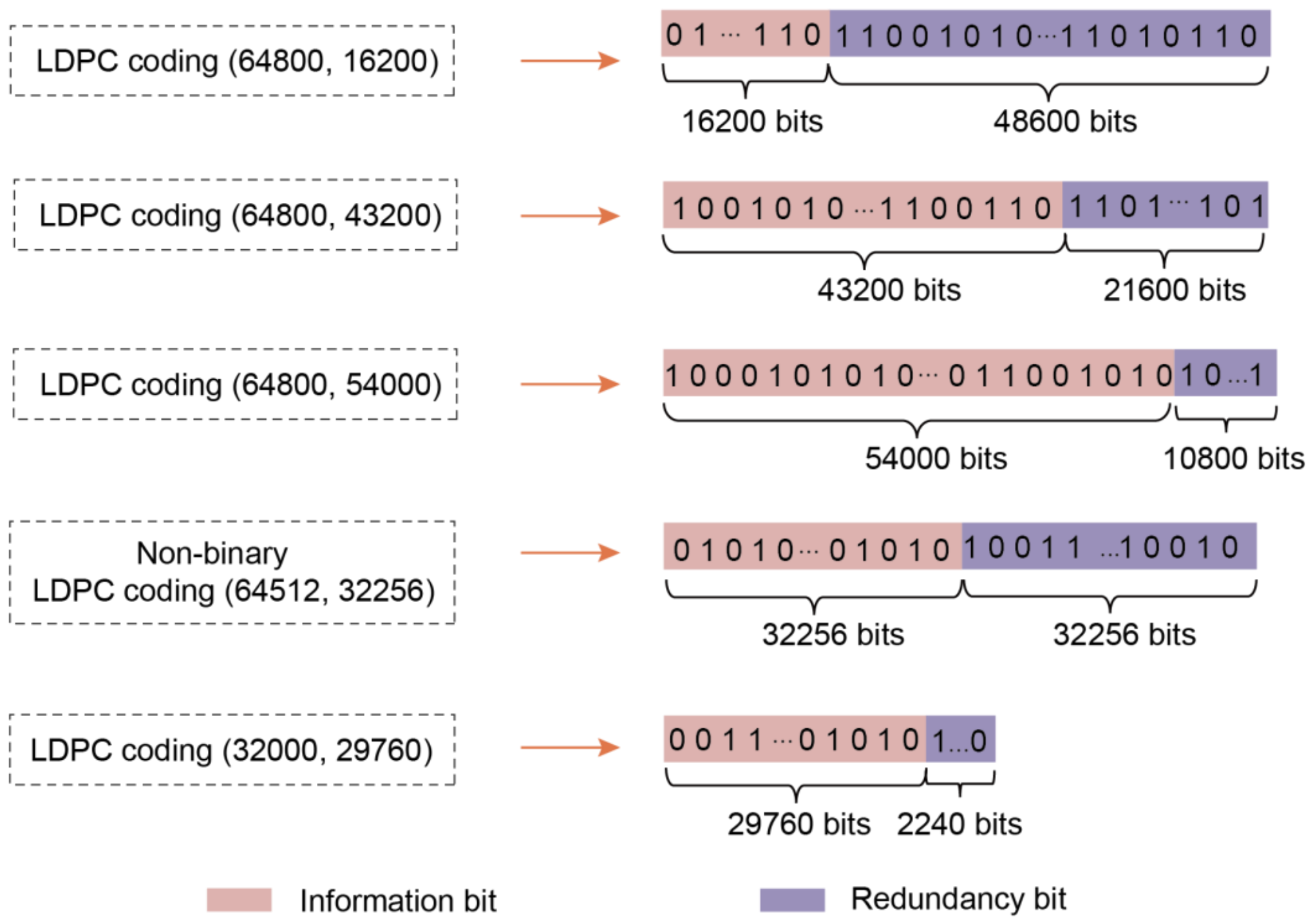

(B)

Sparsification table 1 (*S* = 0.3125)

Before	After	Before	After
0000	00000	1000	01000
0001	00001	1001	01001
0010	00010	1010	01010
0011	00011	1011	10100
0100	00100	1100	01100
0101	00101	1101	10010
0110	00110	1110	10001
0111	11000	1111	10000

Sparsification table 2 (*S* = 0.25)

Before	After	Before	After
0000	000000	1000	001000
0001	000001	1001	101000
0010	000010	1010	100100
0011	010000	1011	100010
0100	000100	1100	010001
0101	100000	1101	010010
0110	001010	1110	100001
0111	010100	1111	001001

Figure S6 Overview of the coding schemes and sparsification rules. (A) Information and redundancy bit distributions for five LDPC coding configurations used in this study. (B) Sparsification rules used in this study, with bit-mapping tables shown for sparsity ratios of 0.3125 and 0.25.

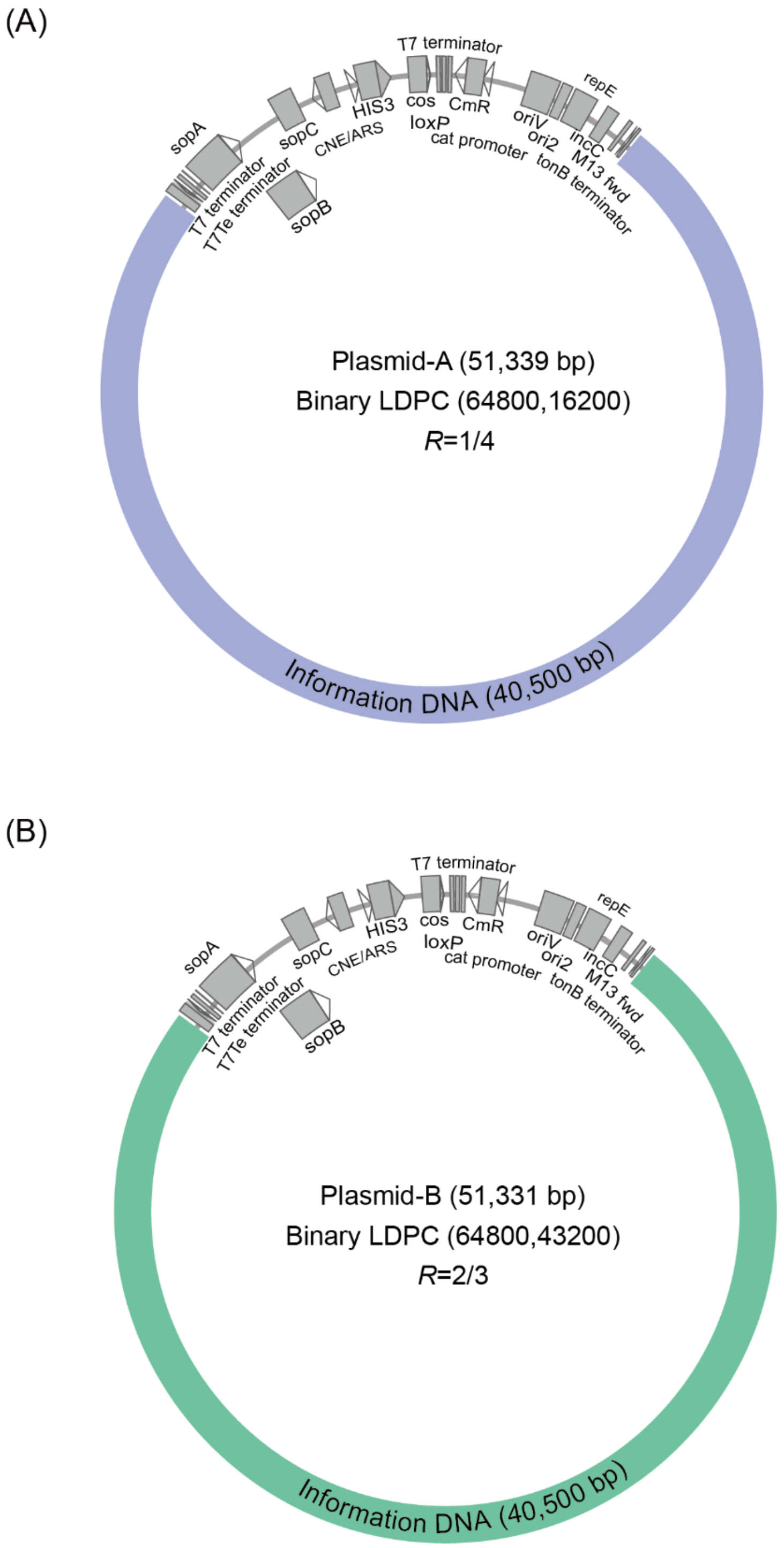

Figure S7 Schematic diagrams of the two data-carrying plasmids. DNA fragments containing encoded information were synthesized and cloned into the pCC1413 vector. (A) Plasmid-A is a circular plasmid of 51,339 bp carrying 40,500 bp of information DNA with a code rate of 1/4. (B) Plasmid-B is a circular plasmid of 51,331 bp, also carrying 40,500 bp of information DNA with a code rate of 2/3.

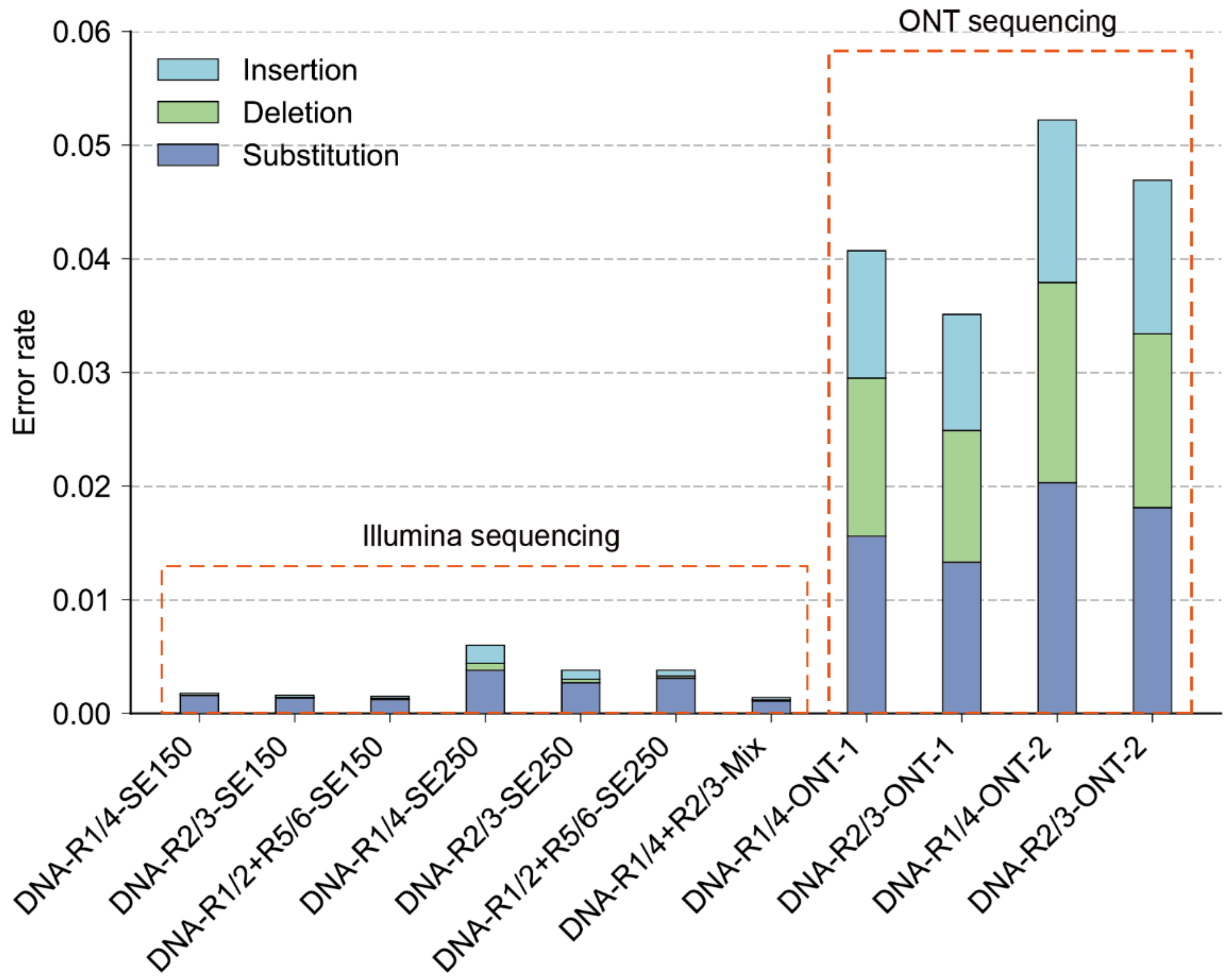

Figure S8 Raw error rates of real sequencing reads. Plasmid-A (DNA-40.5kb-DR, R = 1/4), Plasmid-B (DNA-40.5kb-EM, R = 2/3), and an artificial chromosome carrying DNA-40.32kb-ER (R = 1/2) and DNA-40.5kb-MC (R = 5/6) were synthesized and sequenced using both Illumina and Oxford Nanopore Technology (ONT) sequencers. Illumina sequencing was performed in Single-End (SE) mode with read lengths of 150 bp and 250 bp. The error rate was calculated as the Levenshtein distance between each noisy read and the reference sequence, normalized by the aligned length. Reads with error rates below 0.2 were retained for the distributions shown.

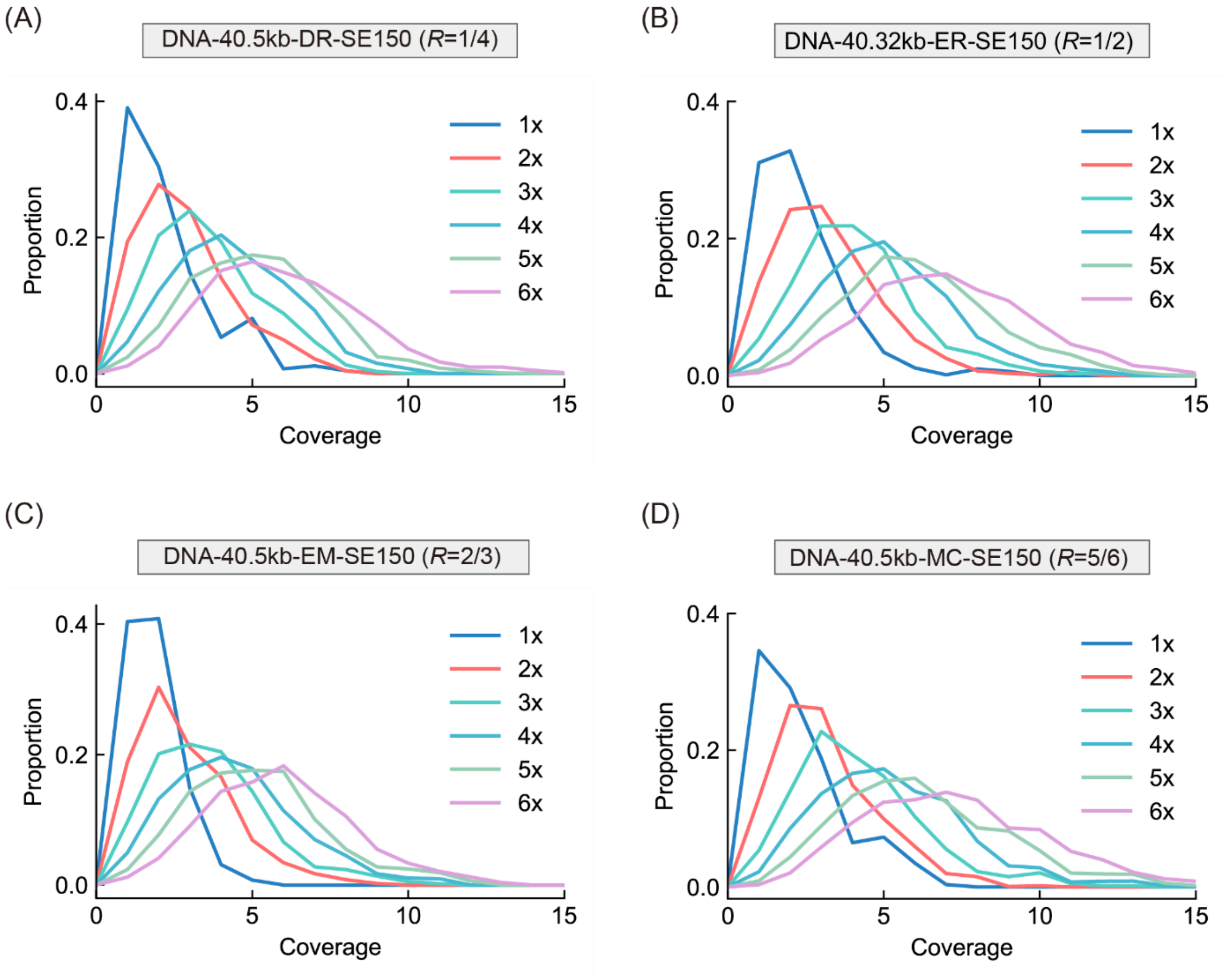

Figure S9 Base-coverage distribution of Illumina reads. Sequencing coverage ranged from 1× to 6×. With increasing average coverage, the base-coverage distribution became broader and smoother. (A) DNA-40.5kb-DR-SE150 (R = 1/4). (B) DNA-40.32kb-ER-SE150 (R = 1/2). (C) DNA-40.5kb-EM-SE150 (R = 2/3). (D) DNA-40.5kb-MC-SE150 (R = 5/6).

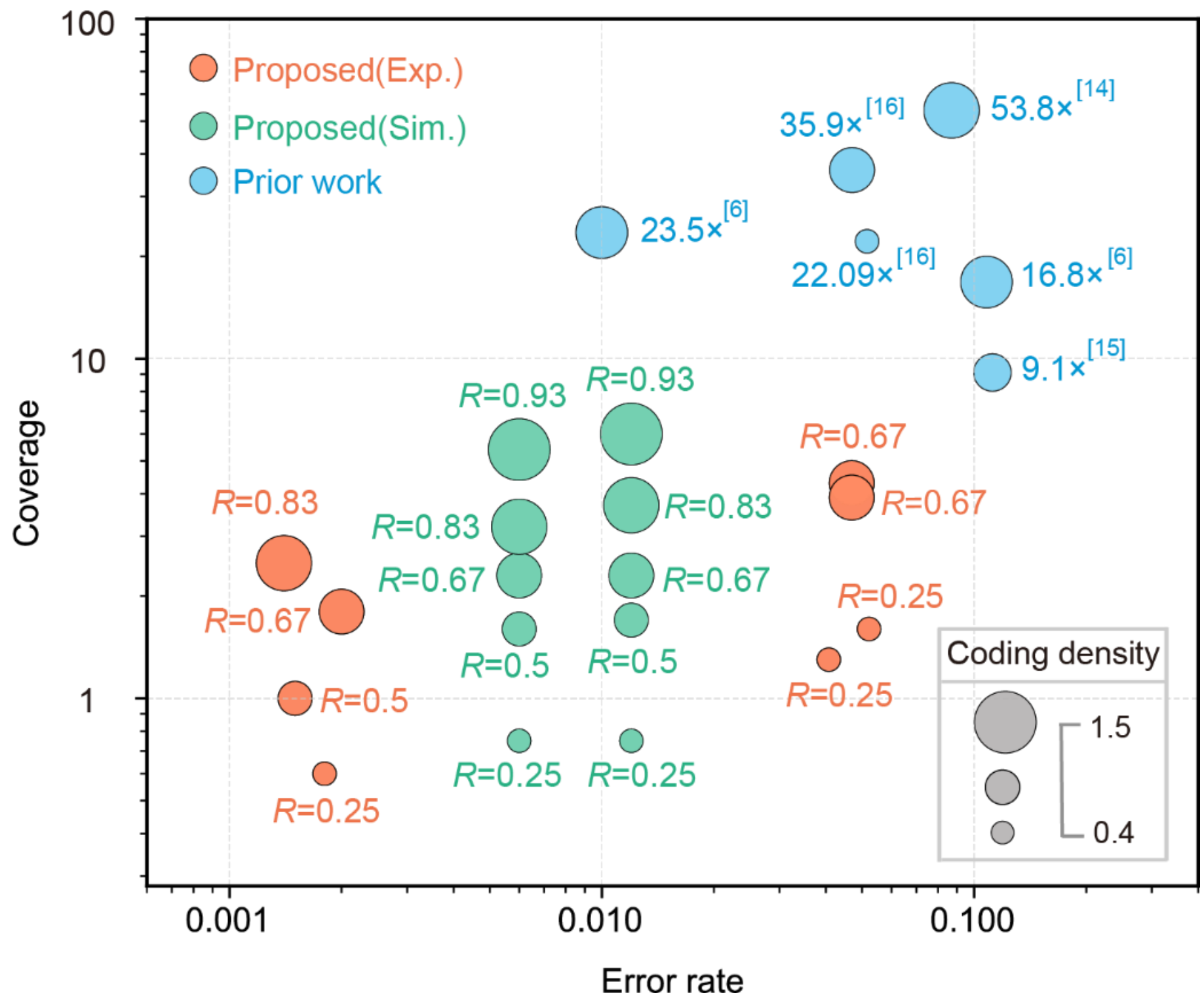

Figure S10 Comparison with other DNA data storage schemes. *In silico* simulations with different code rates (1/4, 1/2, 2/3, 5/6, and 0.93) were conducted to assess the scalability of the proposed method. Blue bubbles represent the simulated recovery performance of our readout framework. The simulations were performed under two error settings: a raw error rate of 0.6% ($P_i : P_d : P_s$ = 10: 5: 2) and 1.2% ($P_i : P_d : P_s$ = 1: 1: 2). The bubble size represents the encoding density, with larger bubbles indicating higher densities.

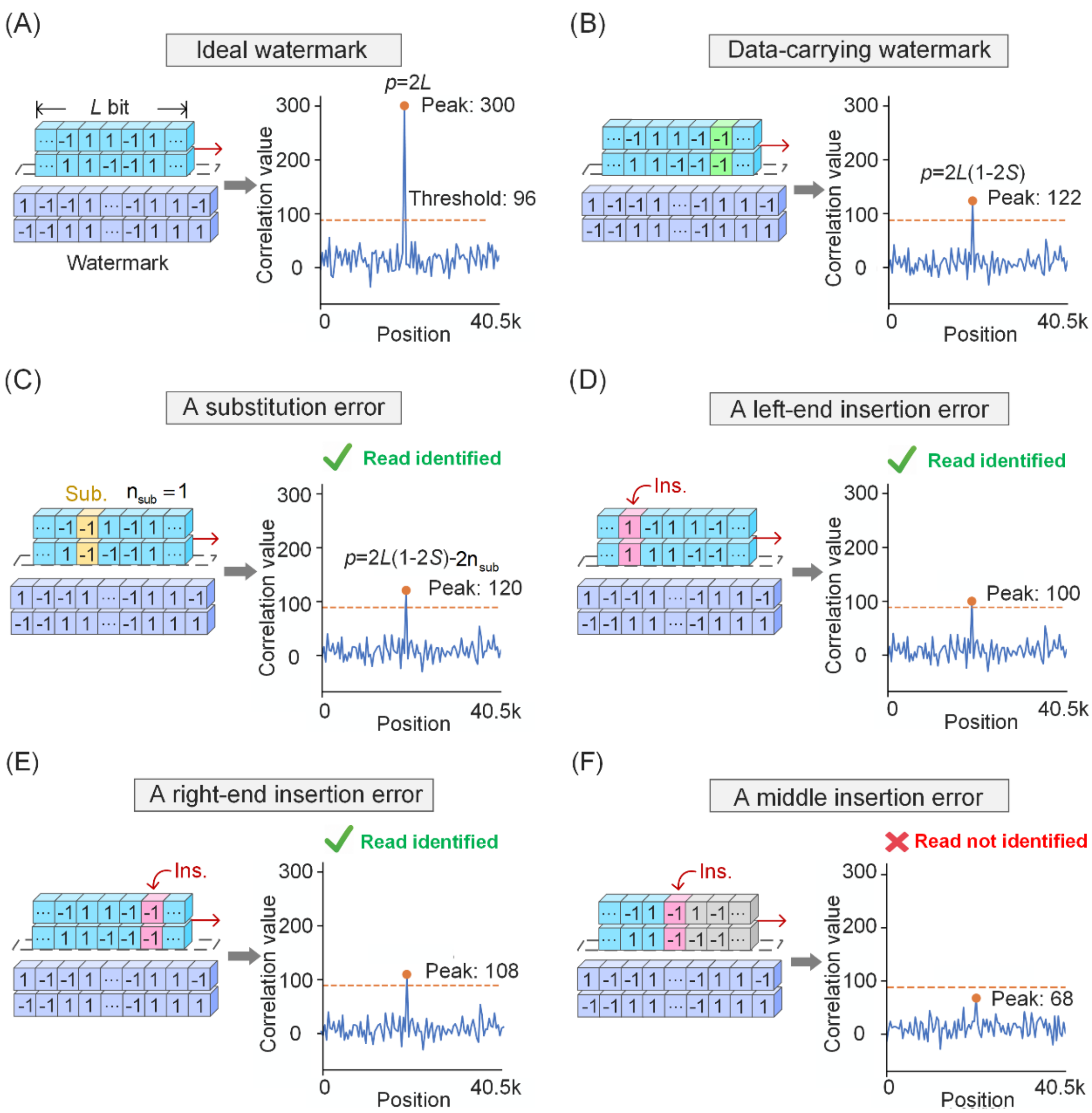

Figure S11 Correlation-based read identification under different types of noise. (A) An ideal watermark without errors, exhibiting the theoretical maximum correlation peak. (B) A data-carrying watermark derived by embedding codewords into the ideal watermark, resulting in a reduced correlation peak. (C) A substitution error introduced at an arbitrary position slightly lowered the peak, but the read remained identifiable. (D) and (E) Insertion errors near the left or right end of the read also reduced the peak but still allowed successful identification above the preset threshold. (F) An insertion error near the center of the read significantly disrupted correlation, reducing the peak below the threshold and resulting in identification failure.

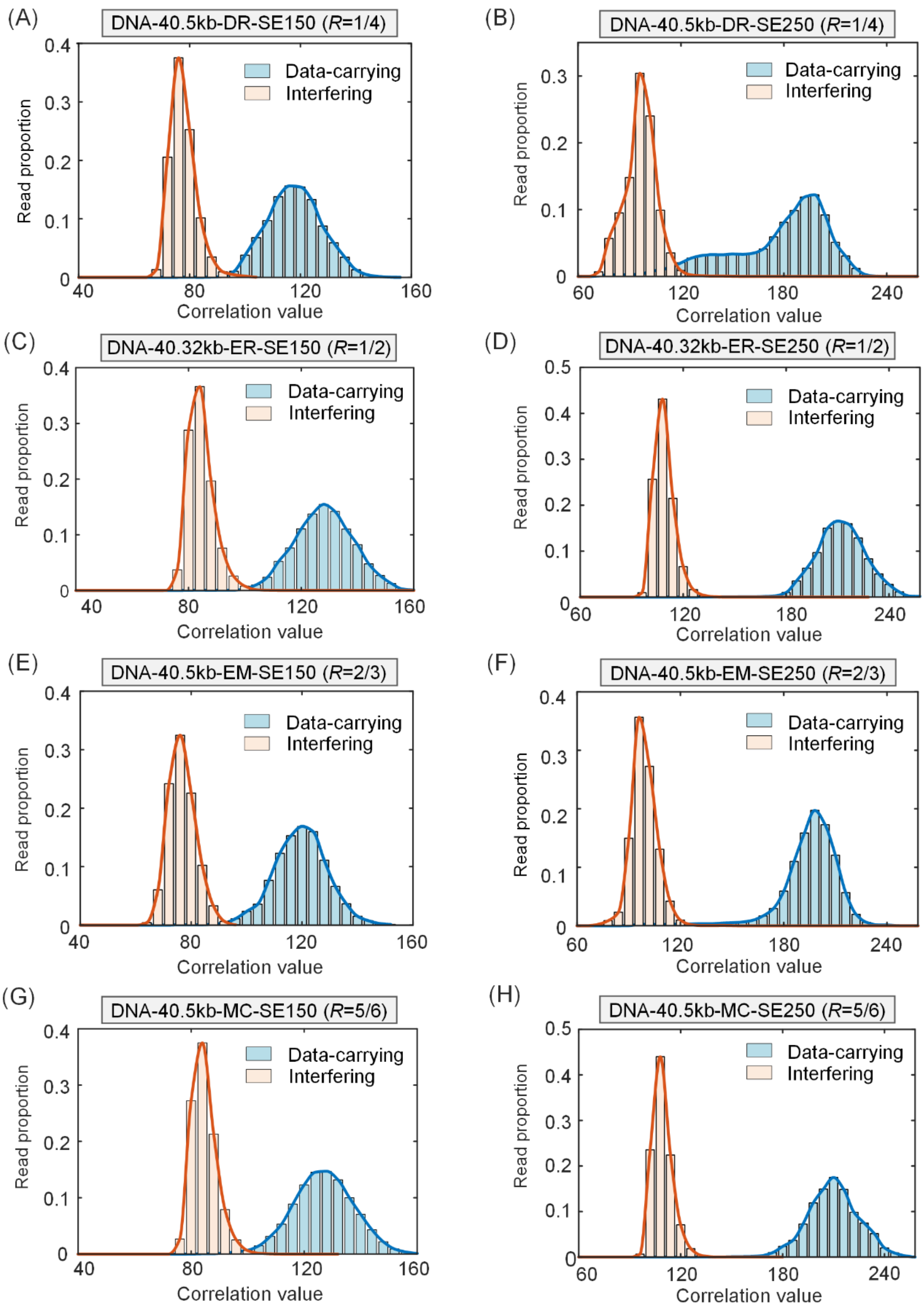

Figure S12 Distribution of correlation peak values for data-carrying and interfering reads. (A) DNA-40.5kb-DR-SE150 (R = 1/4). (B) DNA-40.5kb-DR-SE250 (R = 1/4). (C) DNA-40.32kb-ER-SE150 (R = 1/2). (D) DNA-40.32kb-ER-SE250 (R = 1/2). (E) DNA-40.5kb-EM-SE150 (R = 2/3). (F) DNA-40.5kb-EM-SE250 (R = 2/3). (G) DNA-40.5kb-MC-SE150 (R = 5/6). (H) DNA-40.5kb-MC-SE250 (R = 5/6).

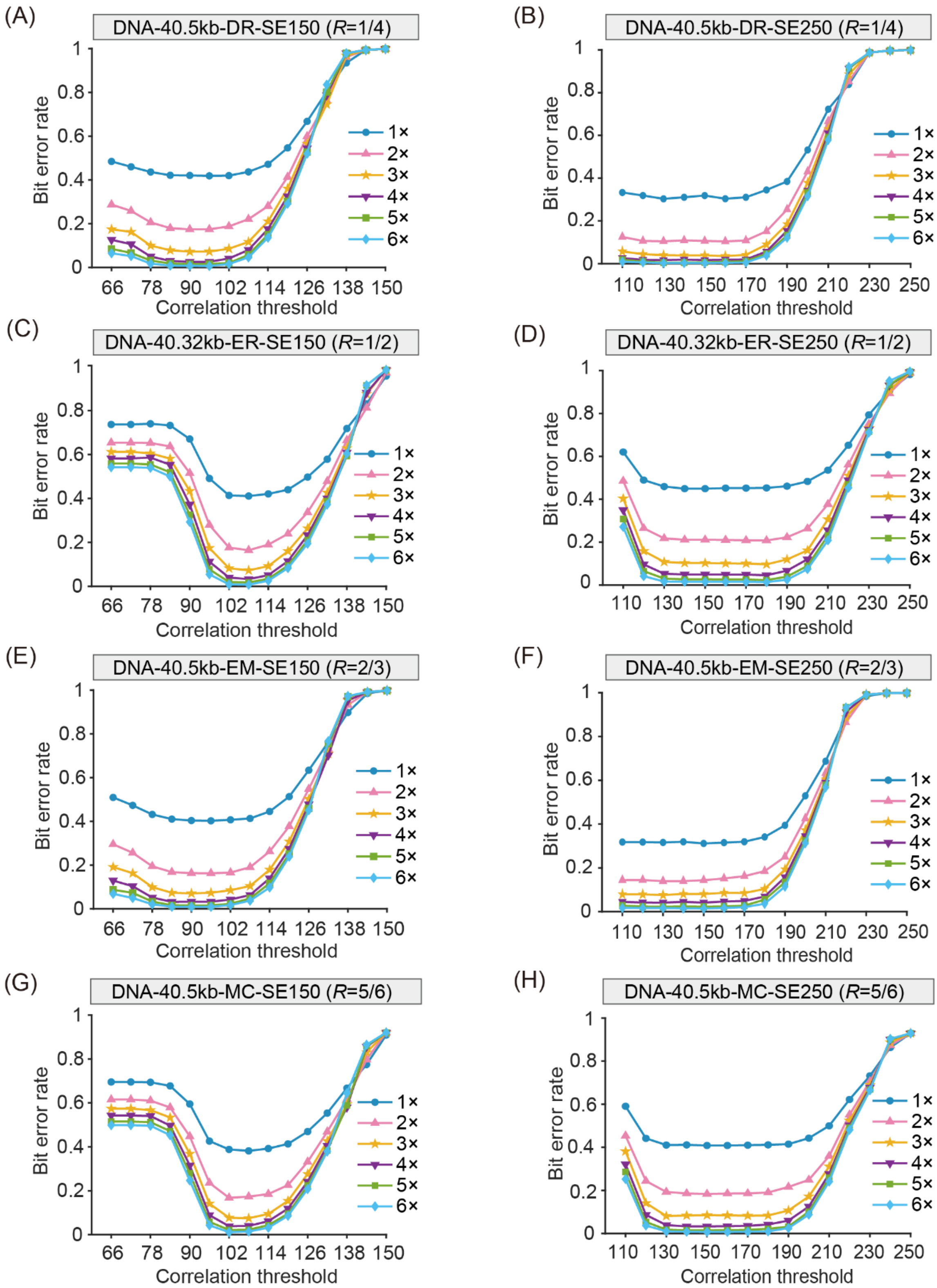

Figure S13 Bit error rate distribution before decoding under different correlation thresholds. (A) DNA-40.5kb-DR-SE150 (*R* = 1/4). (B) DNA-40.5kb-DR-SE250 (*R* = 1/4). (C) DNA-40.32kb-ER-SE150 (*R* = 1/2). (D) DNA-40.32kb-ER-SE250 (*R* = 1/2). (E) DNA-40.5kb-EM-SE150 (*R* = 2/3). (F) DNA-40.5kb-EM-SE250 (*R* = 2/3). (G) DNA-40.5kb-MC-SE150 (*R* = 5/6). (H) DNA-40.5kb-MC-SE250 (*R* = 5/6).

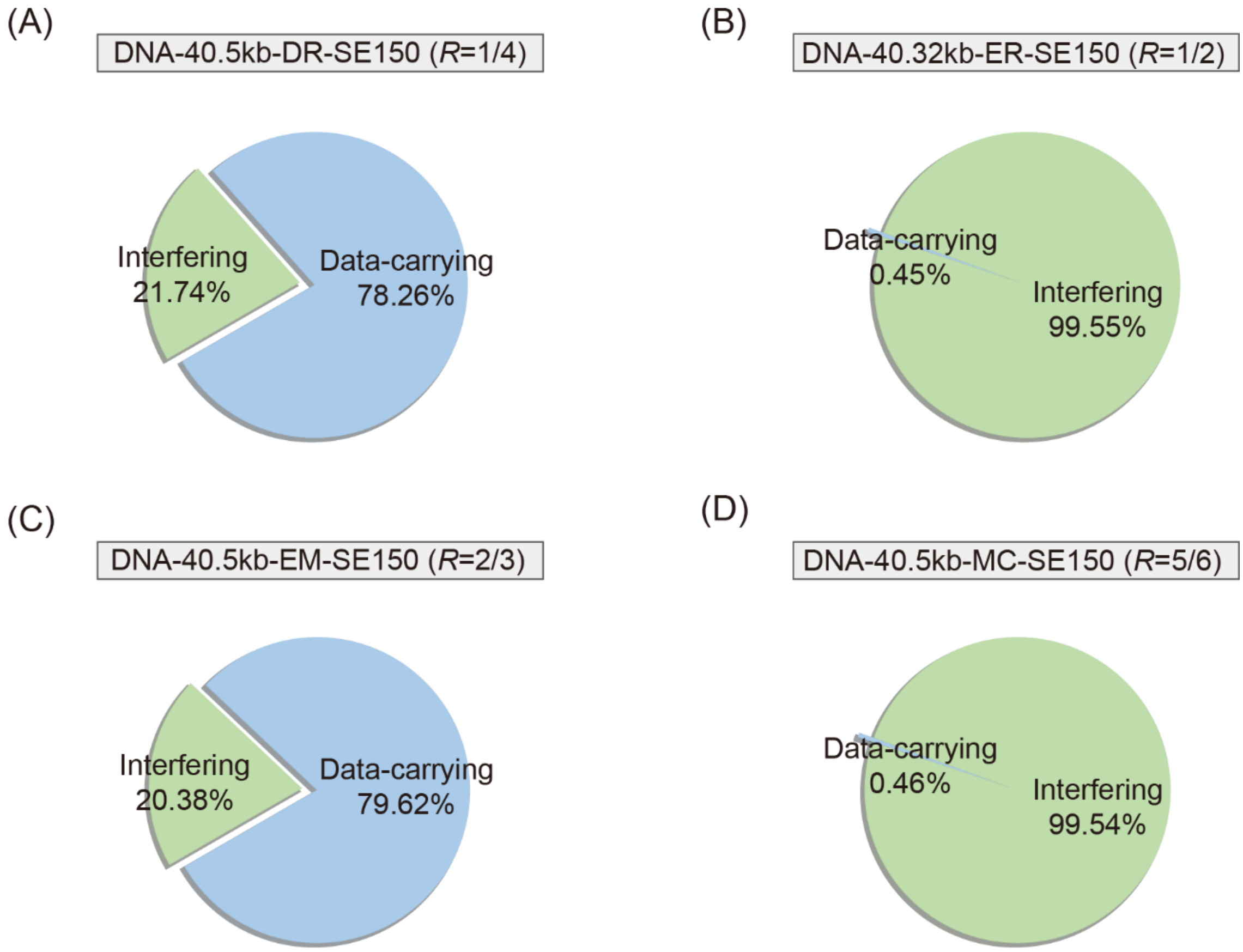

Figure S14 Proportions of data-carrying and interfering reads in sequencing data. Pie charts show the proportions of data-carrying and interfering reads in the sequencing results of four encoded large DNA fragments. Data-carrying reads are defined as those aligning to the corresponding reference sequence, while the remaining reads are considered interfering reads. (A) DNA-40.5kb-DR-SE150 (R = 1/4). (B) DNA-40.32kb-ER-SE150 (R = 1/2). (C) DNA-40.5kb-EM-SE150 (R = 2/3). (D) DNA-40.5kb-MC-SE150 (R = 5/6). (A) and (C) were derived from plasmids. (B) and (D) were derived from a synthetic chromosome assembled in yeast, where interference primarily originates from host genomic sequences.

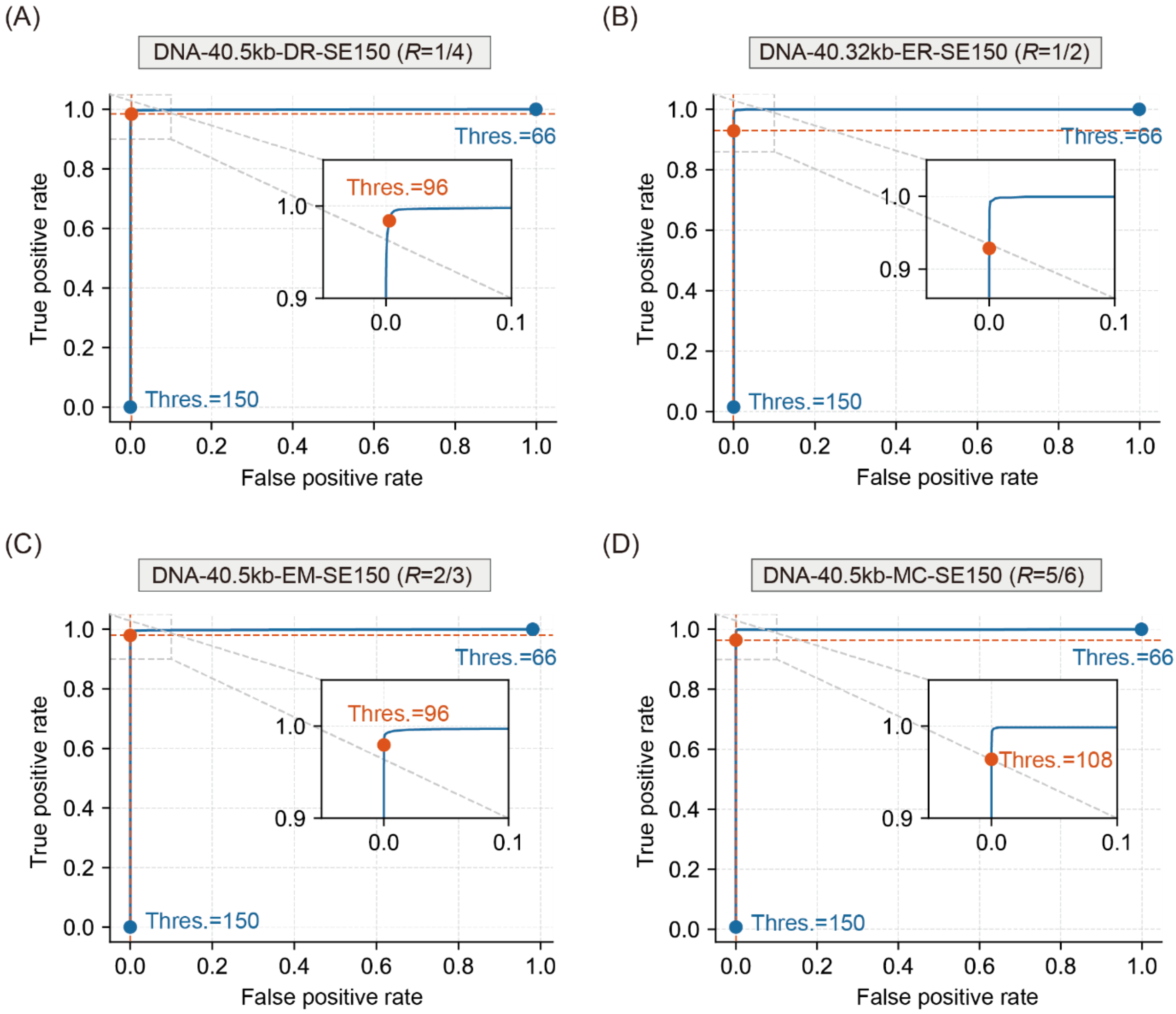

Figure S15 ROC curves of correlation-based filtering under different thresholds across datasets. For each dataset, prediction labels were generated from correlation outputs under varying thresholds (66–150, step = 1), and ground-truth labels were determined from sequence alignments. The four datasets include: (A) DNA-40.5kb-DR-SE150 (R = 1/4), (B) DNA-40.32kb-ER-SE150 (R = 1/2), (C) DNA-40.5kb-EM-SE150 (R = 2/3), and (D) DNA-40.5kb-MC-SE150 (R = 5/6). In each panel, the true positive rate (TPR) is plotted against the false positive rate (FPR). The orange markers indicate the selected operating points. The corresponding coordinates of these selected thresholds are as follows: (A) FPR = 0.0027, TPR = 0.9840; (B) FPR = 0.0002, TPR = 0.9826; (C) FPR = 0.0001, TPR = 0.9795; (D) FPR = 0.0000, TPR = 0.9640.

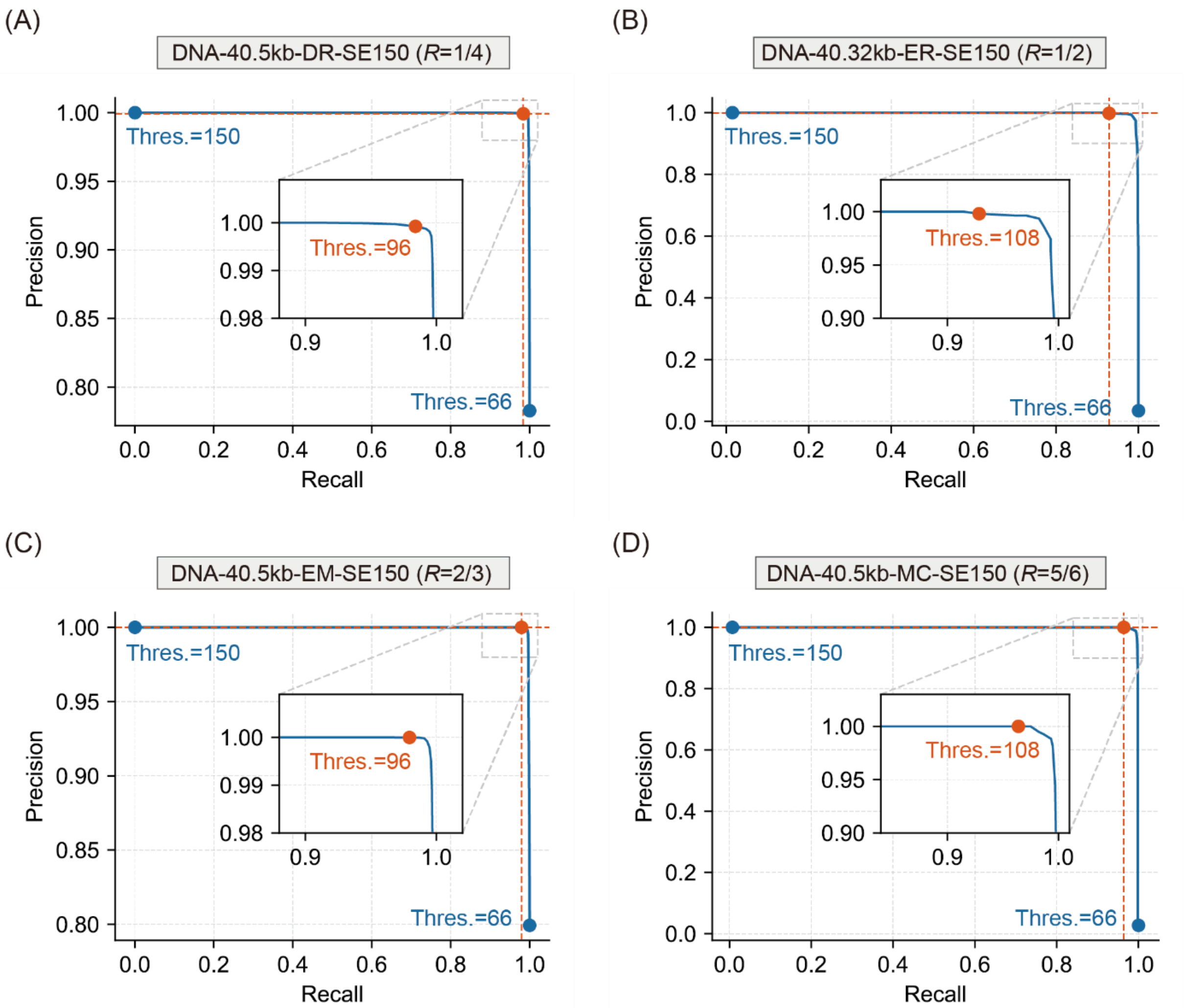

Figure S16 PR curves of correlation-based filtering under different thresholds across datasets. For each dataset, prediction labels were generated from correlation outputs under varying thresholds (66–150, step = 1), and ground-truth labels were determined from sequence alignments. The four datasets include: (A) DNA-40.5kb-DR-SE150 (*R* = 1/4), (B) DNA-40.32kb-ER-SE150 (*R* = 1/2), (C) DNA-40.5kb-EM-SE150 (*R* = 2/3), and (D) DNA-40.5kb-MC-SE150 (*R* = 5/6). The orange markers indicate the selected thresholds. The coordinates of the selected points are: (A) Recall = 0.9841, Precision = 0.9992; (B) Recall = 0.9826, Precision = 0.9935; (C) Recall = 0.9795, Precision = 1.0000; (D) Recall = 0.9640, Precision = 1.0000.

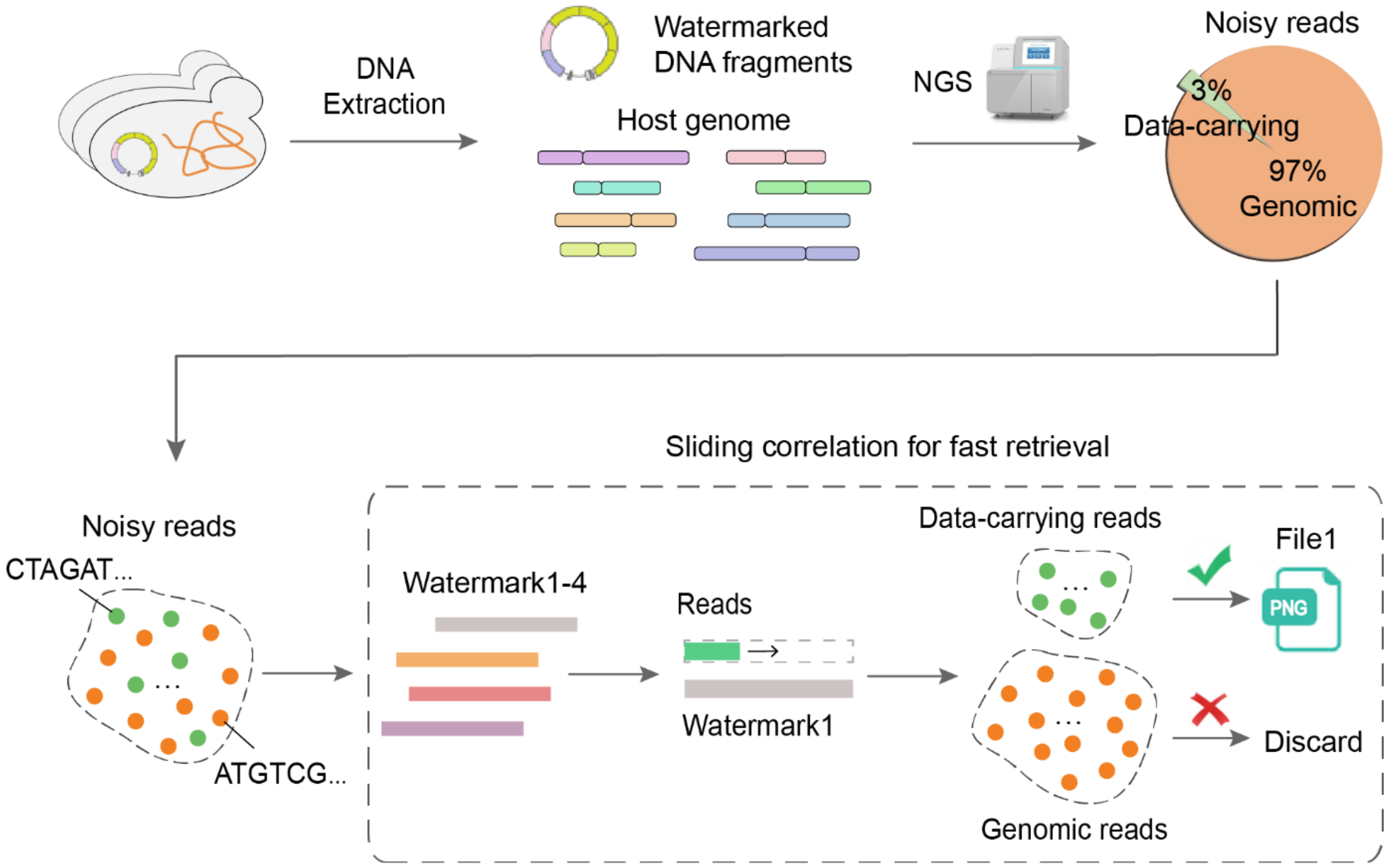

Figure S17 Data recovery under host genome interference. Nucleic acid extraction from yeast cells yields both the watermark-encoded DNA sequences and the host genome. The extracted material was sequenced using next-generation sequencing (NGS), generating a mixed dataset comprising ~97% interfering reads from the host genome and ~3% data-carrying reads.

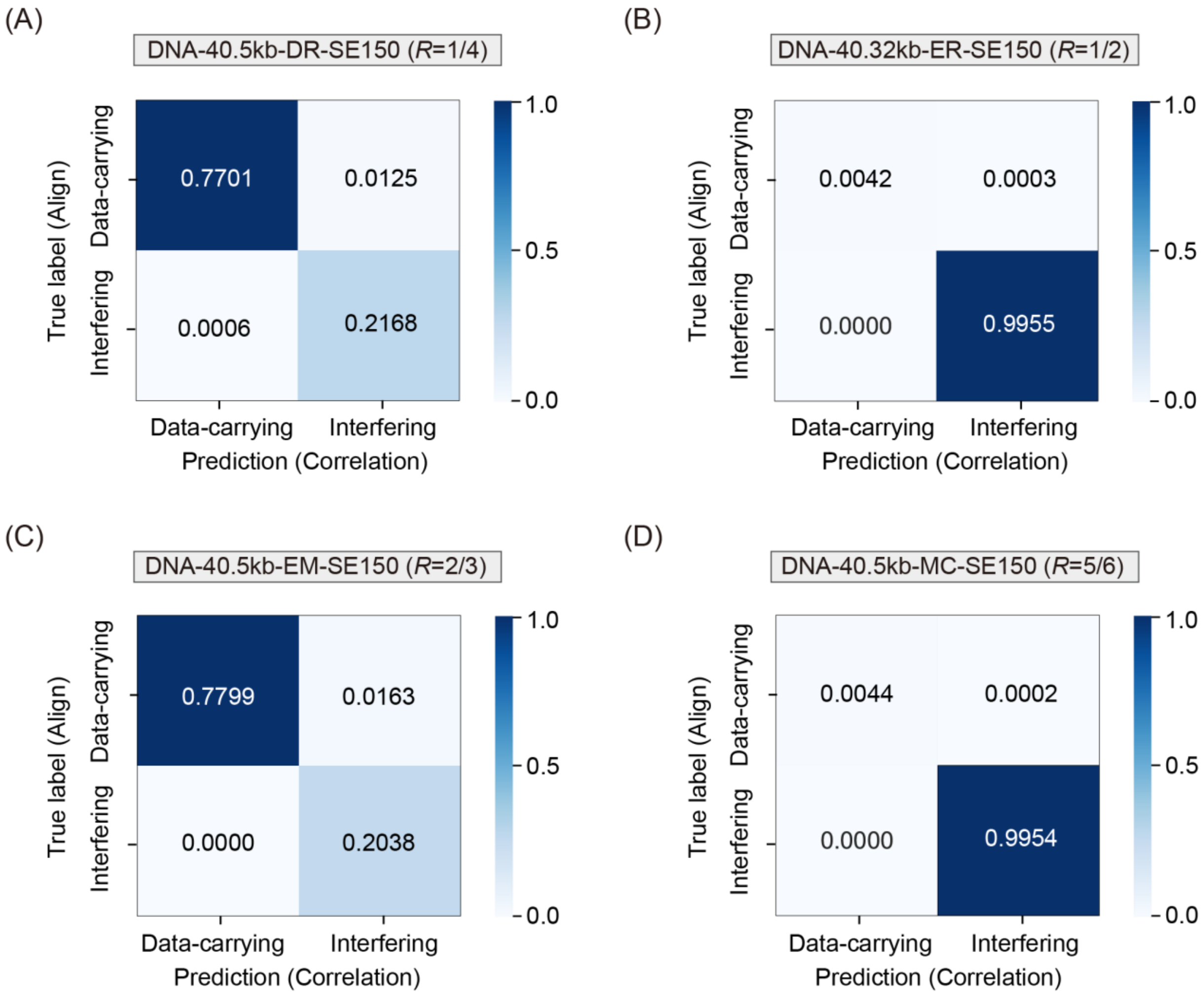

Figure S18 Accuracy of correlation-based classification of data-carrying and interfering reads. Confusion matrices show the agreement between correlation-based predictions and alignment-derived ground-truth labels. True labels are assigned through sequence alignment. (B) and (D) correspond to chromosome-based DNA storage with host genomic interference (R = 1/2 and R = 5/6).

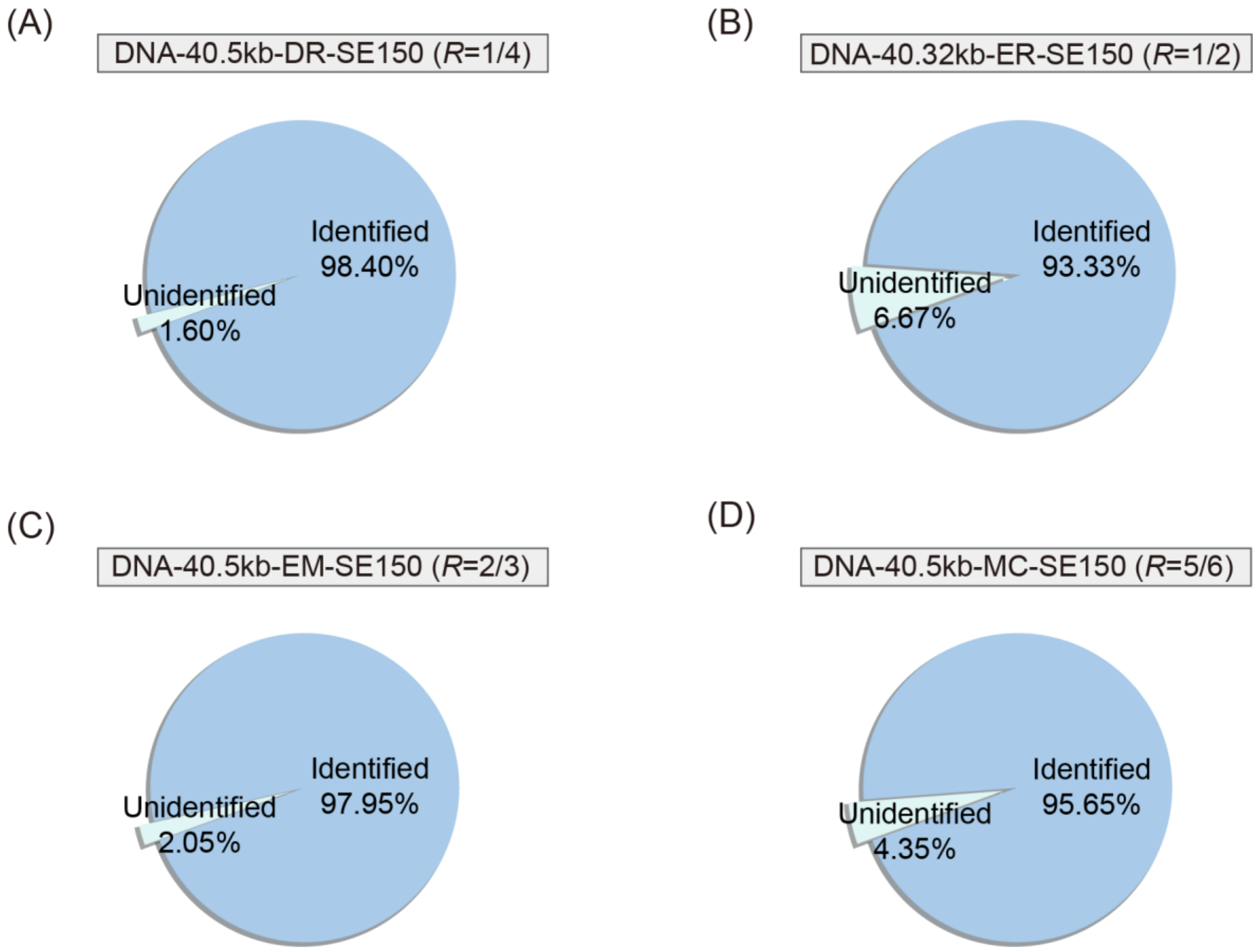

Figure S19 Read utilization of sliding correlation. Read utilization was defined as the proportion of data-carrying reads that could be successfully identified using the sliding correlation algorithm (i.e., those exhibiting correlation peaks above a predefined threshold). (A) DNA-40.5kb-DR-SE150 ($R = 1/4$). (B) DNA-40.32kb-ER-SE150 ($R = 1/2$). (C) DNA-40.5kb-EM-SE150 ($R = 2/3$). (D) DNA-40.5kb-MC-SE150 ($R = 5/6$).

(A)

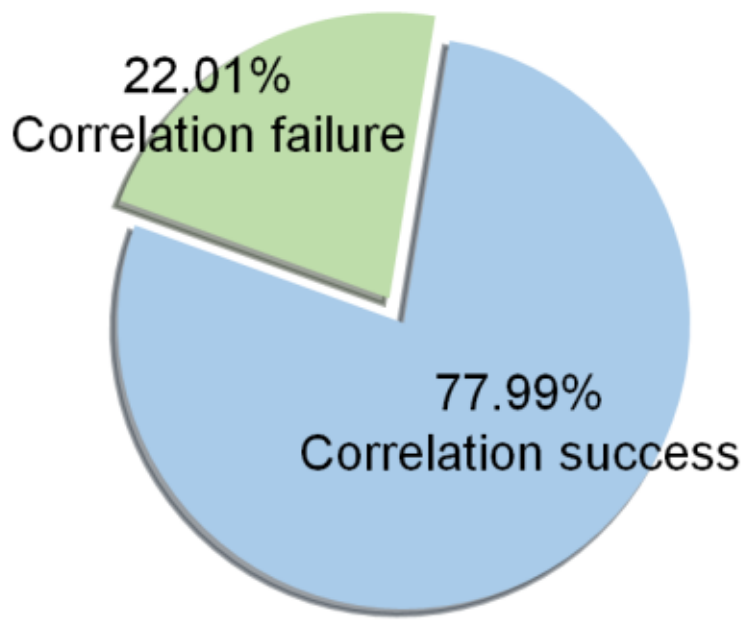

(B)

	Failure	Success
Ins.	0.2084	0.0001
Del.	0.0479	0.0001
Sub.	0.2092	0.0017
Total	0.4655	0.0019

(C) (D)

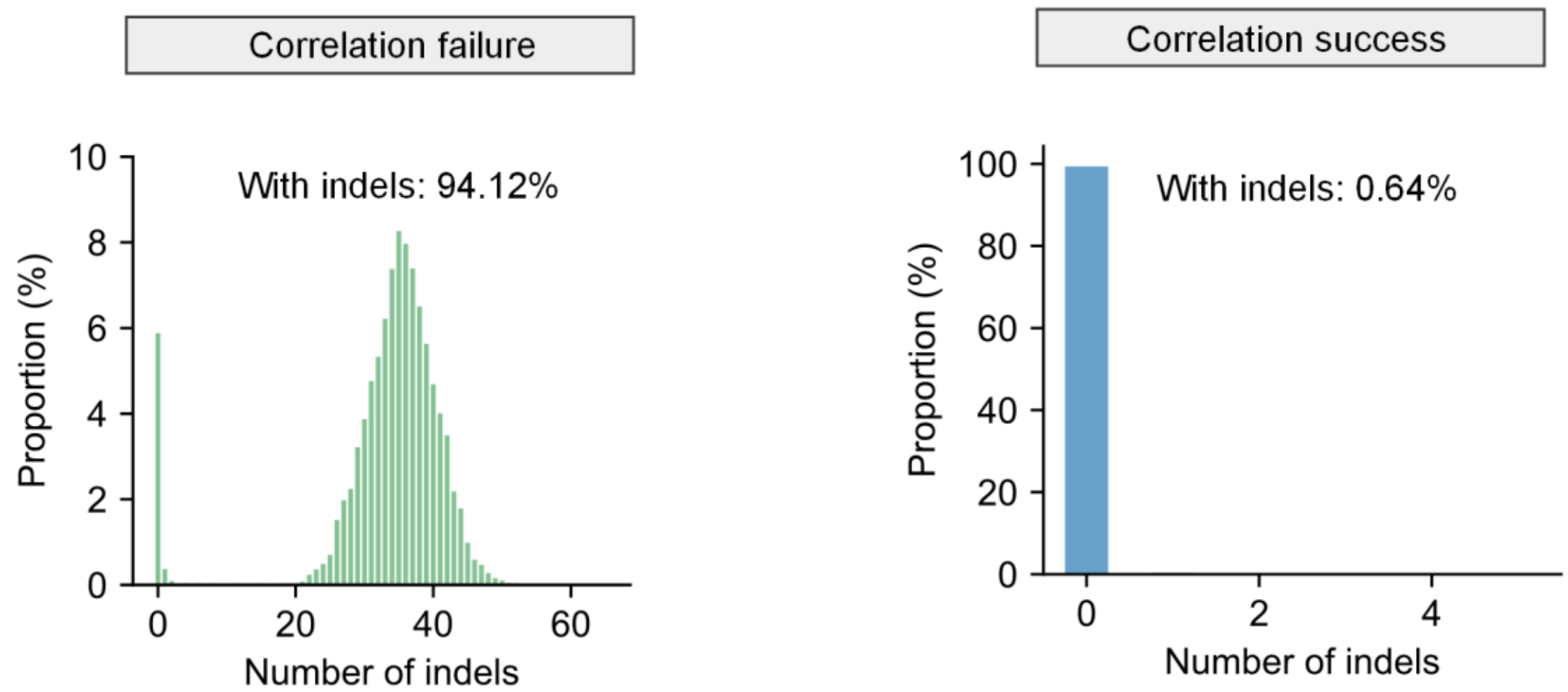

Figure S20 Proportion and error characteristics of data-carrying reads. Data-carrying reads were identified by sliding correlation using 150-nt reads from DNA-40.5kb-EM-SE150 (R = 2/3). (A) Proportion of data-carrying reads identified by sliding correlation. (B) Raw error rates of reads that passed versus failed the correlation threshold. (C) Indel error count distribution in reads that failed correlation. (D) Indel error count distribution in reads that passed correlation.

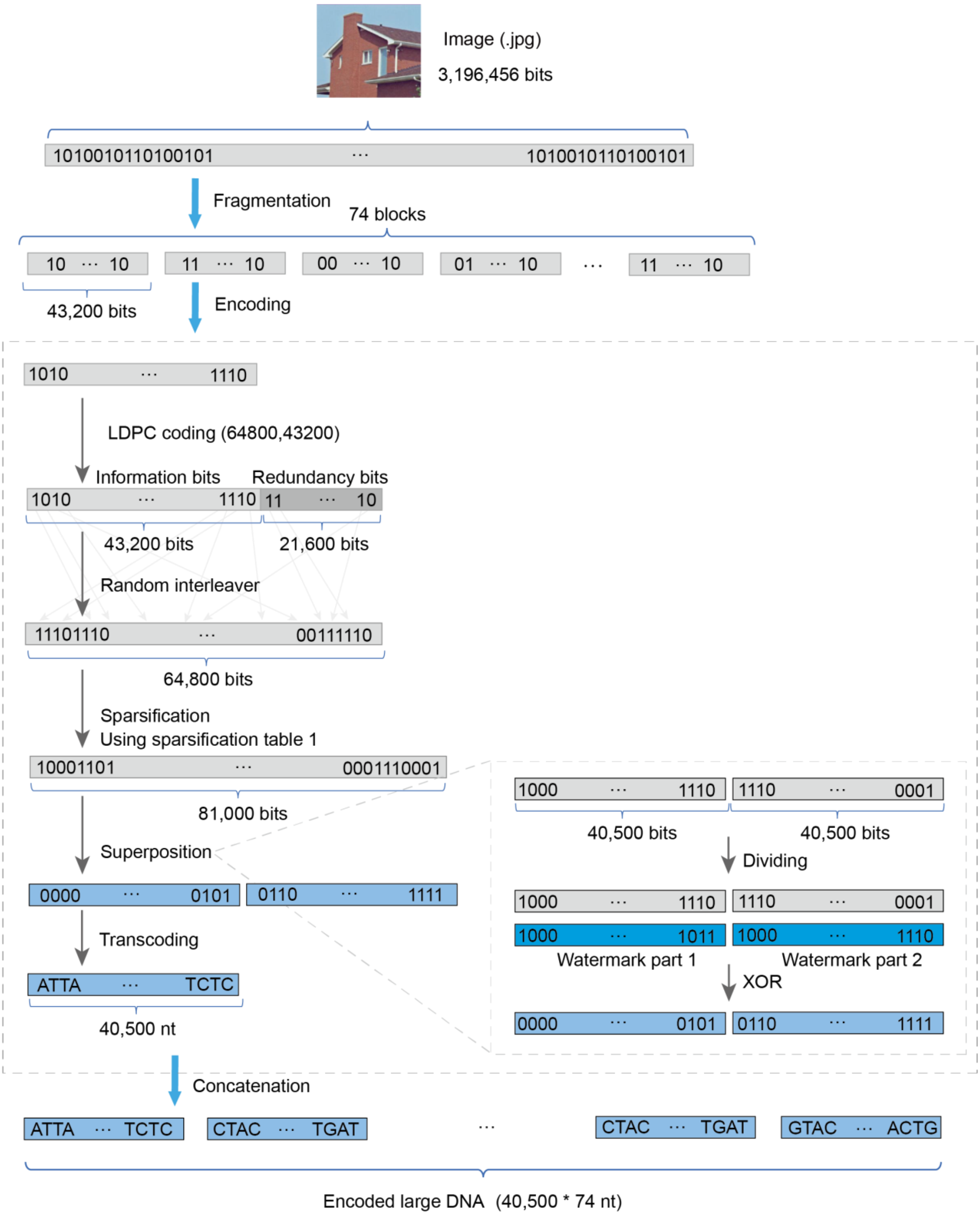

Figure S21 Large DNA encoding workflow used in simulation. The digital file was divided into 74 blocks. Each block was encoded with an LDPC code (R = 2/3) to generate 64,800 bits, then sparsified to 81,000 bits. The sparsified codewords were superimposed with a pseudo-random sequence (watermark) through an exclusive OR (XOR) operation. Next, the DNA sequences were obtained by transcoding every 2 bits into 1 base.

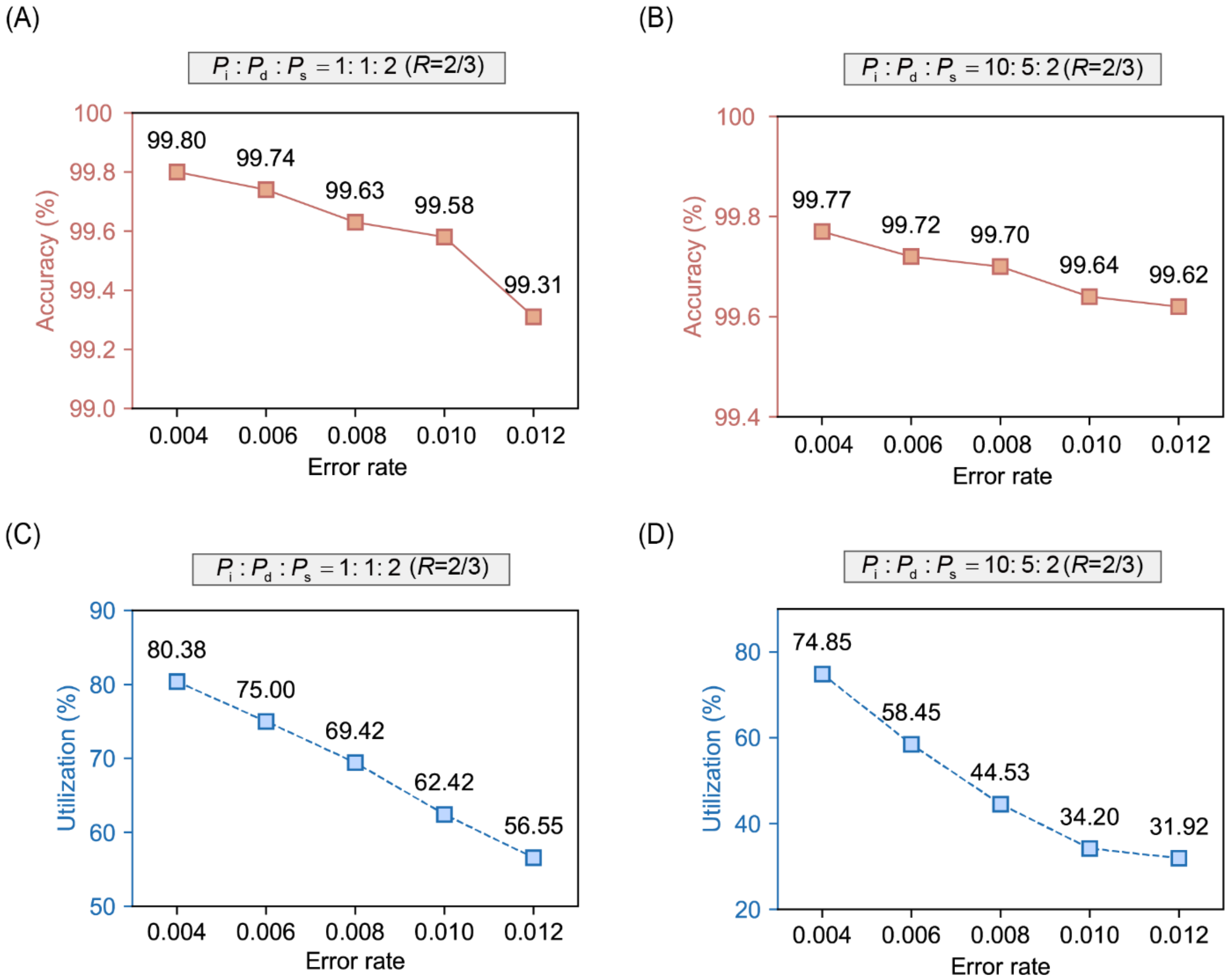

Figure S22 Impact of error profiles on positioning accuracy and read utilization. The performance of the sliding correlation method was evaluated using 100,000 simulated reads, derived from DNA-40.5 kb-EM-SE150 (R = 2/3). P_i, P_d, and P_s represent the error rates for insertions, deletions, and substitutions, respectively. (A–B) Positioning accuracy under two representative error profiles. (C–D) Corresponding read utilization under the same conditions.

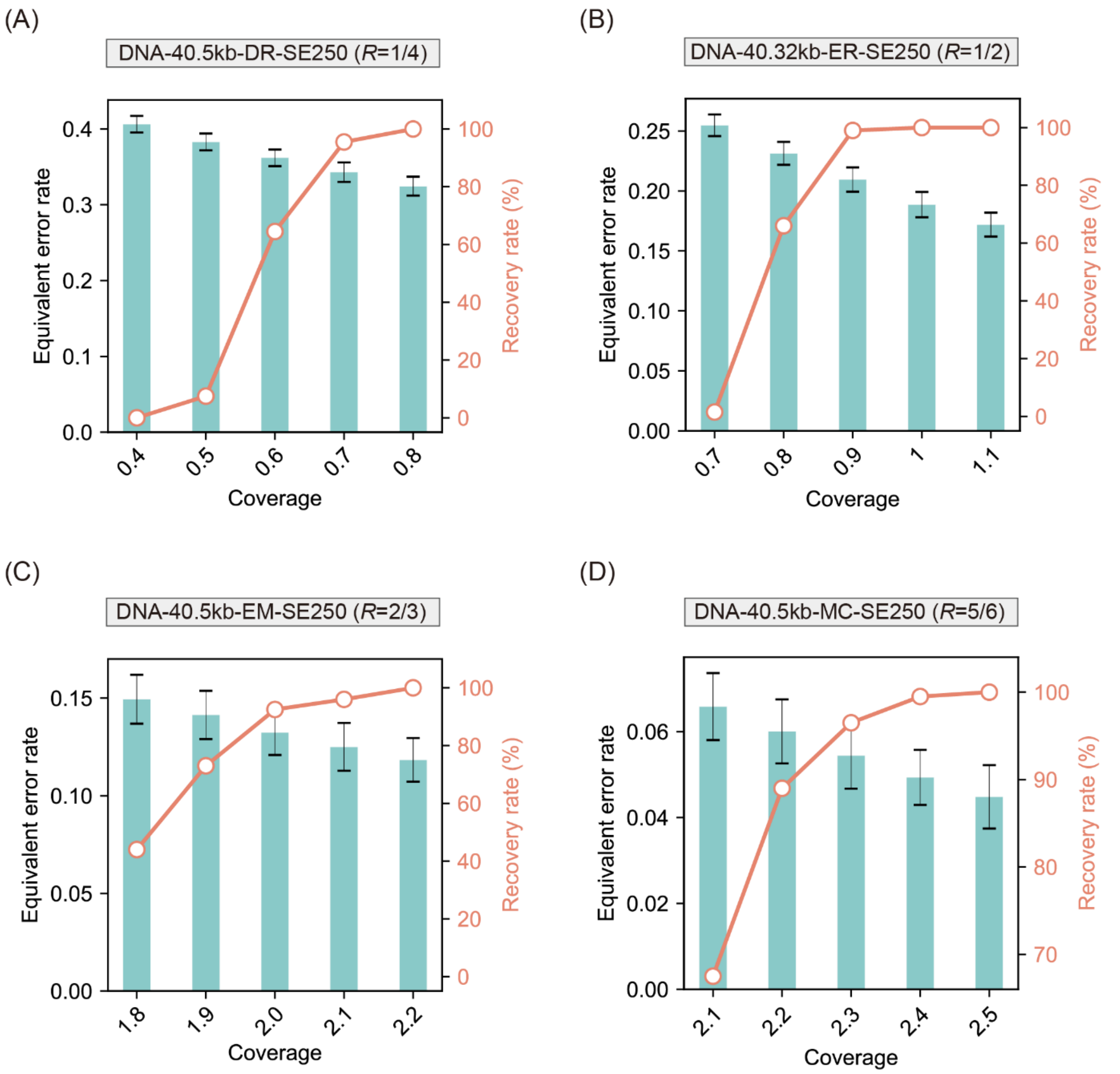

Figure S23 Data recovery performance using 250-bp NGS reads. Performance was evaluated for different LDPC coding schemes across a range of sequencing coverages, using 200 independent recovery experiments. Bar plots represent the equivalent error rate (mean ± SD), calculated as half the erasure rate plus the substitution error rate (i.e., $E_{\mathrm{erasure}}/2 + E_{\mathrm{error}}$). Line plots indicate the recovery ratio. (A) DNA-40.5 kb-DR-SE250 (R = 1/4) from Plasmid-A. (B) DNA-40.32 kb-ER-SE250 (R = 1/2). (C) DNA-40.5 kb-EM-SE250 (R = 2/3) from Plasmid-B. (D) DNA-40.5 kb-MC-SE250 (R = 5/6).

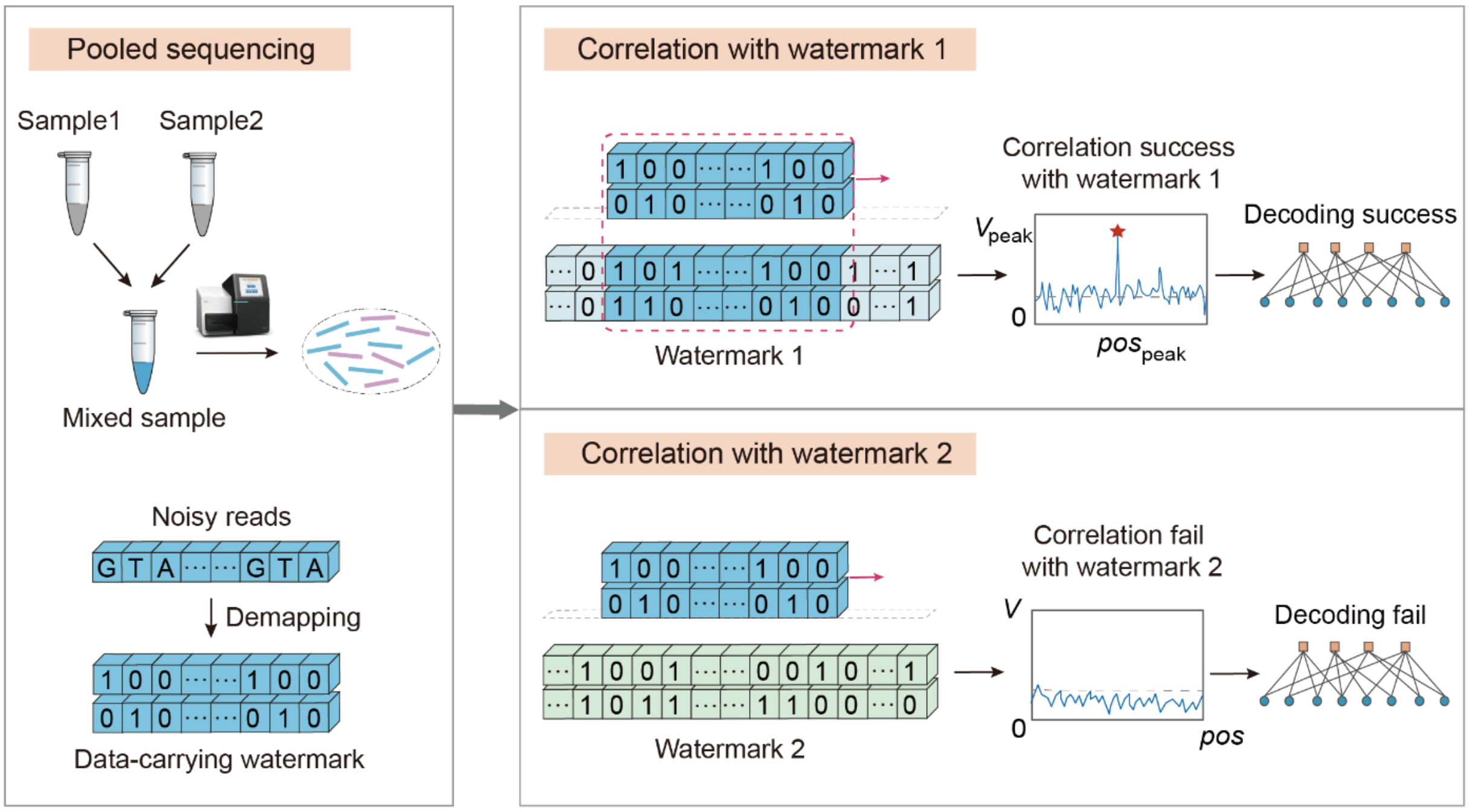

Figure S24 Distinguishing cross-fragment interference in pooled-fragment experiments using watermark-based correlation. The two samples, DNA-40.5kb-DR (R = 1/4) and DNA-40.5kb-EM (R = 2/3), were pooled and sequenced on the Illumina HiSeq platform. Sequencing reads were subjected to sliding correlation based on multiple watermark sequences. Since each plasmid was embedded with a distinct watermark sequence, the correlation-based classification distinguished reads originating from the two plasmids.

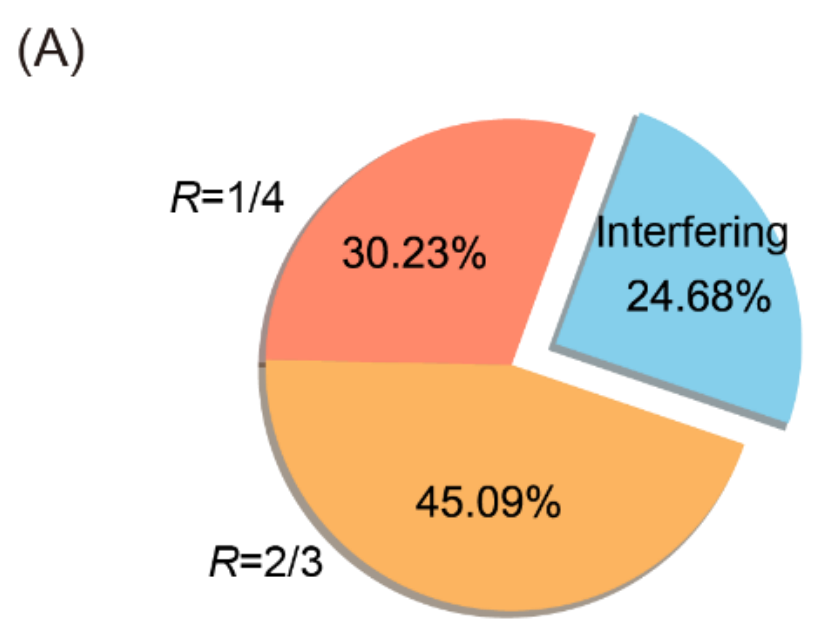

(B)

Error rate	Pooled sequencing	Unpooled sequencing	
		R=1/4	R=2/3
Ins.	0.0002	0.00016	0.00019
Del.	0.0001	0.00003	0.00004
Sub.	0.0011	0.00158	0.00137
Total.	0.0014	0.00177	0.00160

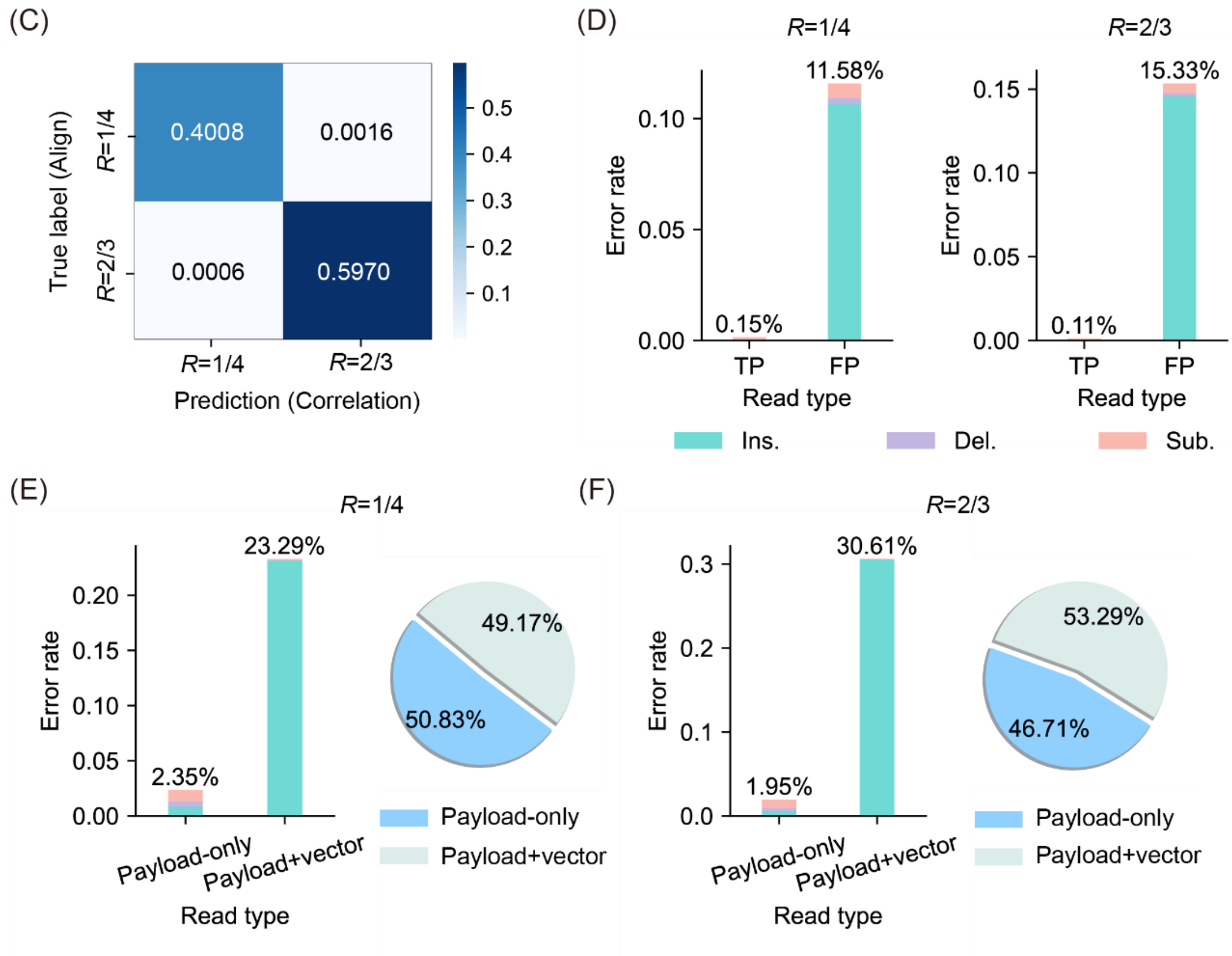

Figure S25 Recovery performance under pooled sequencing. (A) Composition of read types in the pooled-fragment experiments. (B) Comparison of raw error rates between pooled and unpooled sequencing. (C) Accuracy of the correlation-based classification. The true labels were obtained from sequence alignment, while the predicted labels were derived from the correlation results. (D) Raw error rates of different read types identified by correlation. True-positive (TP) and false-positive (FP) denote reads derived from the target fragments and from non-target fragments, respectively. (E) Composition and error rates of FP reads from DNA-40.5kb-DR (R = 1/4). The misclassified reads comprised two types: payload-only reads and payload + vector reads, the latter carrying vector sequences that resulted in higher insertion error rates. (F) Composition and error rates of FP reads from DNA-40.5kb-EM (R = 2/3).

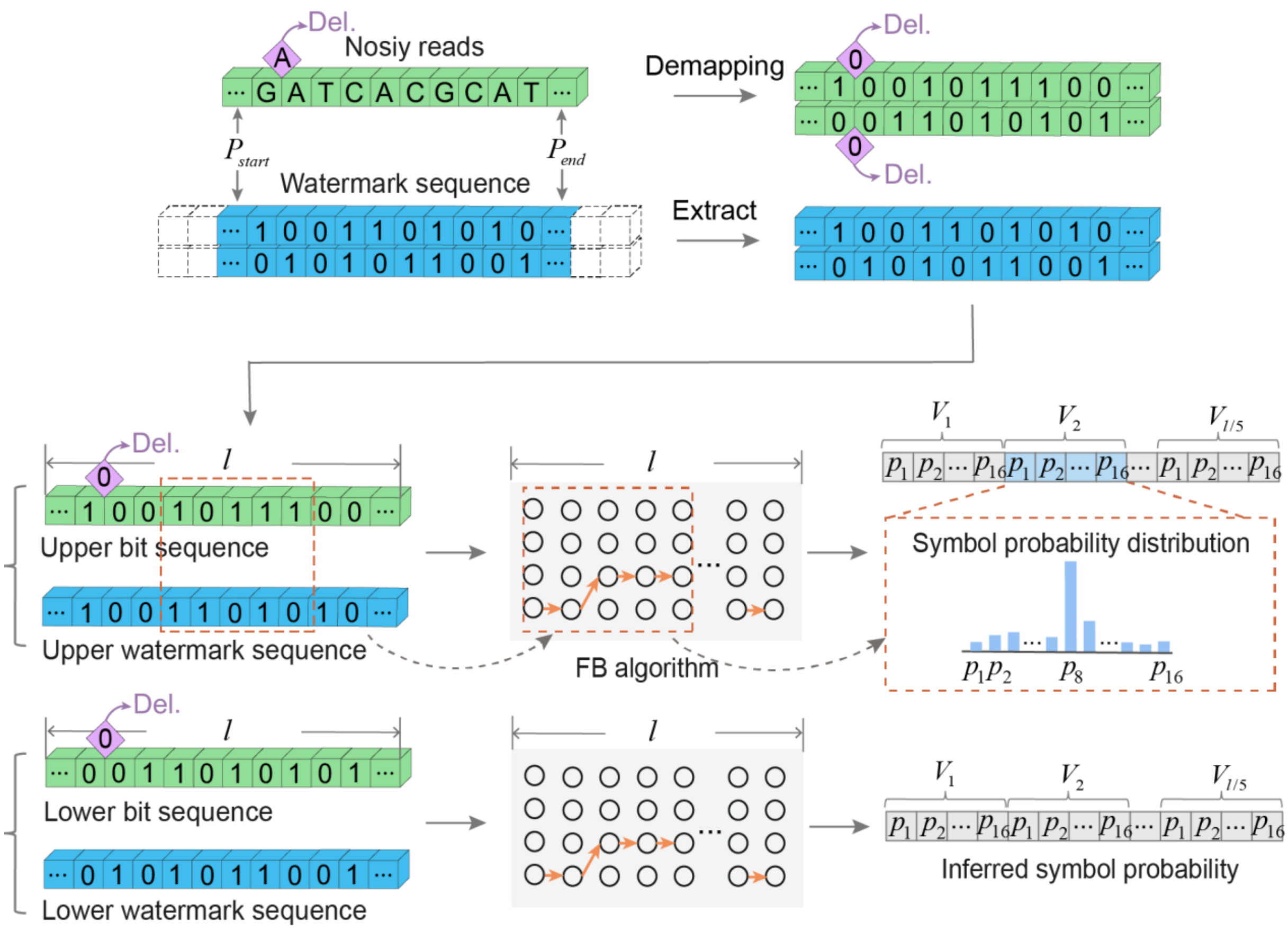

Figure S26 Workflow of the read-by-read forward-backward algorithm. Noisy reads are demapped into upper and lower bit sequences. Based on the pre-identified start position on the watermark sequence (obtained via alignment or correlation), the corresponding watermark segments are extracted. Each bit layer, along with its associated watermark segment, is then processed using the FBA to correct indel errors and estimate symbol probabilities.

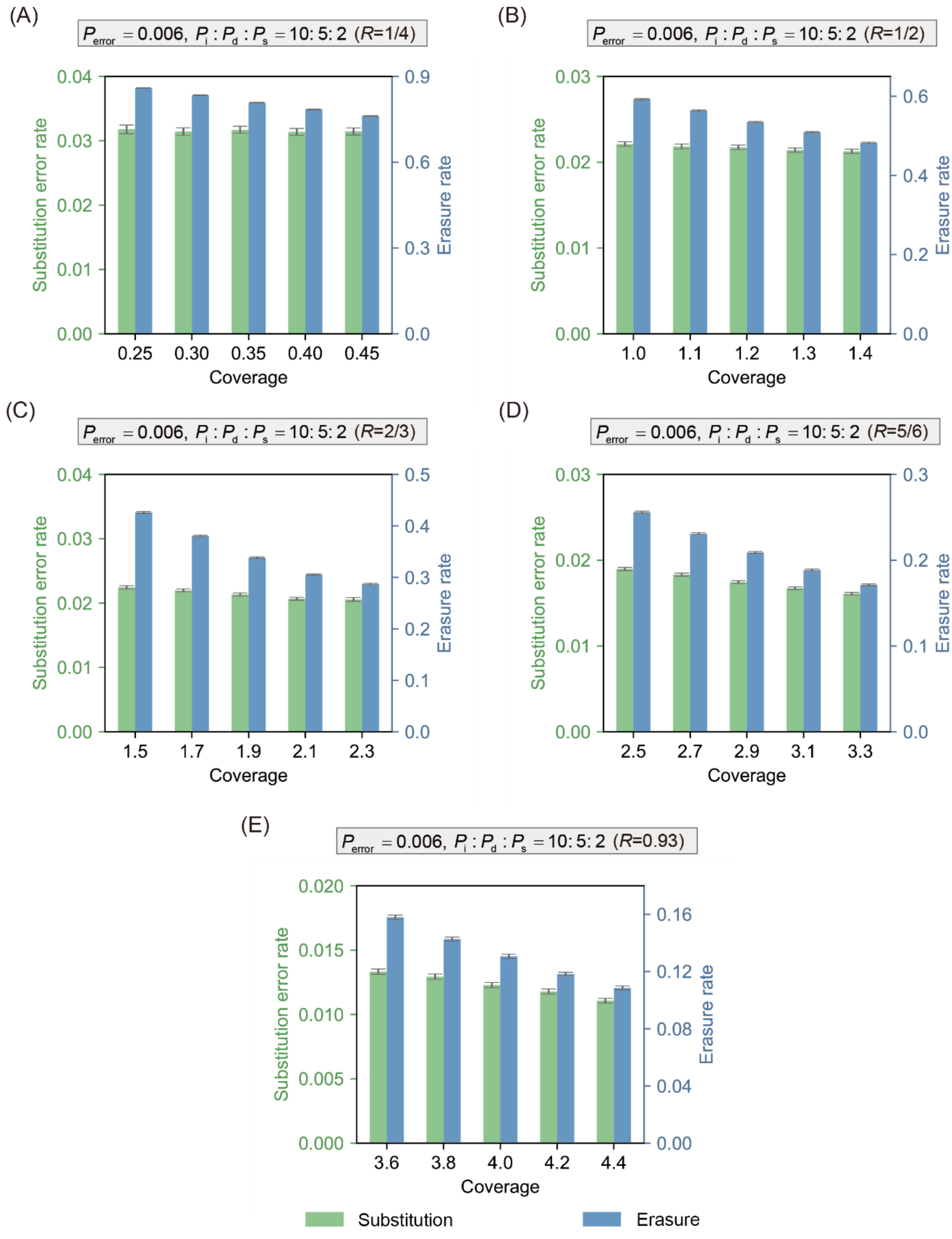

Figure S27 Base erasure rate and base substitution error rate of the scaffold reference (simulated data, raw error rate = 0.6%). Blue and green bars represent base erasure and substitution rates, respectively. Error bars denote standard deviations from 200 independent simulations. (A) DNA-40.5kb-DR-Sim-1 (R = 1/4). (B) DNA-40.32kb-ER-Sim-1 (R = 1/2). (C) DNA-40.5kb-EM-Sim-1 (R = 2/3). (D) DNA-40.5kb-MC-Sim-1 (R = 5/6). (E) DNA-20kb-RB-Sim-1 (R = 0.93).

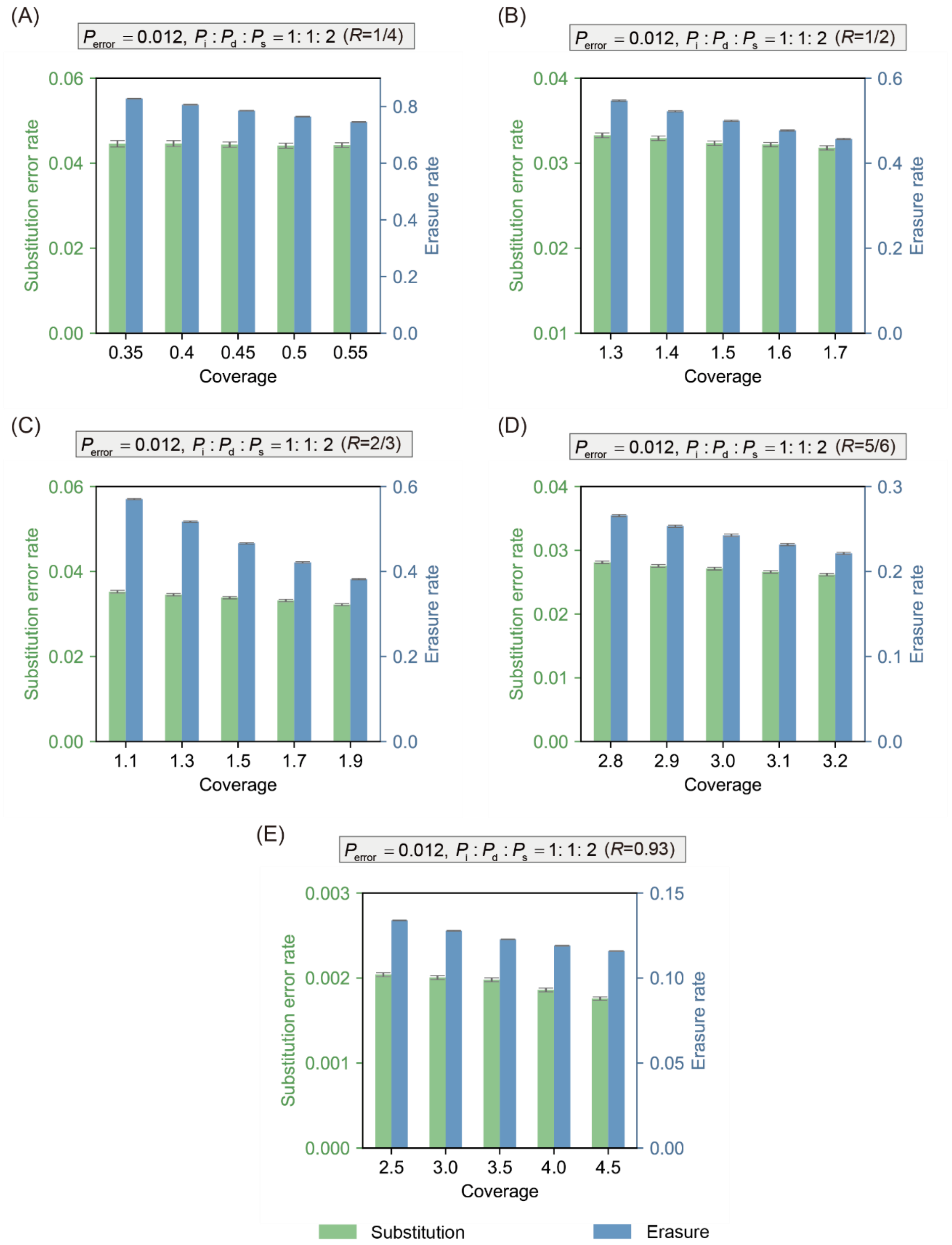

Figure S28 Base erasure rate and base substitution error rate of the scaffold reference (simulated data, raw error rate = 1.2%). Blue and green bars represent base erasure and substitution rates, respectively. Error bars denote standard deviations from 200 independent simulations. (A) DNA-40.5kb-DR-Sim-2 (R = 1/4). (B) DNA-40.32kb-ER-Sim-2 (R = 1/2). (C) DNA-40.5kb-EM-Sim-2 (R = 2/3). (D) DNA-40.5kb-MC-Sim-2 (R = 5/6). (E) DNA-20kb-RB-Sim-2 (R = 0.93).

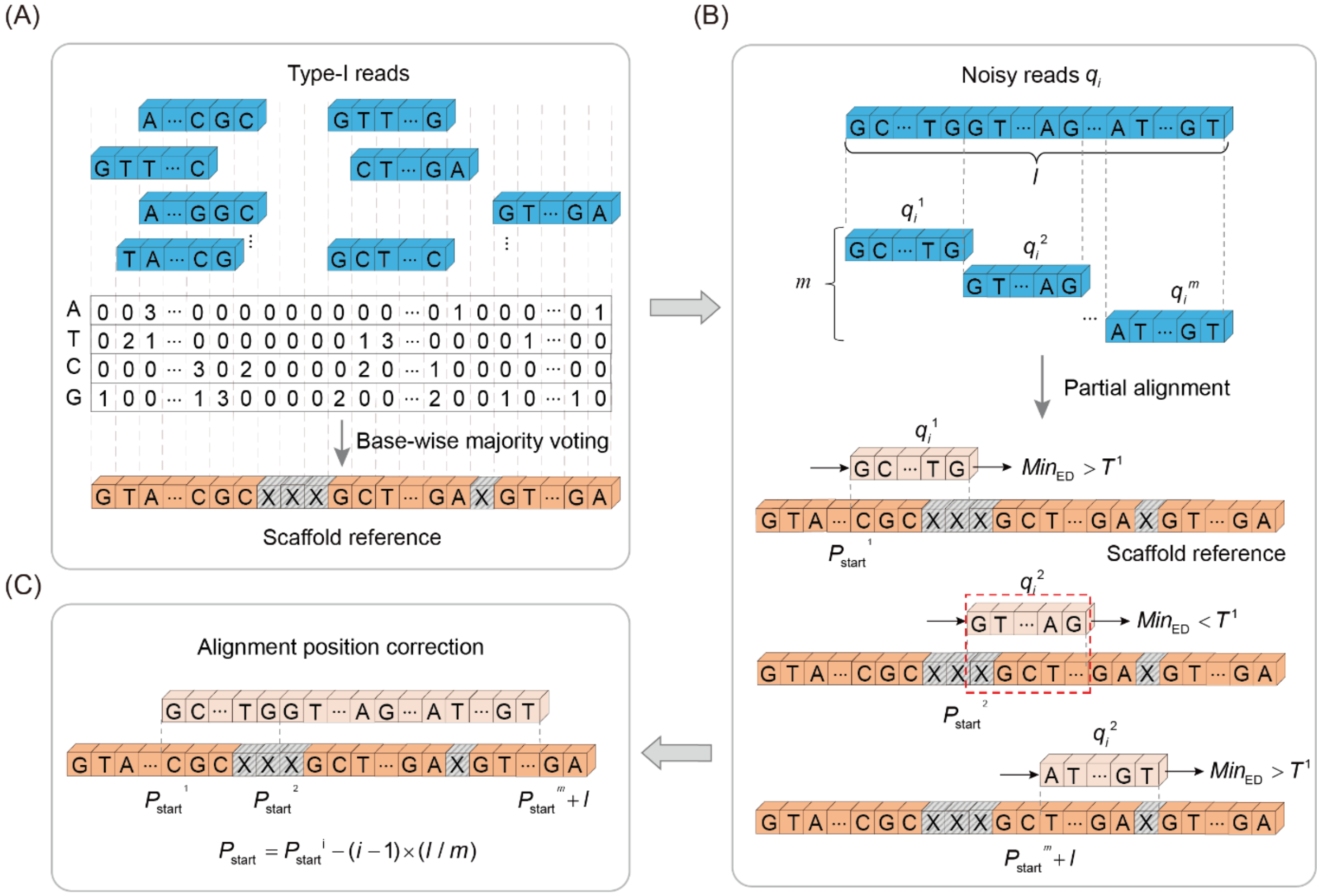

Figure S29 Workflow of the partial alignment method. The partial alignment method consists of three main steps. (A) Type-I reads that pass initial identification are aligned to generate a consensus scaffold through base-wise majority voting. Positions with no read coverage are denoted as "X". (B) Reads that fail the initial correlation-based identification are split into equal-length segments and aligned independently to the scaffold. If any segment achieves an edit distance below a predefined threshold, the read is reclassified as Type-II. (C) The approximate mapping position of Type-II reads is estimated based on the index and alignment position of the matched segment, followed by position correction.

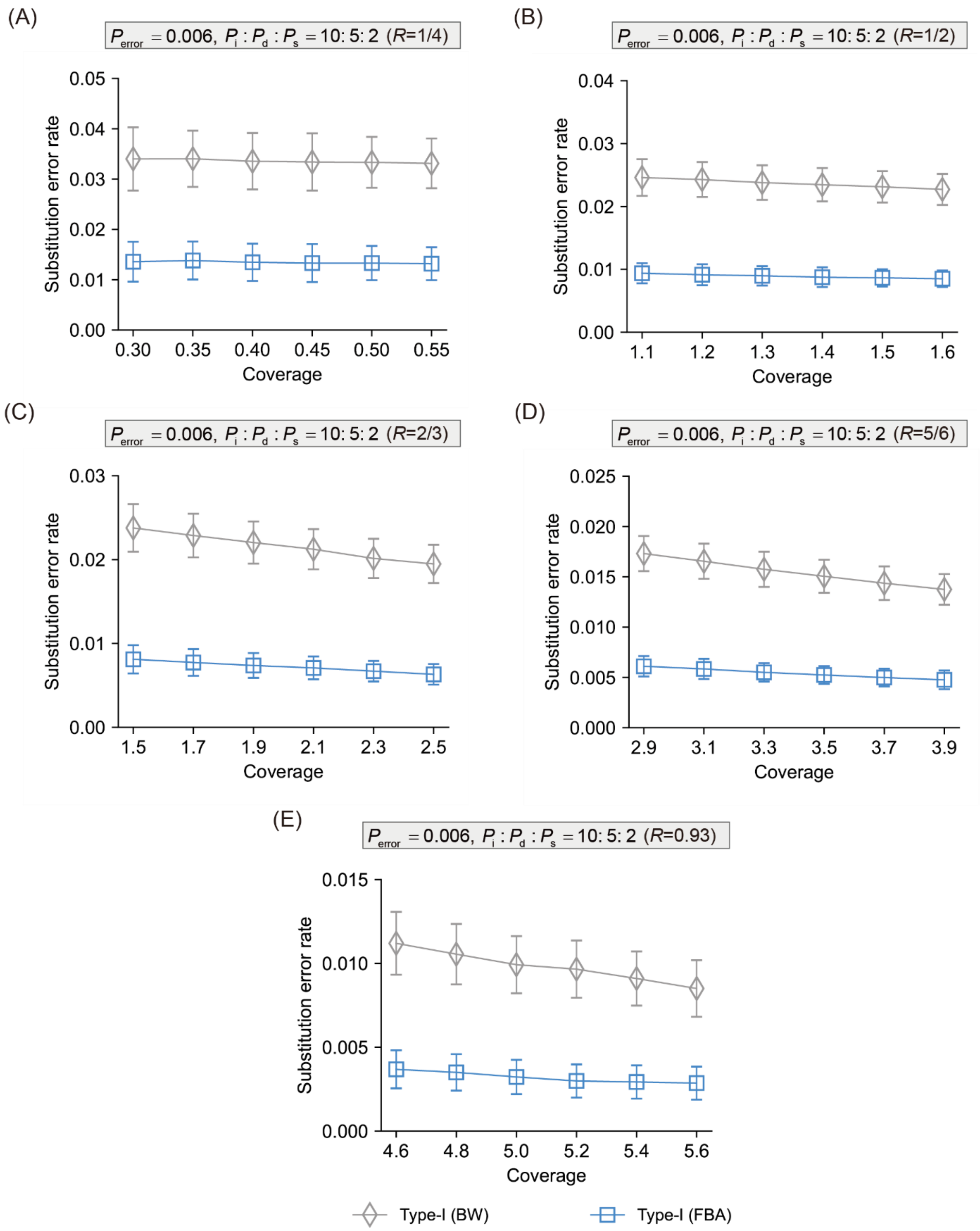

Figure S30 Evaluation of the probability generation with FBA. Compared to direct bit-wise consensus (BW), probability generation with FBA significantly reduced the number of substitution errors before decoding (raw error rate = 0.6%). (A) DNA-40.5kb-DR-Sim-1 (*R* = 1/4). (B) DNA-40.32kb-ER-Sim-1 (*R* = 1/2). (C) DNA-40.5kb-EM-Sim-1 (*R* = 2/3). (D) DNA-40.5kb-MC-Sim-1 (*R* = 5/6). (E) DNA-20kb-RB-Sim-1 (*R* = 0.93).

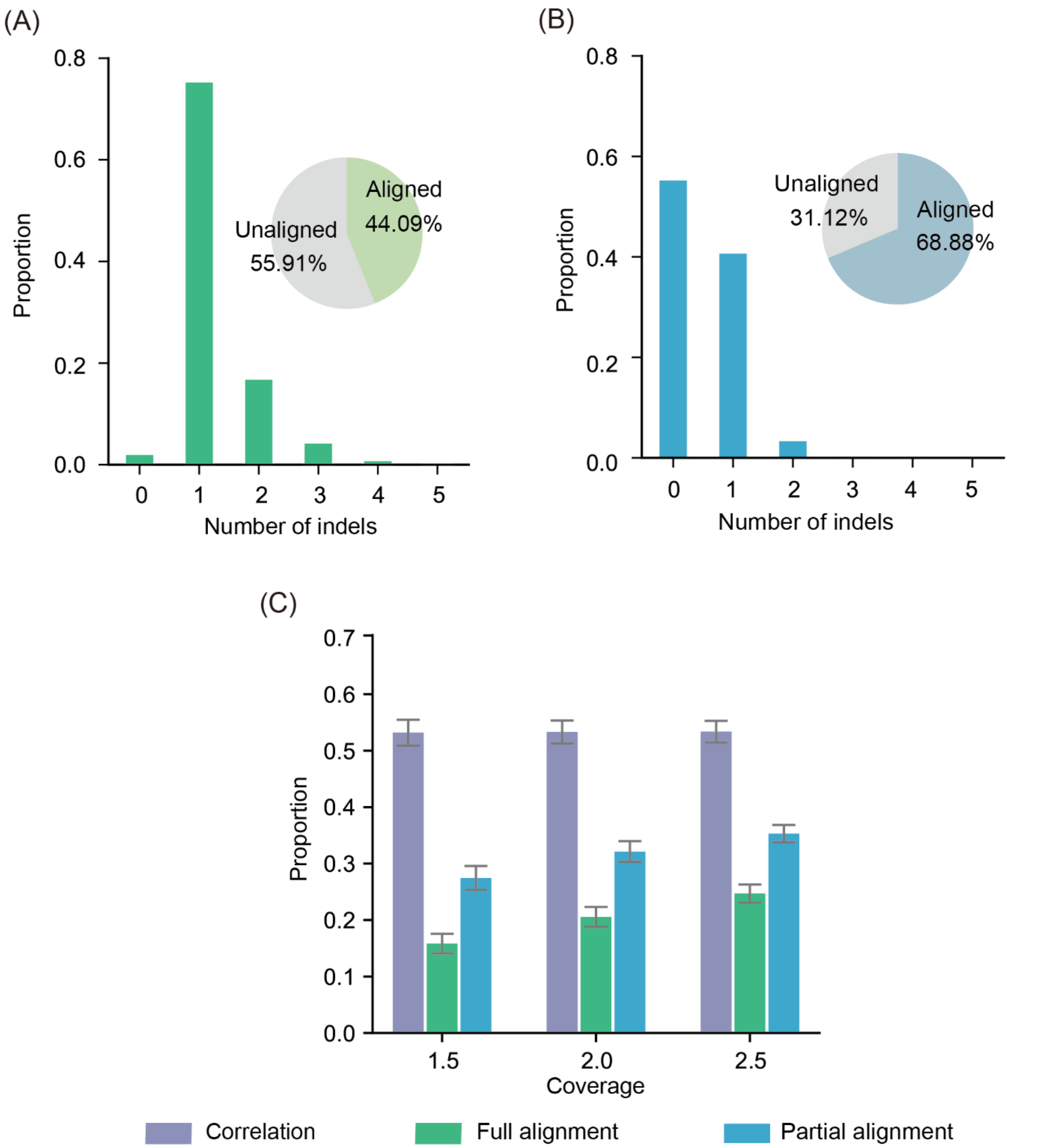

Figure S31 Evaluation of partial alignment on DNA-40.32kb-ER-Sim-1 (*R* = 1/2). Read identification via partial alignment was benchmarked against full-length alignment. (A and B) Indel error count distributions of reads recovered by full alignment and partial alignment, respectively. The accompanying pie charts show identification rates: 44.09% for full alignment and 68.88% for partial alignment. (C) Proportions of reads identified through correlation, full alignment, and partial alignment across different sequencing coverages.

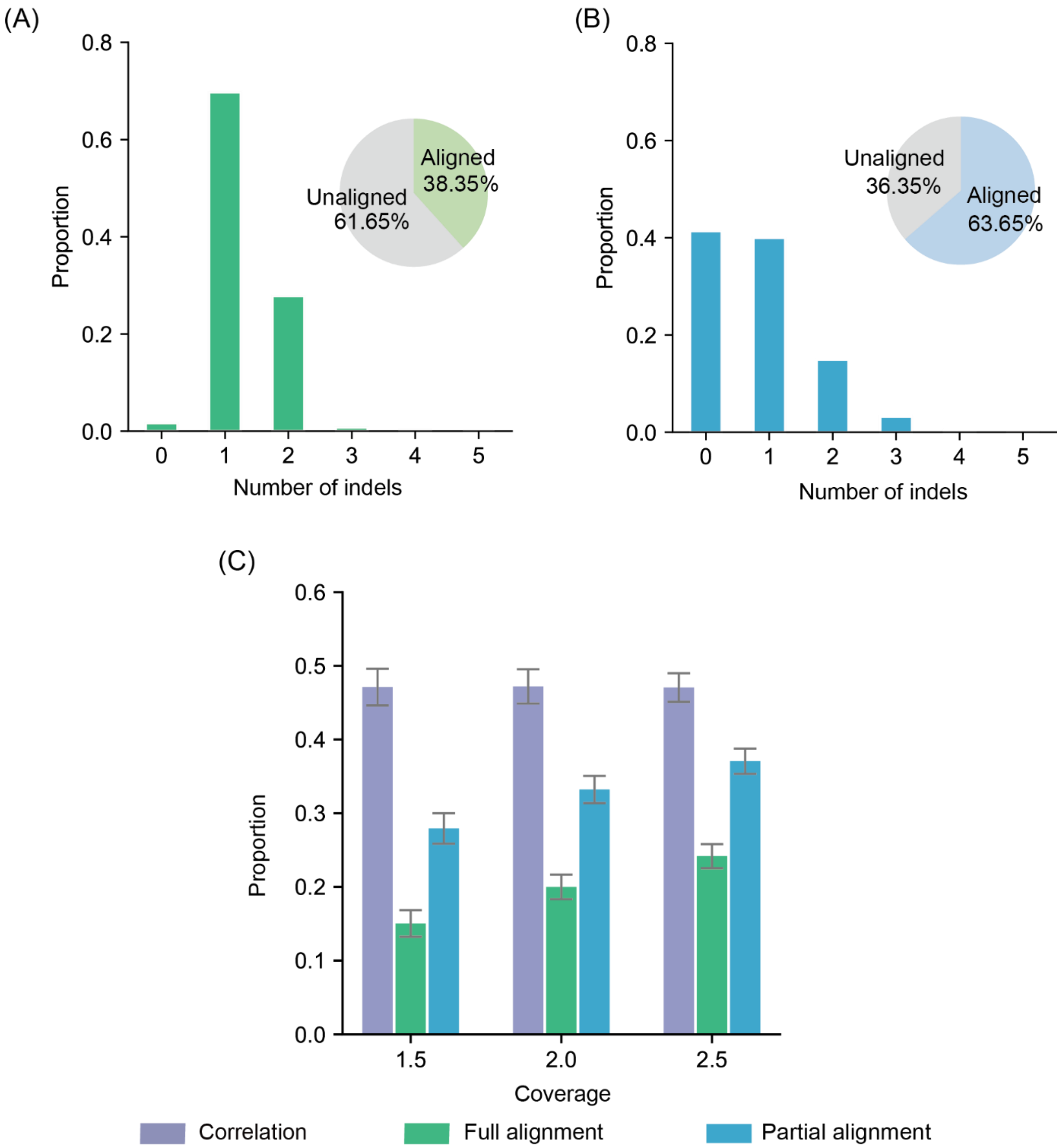

Figure S32 Evaluation of partial alignment on DNA-40.32kb-ER-Sim-2 (*R* = 1/2). Read identification via partial alignment was benchmarked against full-length alignment. (A and B) Indel error count distributions of reads recovered by full alignment and partial alignment, respectively. The accompanying pie charts show identification rates: 38.35% for full alignment and 63.65% for partial alignment. (C) Proportions of reads identified through correlation, full alignment, and partial alignment across different sequencing coverages.

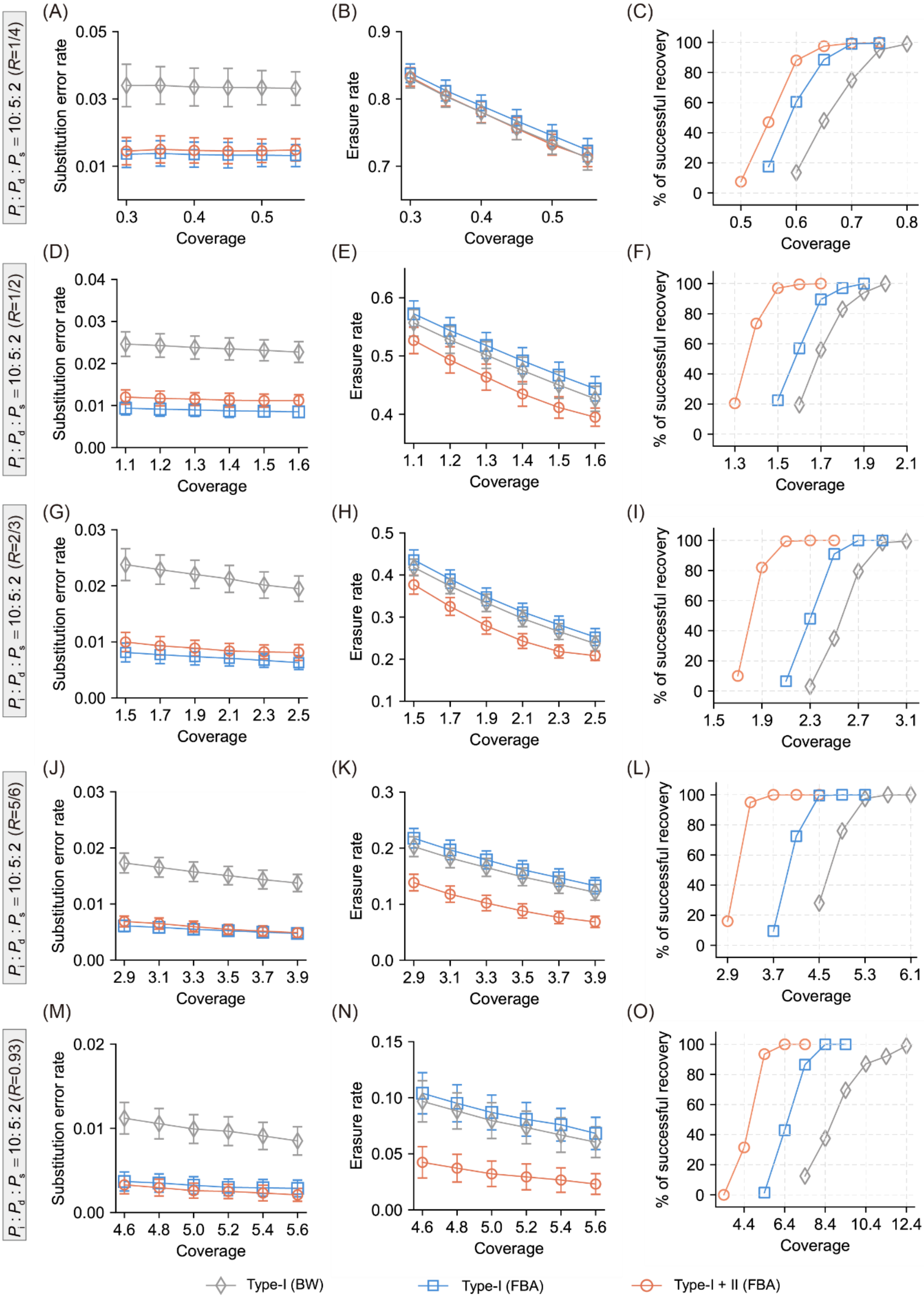

Figure S33 Evaluation of data recovery using different types of reads and soft-decision information generation schemes (raw error rate = 0.6%). Substitution and erasure rates before LDPC decoding, as well as recovery performance, were evaluated under various code rates across 200 trials.

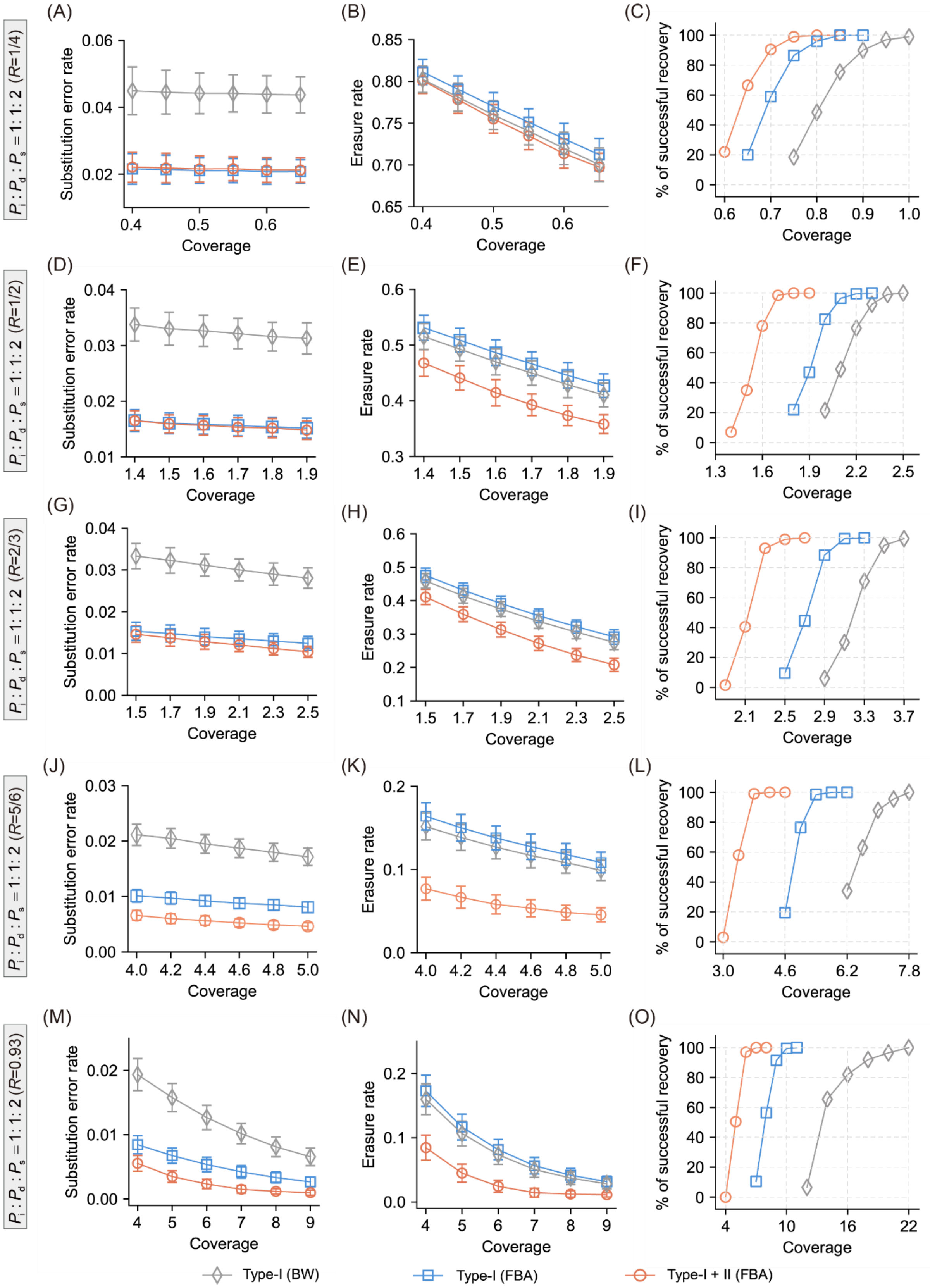

Figure S34 Evaluation of data recovery using different types of reads and soft-decision information generation schemes (raw error rate = 1.2%). Substitution and erasure rates before LDPC decoding, as well as recovery performance, were evaluated under various code rates across 200 trials.

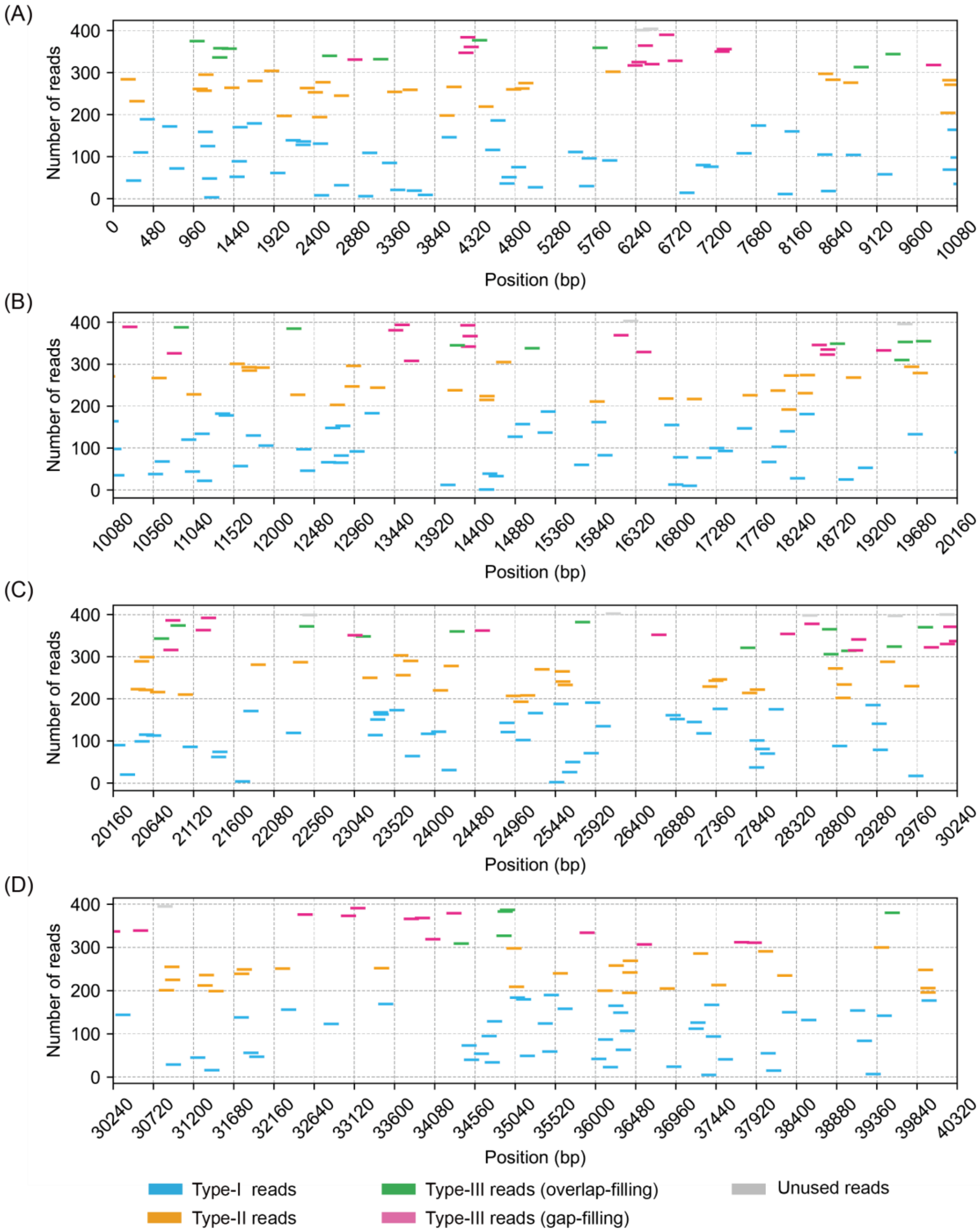

Figure S35 Mapping positions of simulated reads on the large-fragment DNA. A total of 404 simulated reads were generated from DNA-40.32kb-ER-Sim-1 ($R = 1/2$) with a raw error rate of 0.6%. The x-axis represents positions on the large-fragment DNA, while the y-axis corresponds to individual read indices. The Type-II reads were typically mapped to overlapping regions, whereas the Type-III reads were able to fill the gaps. (A–D) Correspond to regions 0–10,080 bp, 10,081–20,160 bp, 20,161–30,240 bp, and 30,241–40,320 bp, respectively.

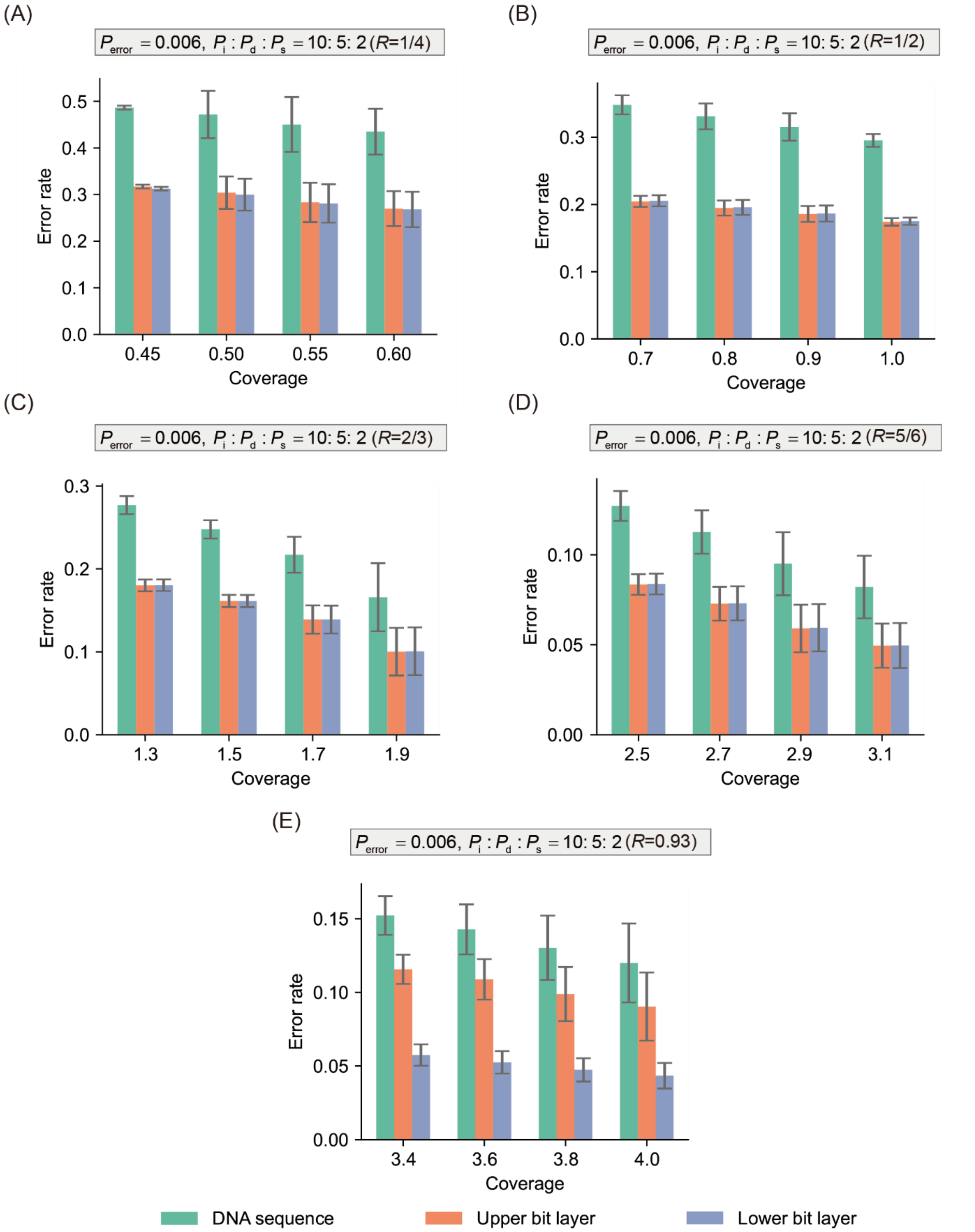

Figure S36 Error rates of the regenerative reference under varying sequencing coverages (raw error rate = 0.6%). The error rates were evaluated separately for the DNA sequence layer, the upper bit layer, and the lower bit layer under various code rates across 200 trials. (A) DNA-40.5kb-DR-Sim-1 (R = 1/4). (B) DNA-40.32kb-ER-Sim-1 (R = 1/2). (C) DNA-40.5kb-EM-Sim-1 (R = 2/3). (D) DNA-40.5kb-MC-Sim-1 (R = 5/6). (E) DNA-20kb-RB-Sim-1 (R = 0.93).

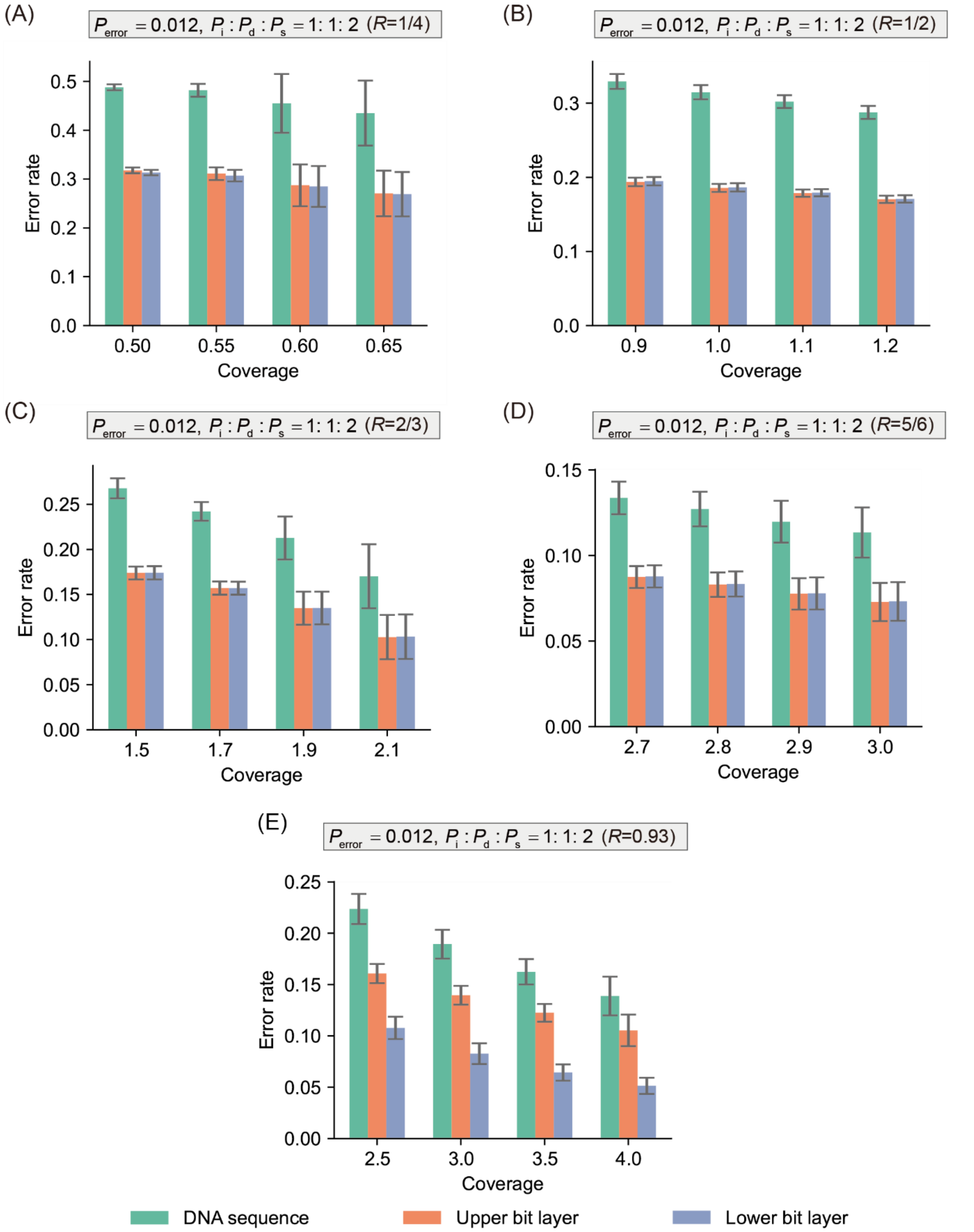

Figure S37 Error rates of the regenerative reference under varying sequencing coverages (raw error rate = 1.2%). The error rates were evaluated separately for the DNA sequence layer, the upper bit layer, and the lower bit layer under various code rates across 200 trials. (A) DNA-40.5kb-DR-Sim-2 (R = 1/4). (B) DNA-40.32kb-ER-Sim-2 (R = 1/2). (C) DNA-40.5kb-EM-Sim-2 (R = 2/3). (D) DNA-40.5kb-MC-Sim-2 (R = 5/6). (E) DNA-20kb-RB-Sim-2 (R = 0.93).

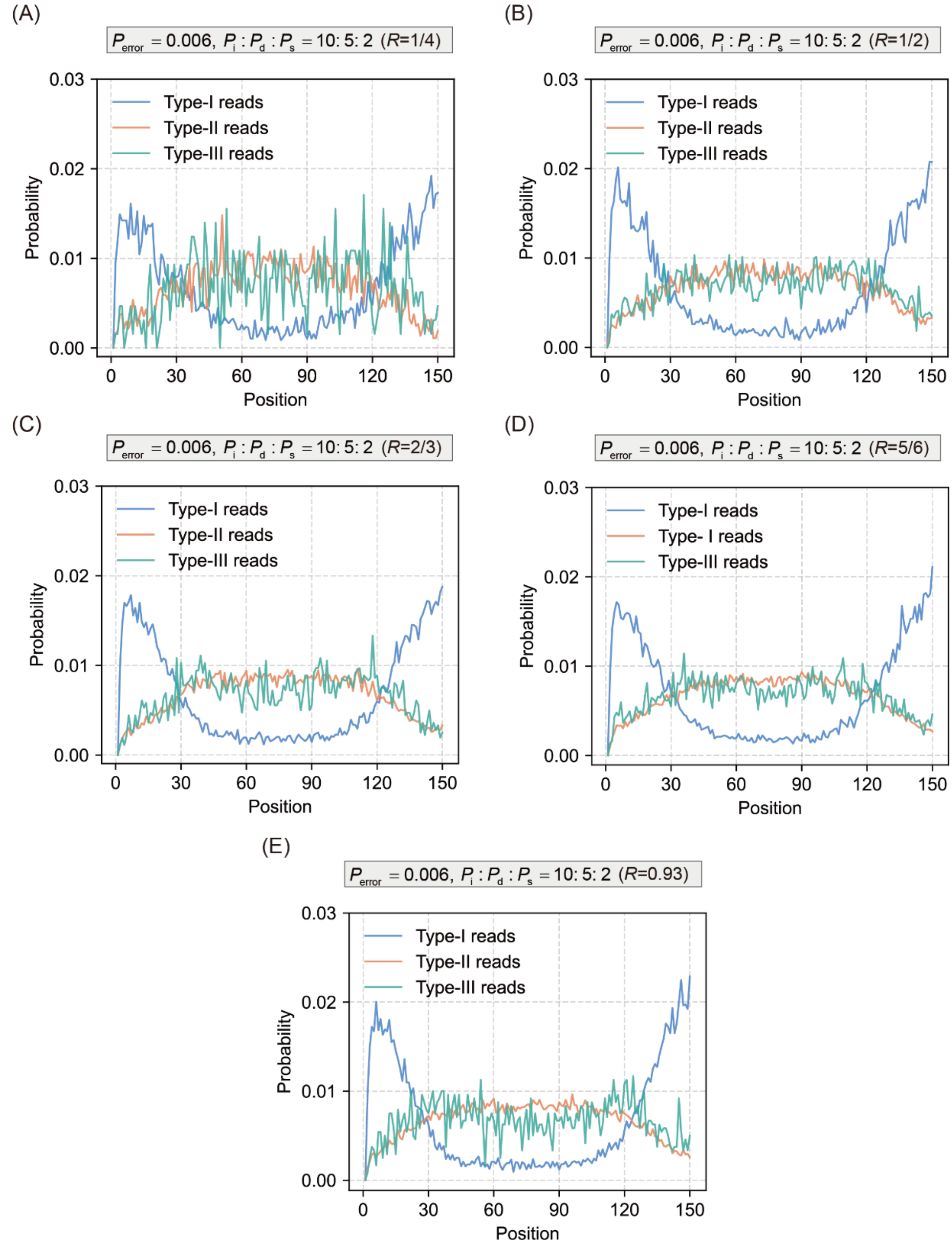

Figure S38 Distribution of indel error positions across types of reads (raw error rate = 0.6%). (A) The results for DNA-40.5kb-DR-Sim-1 (*R* = 1/4) were obtained at 0.5× coverage using 159 independent experiments. (B) The results for DNA-40.32kb-ER-Sim-1 (*R* = 1/2) were obtained at 1.3× coverage using 185 independent experiments. (C) The results for DNA-40.5kb-EM-Sim-1 (*R* = 2/3) were obtained at 1.7× coverage using 200 independent experiments. (D) The results for DNA-40.5kb-MC-Sim-1 (*R* = 5/6) were obtained at 2.9× coverage using 168 independent experiments. (E) The results for DNA-20kb-RB-Sim-1 (*R* = 0.93) were obtained at 4× coverage using 186 independent experiments.

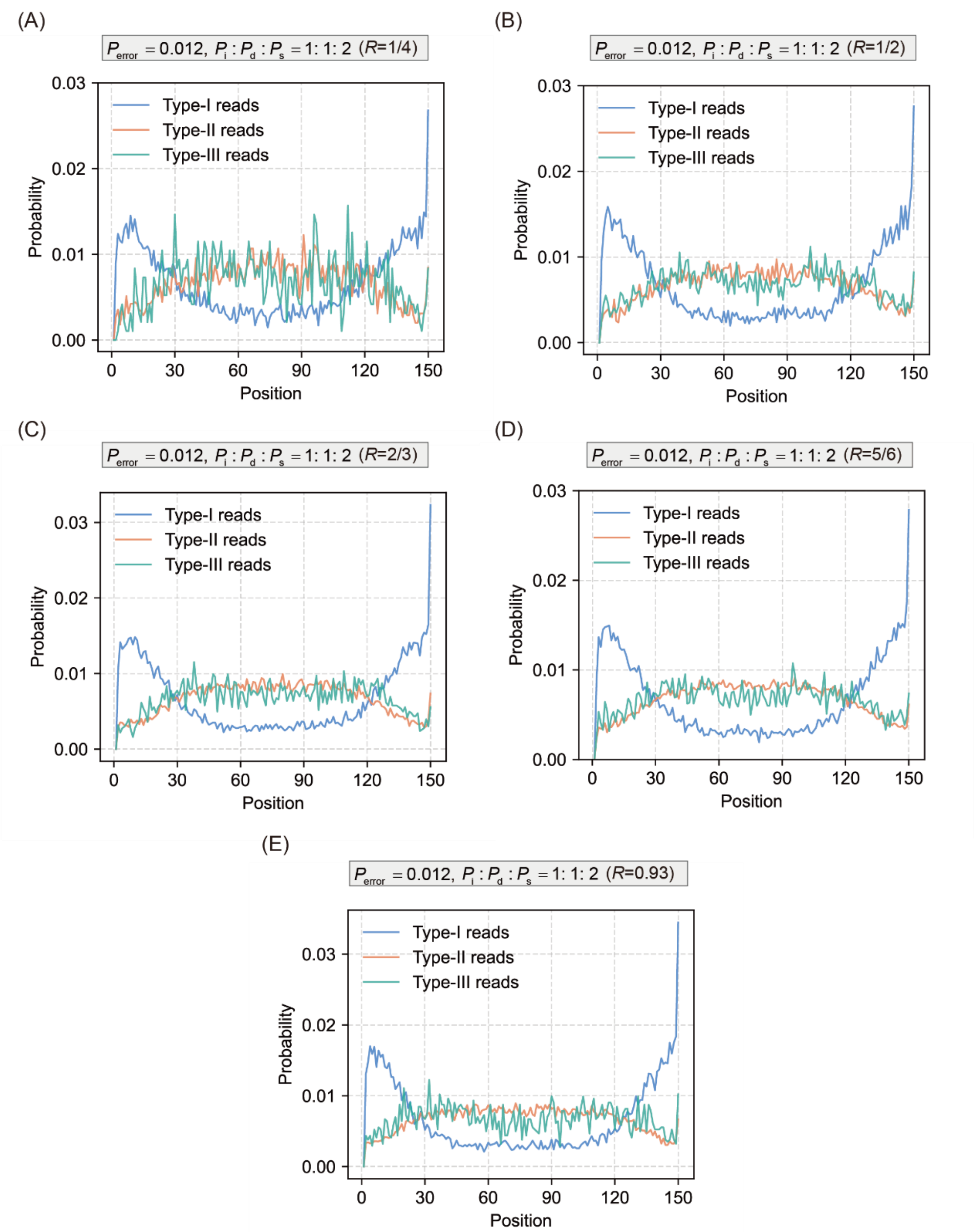

Figure S39 Distribution of indel error positions across types of reads (raw error rate = 1.2%). (A) The results for DNA-40.5kb-DR-Sim-2 (R = 1/4) were obtained at 0.6× coverage using 162 independent experiments. (B) The results for DNA-40.32kb-ER-Sim-2 (R = 1/2) were obtained at 1.5× coverage using 130 independent experiments. (C) The results for DNA-40.5kb-EM-Sim-2 (R = 2/3) were obtained at 1.9× coverage using 197 independent experiments. (D) The results for DNA-40.5kb-MC-Sim-2 (R = 5/6) were obtained at 3.2× coverage using 168 independent experiments. (E) The results for DNA-20kb-RB-Sim-2 (R = 0.93) were obtained at 4× coverage using 200 independent experiments.

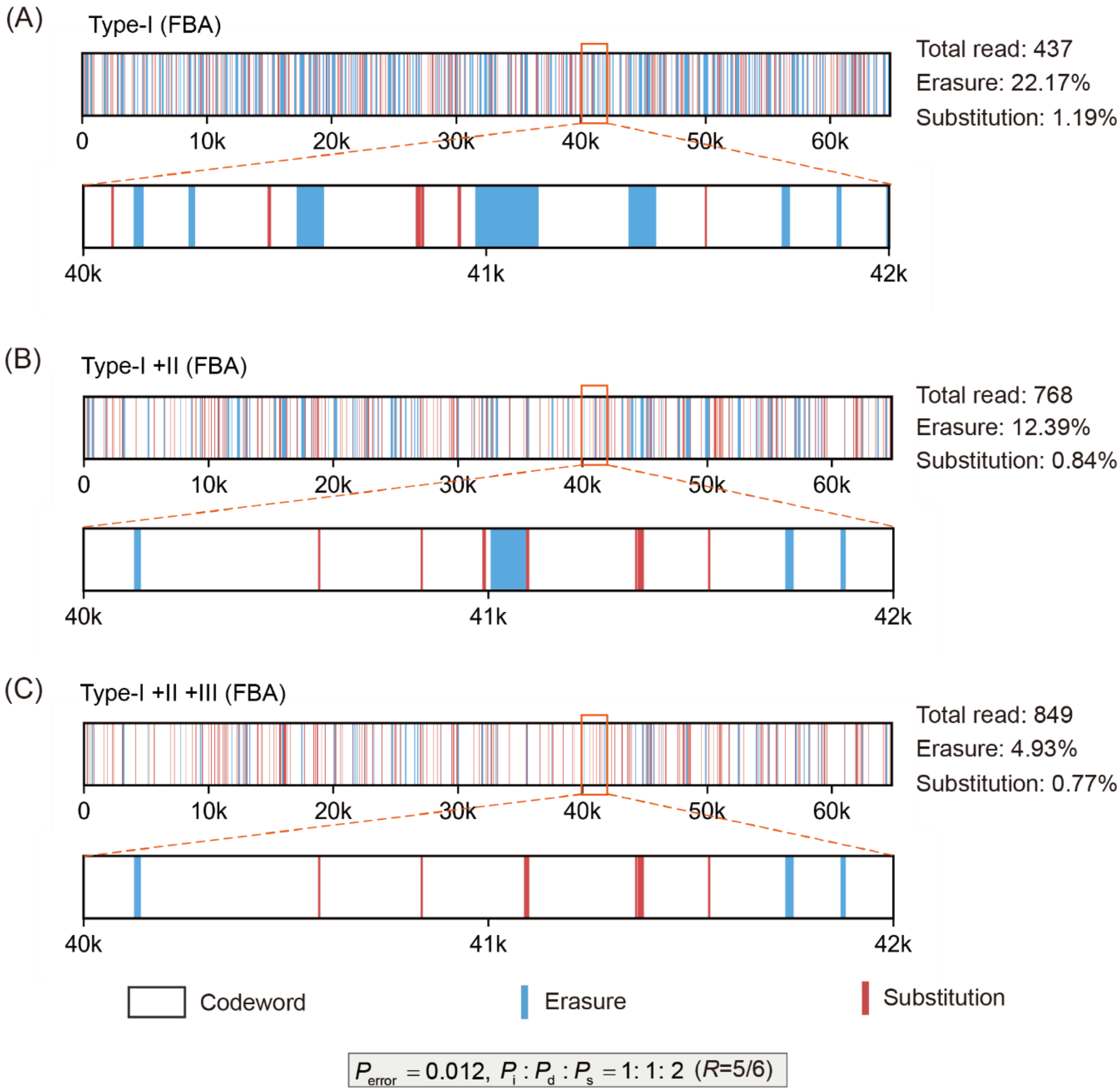

Figure S40 Error distribution across reconstructed codewords using different types of reads. Simulations are based on DNA-40.5kb-MC-Sim-2 (R = 5/6) with a raw error rate of 1.2%. The total number of valid reads and corresponding erasure and substitution rates are shown on the right of each track. Zoom-in views from 40k to 42k highlight local error distributions. Blue and red segments represent erasures and substitutions, respectively. (A) Type-I reads. (B) Type-I + II reads. (C) Type-I + II + III reads.

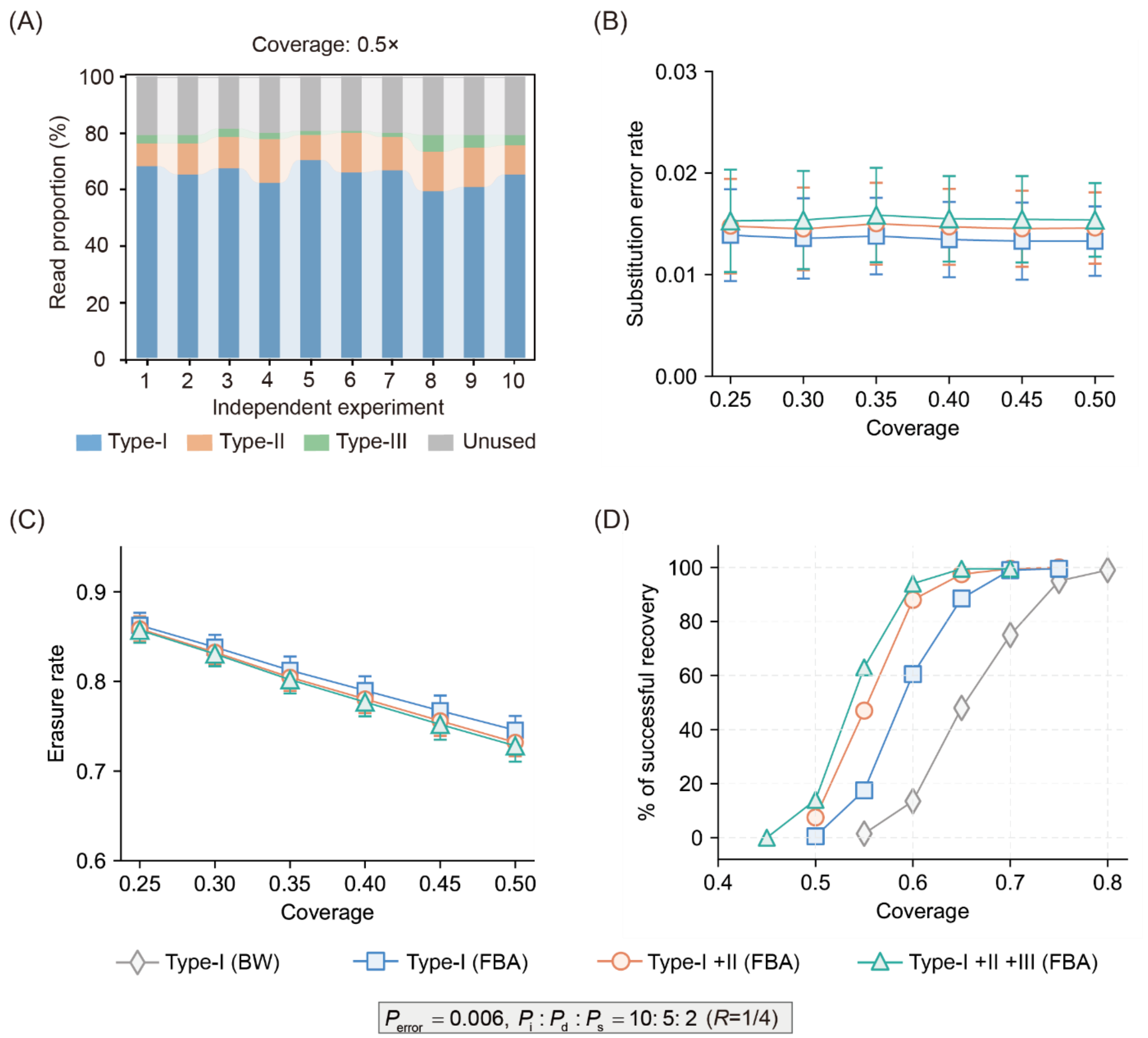

Figure S41 Evaluation of gap-filling scheme on DNA-40.5kb-DR-Sim-1 (*R* = 1/4). (A) Proportions of different types of reads used for data recovery at 0.5× sequencing coverage. (B) Substitution error rates before LDPC decoding using different types of reads. (C) Bit erasure rates before LDPC decoding using different types of reads. Error bars in (B) and (C) represent mean ± SD (standard deviation) calculated from 200 independent simulations. (D) Recovery tests under different sequencing coverages.

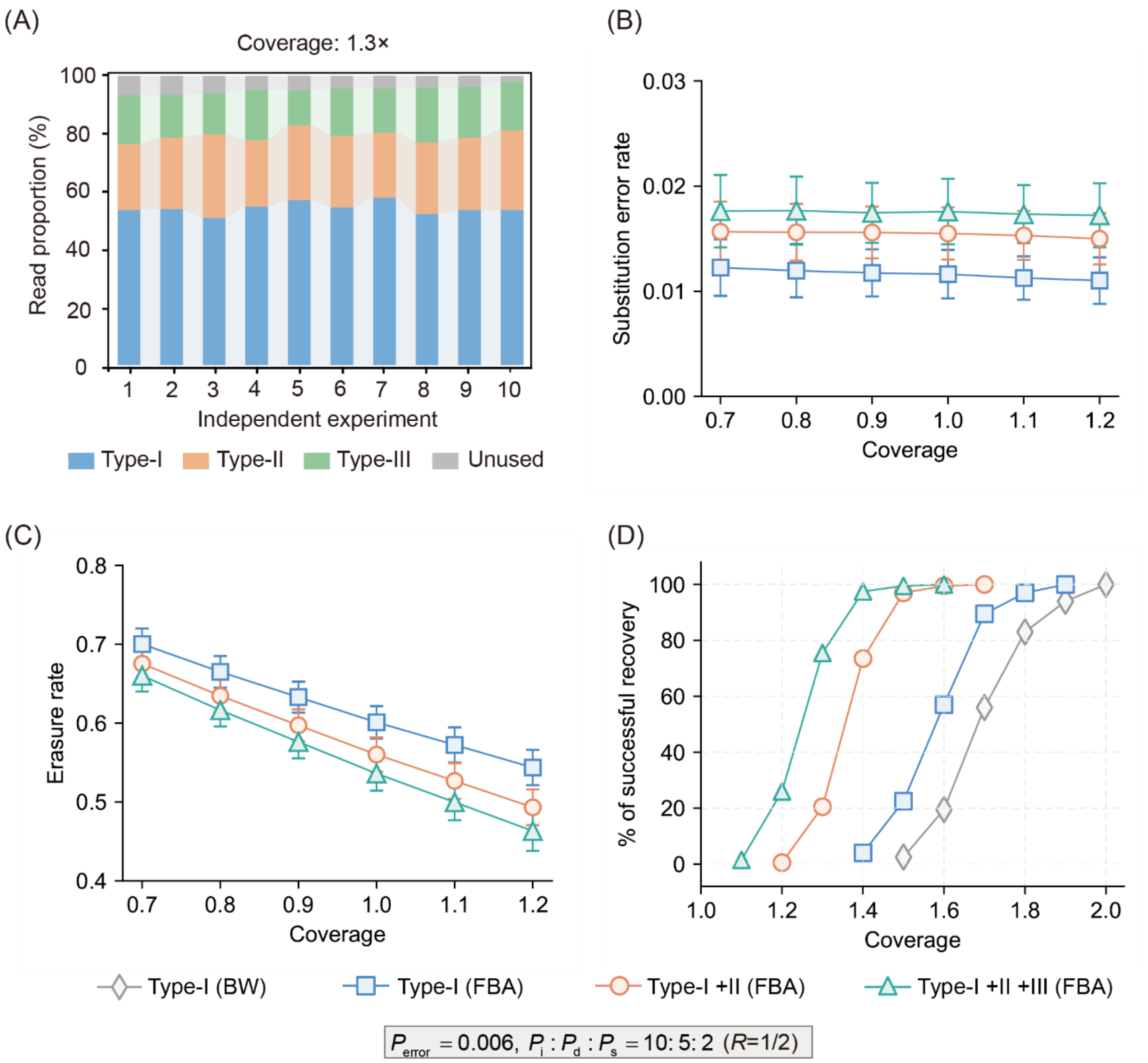

Figure S42 Evaluation of gap-filling scheme on DNA-40.32kb-ER-Sim-1 (*R* = 1/2). (A) Proportions of different types of reads used for data recovery at 1.3× sequencing coverage. (B) Substitution error rates before LDPC decoding using different types of reads. (C) Bit erasure rates before LDPC decoding using different types of reads. Error bars in (B) and (C) represent mean ± SD (standard deviation) calculated from 200 independent simulations. (D) Recovery tests under different sequencing coverages.

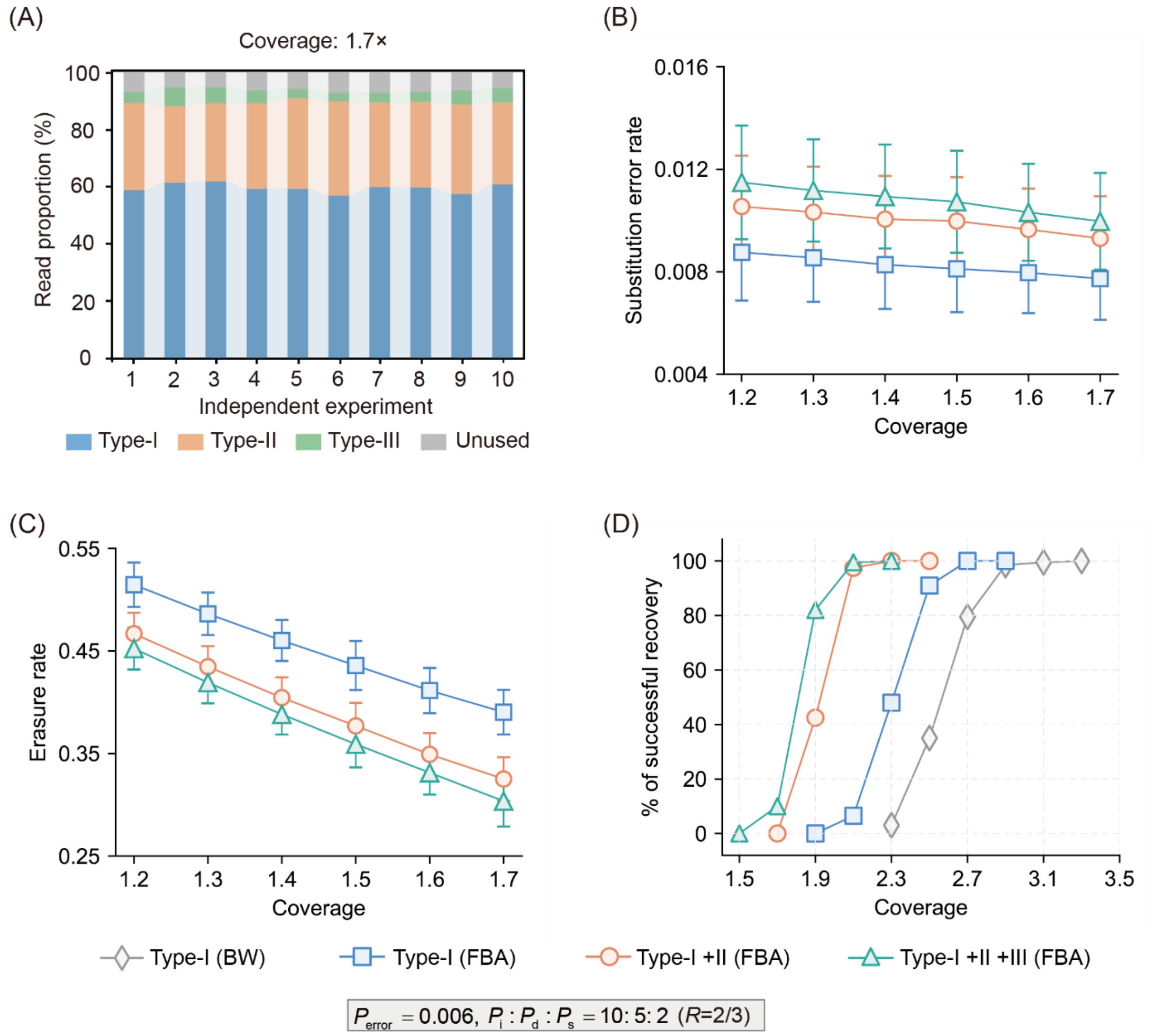

Figure S43 Evaluation of gap-filling scheme on DNA-40.5kb-EM-Sim-1 (*R* = 2/3). (A) Proportions of different types of reads used for data recovery at 1.7× sequencing coverage. (B) Substitution error rates before LDPC decoding using different types of reads. (C) Bit erasure rates before LDPC decoding using different types of reads. Error bars in (B) and (C) represent mean ± SD (standard deviation) calculated from 200 independent simulations. (D) Recovery tests under different sequencing coverages.

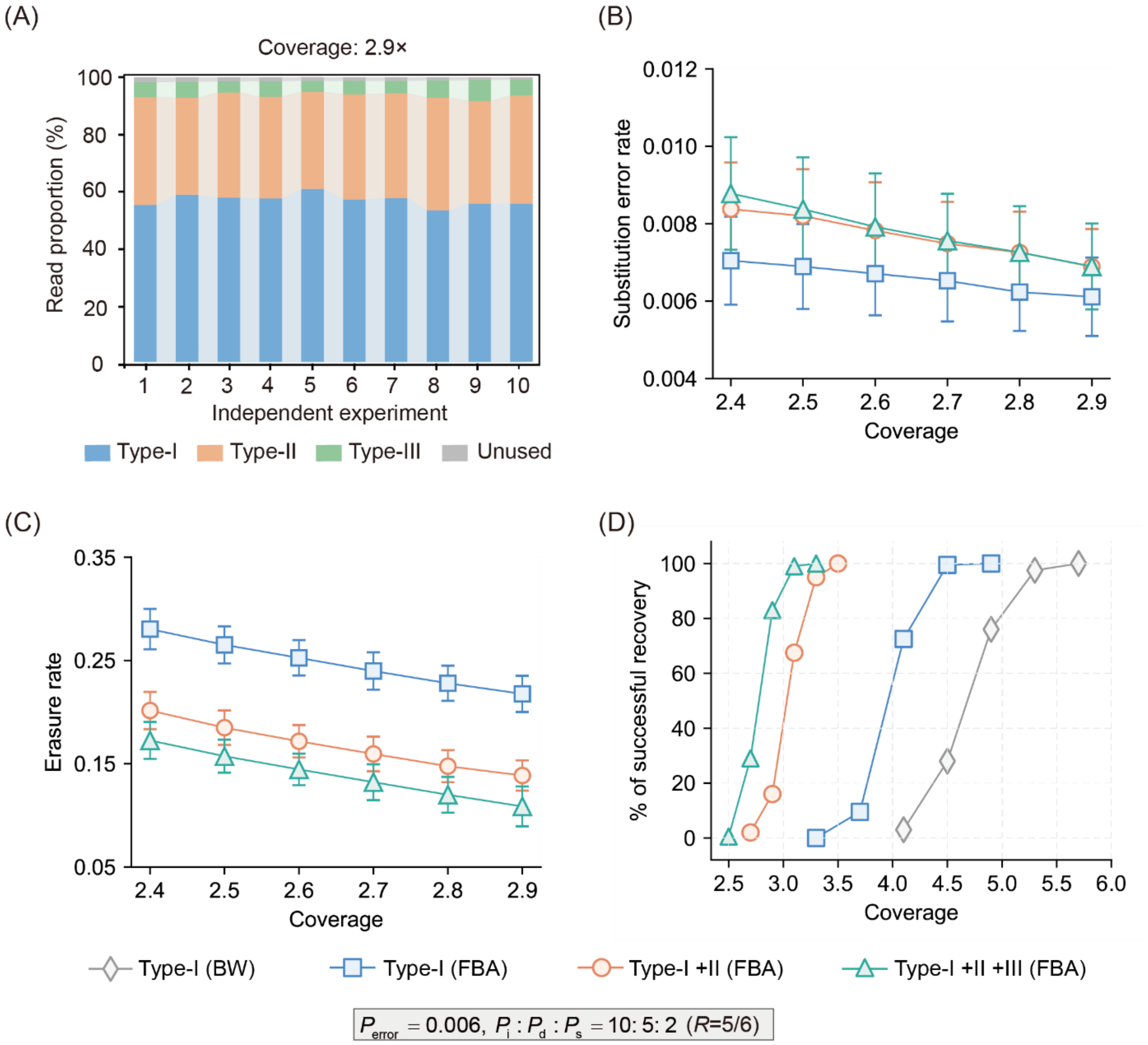

Figure S44 Evaluation of gap-filling scheme on DNA-40.5kb-MC-Sim-1 (*R* = 5/6). (A) Proportions of different types of reads used for data recovery at 2.9× sequencing coverage. (B) Substitution error rates before LDPC decoding using different types of reads. (C) Bit erasure rates before LDPC decoding using different types of reads. Error bars in (B) and (C) represent mean ± SD (standard deviation) calculated from 200 independent simulations. (D) Recovery tests under different sequencing coverages.

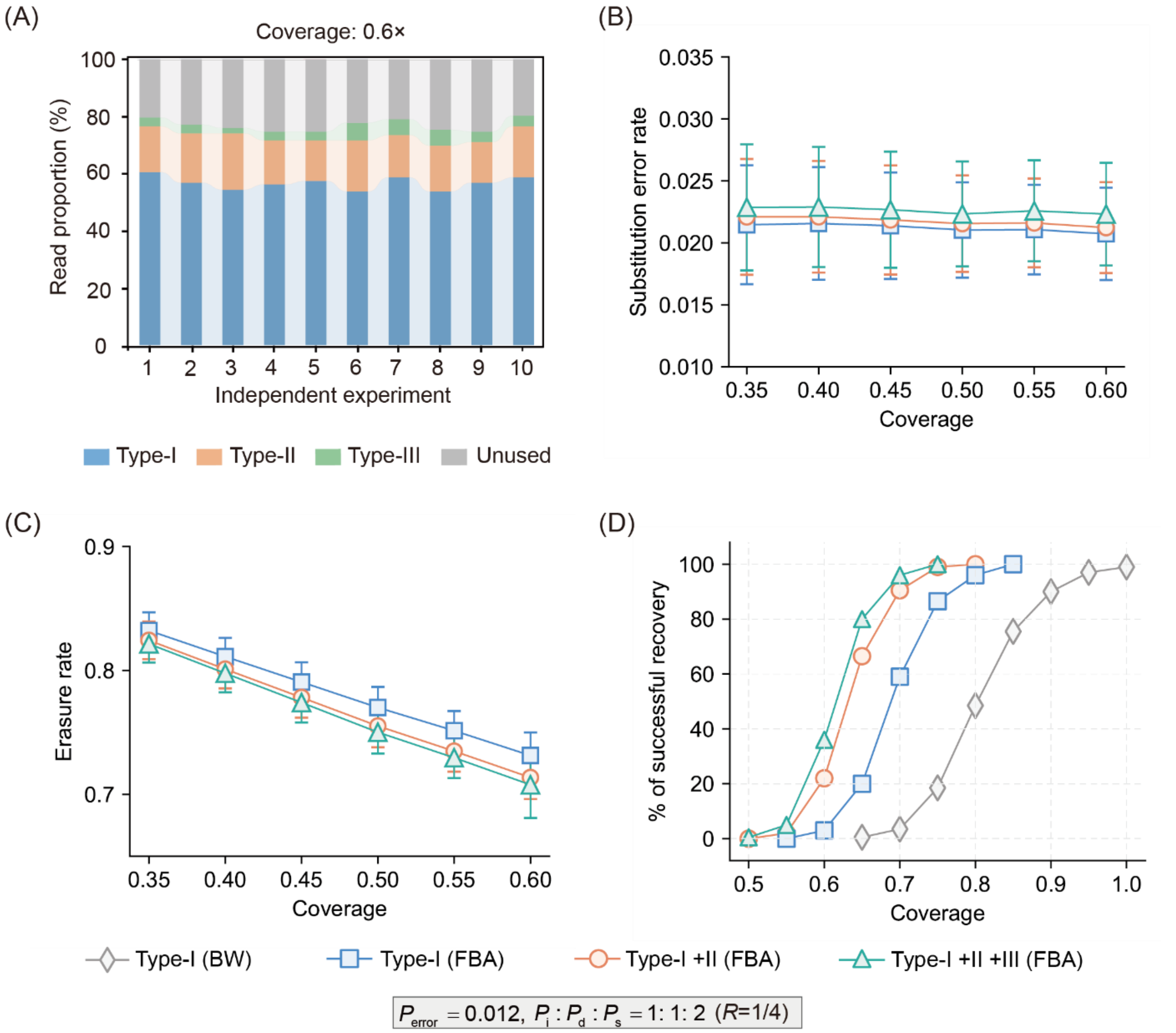

Figure S45 Evaluation of gap-filling scheme on DNA-40.5kb-DR-Sim-2 (*R* = 1/4). (A) Proportions of different types of reads used for data recovery at 0.6× sequencing coverage. (B) Substitution error rates before LDPC decoding using different types of reads. (C) Bit erasure rates before LDPC decoding using different types of reads. Error bars in (B) and (C) represent mean ± SD (standard deviation) calculated from 200 independent simulations. (D) Recovery tests under different sequencing coverages.

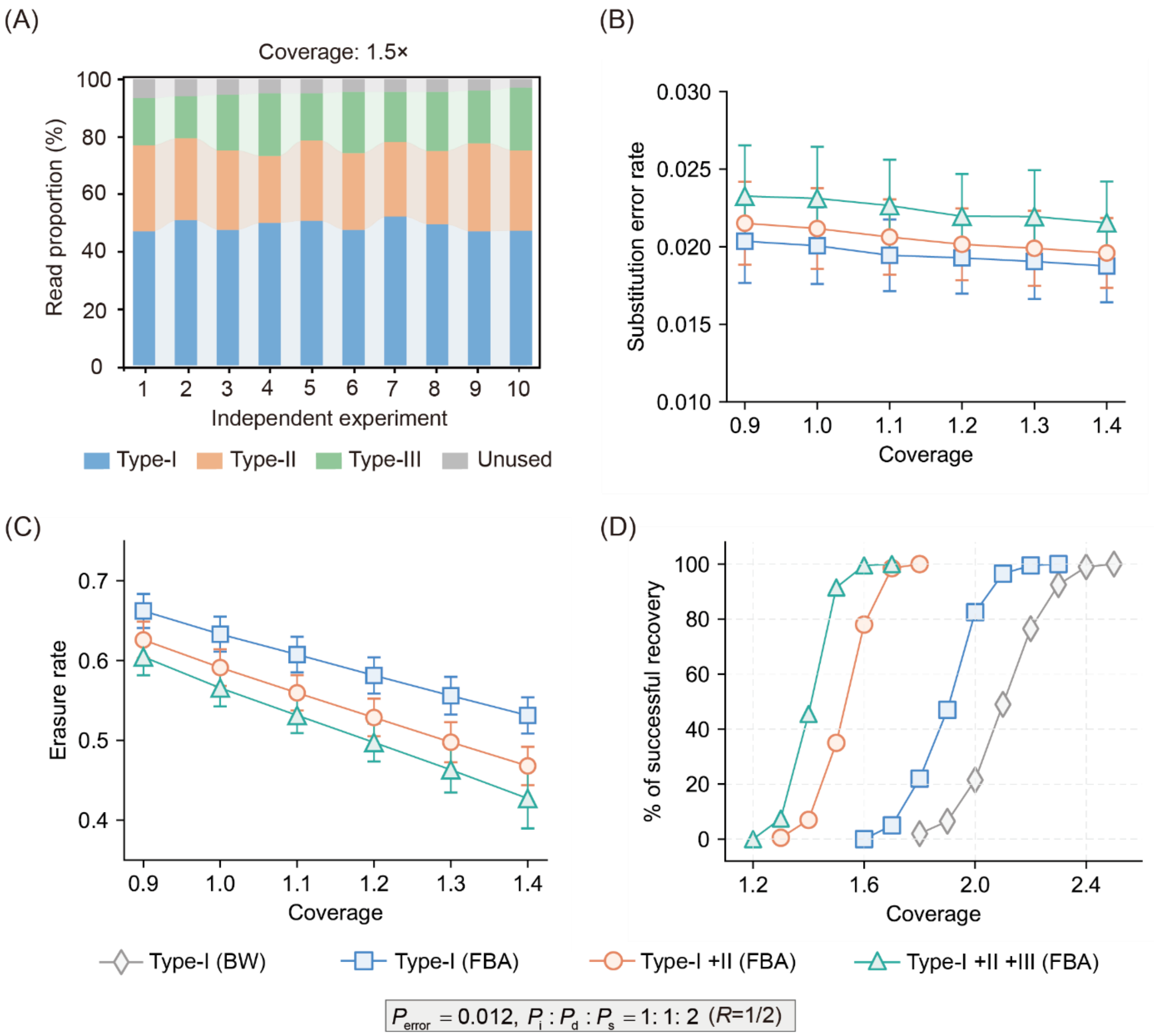

Figure S46 Evaluation of gap-filling scheme on DNA-40.32kb-ER-Sim-2 (*R* = 1/2). (A) Proportions of different types of reads used for data recovery at 1.5× sequencing coverage. (B) Substitution error rates before LDPC decoding using different types of reads. (C) Bit erasure rates before LDPC decoding using different types of reads. Error bars in (B) and (C) represent mean ± SD (standard deviation) calculated from 200 independent simulations. (D) Recovery tests under different sequencing coverages.

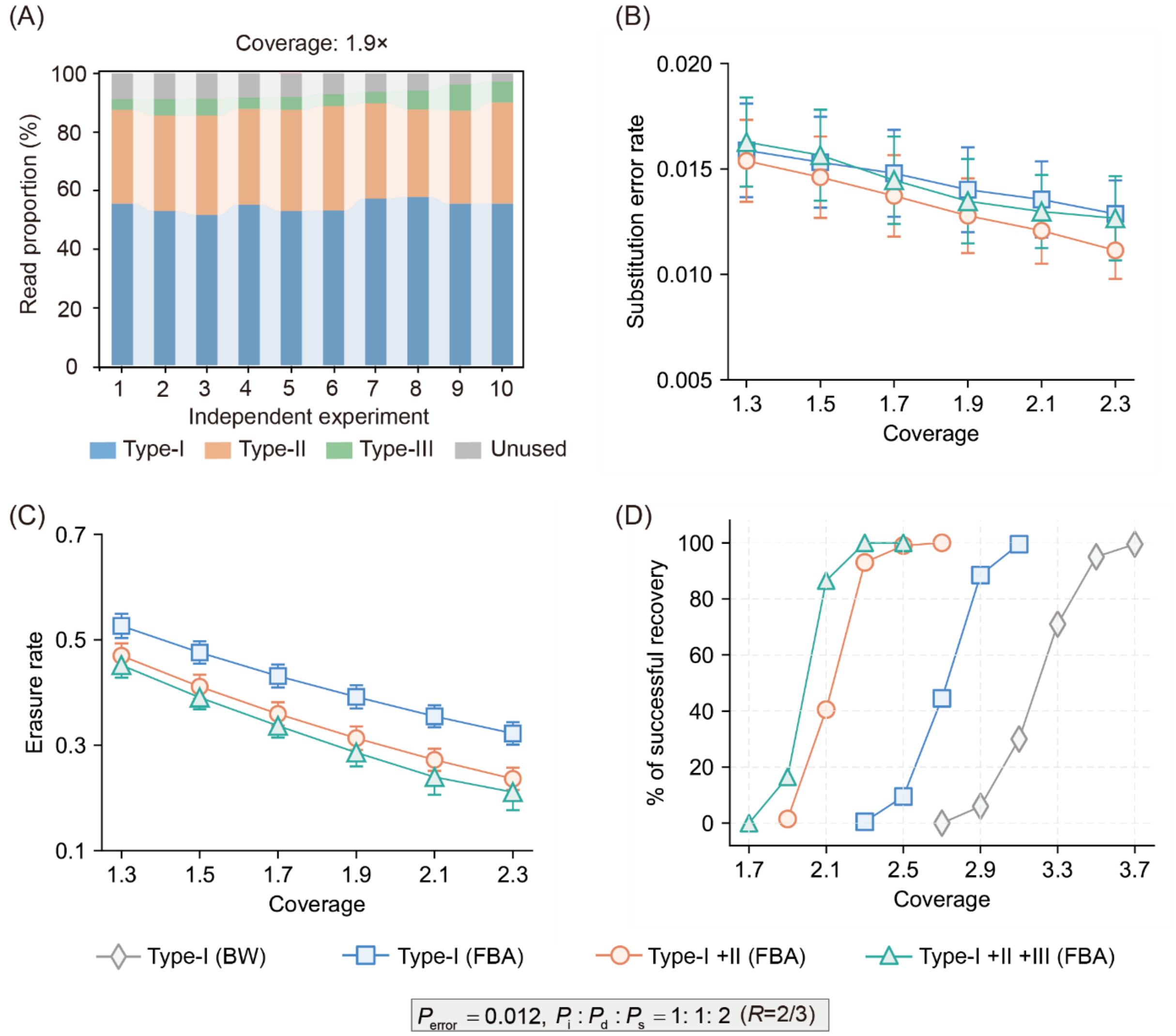

Figure S47 Evaluation of gap-filling scheme on DNA-40.5kb-EM-Sim-2 (*R* = 2/3). (A) Proportions of different types of reads used for data recovery at 1.9× sequencing coverage. (B) Substitution error rates before LDPC decoding using different types of reads. (C) Bit erasure rates before LDPC decoding using different types of reads. Error bars in (B) and (C) represent mean ± SD (standard deviation) calculated from 200 independent simulations. (D) Recovery tests under different sequencing coverages.

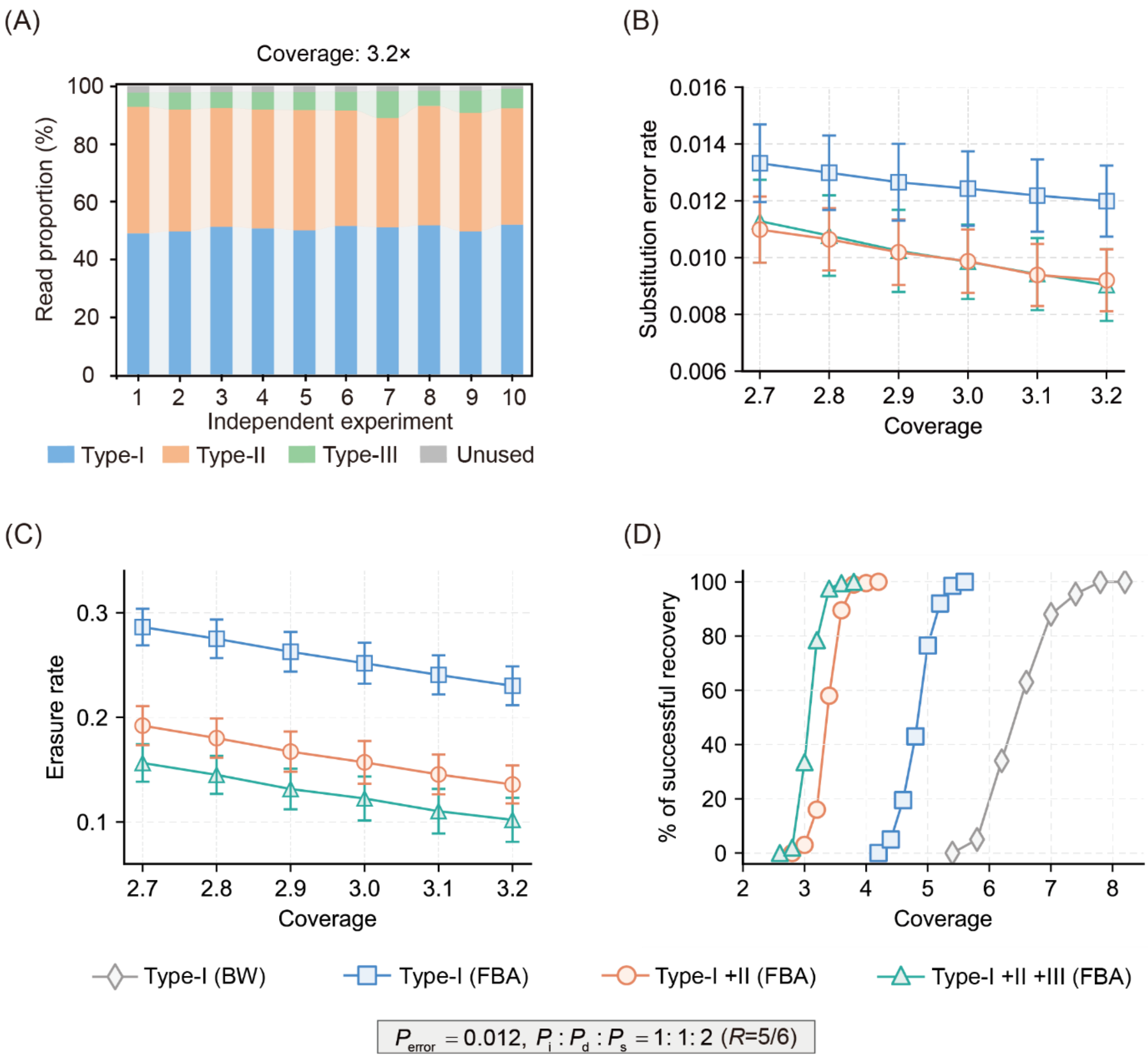

Figure S48 Evaluation of gap-filling scheme on DNA-40.5kb-MC-Sim-2 (*R* = 5/6). (A) Proportions of different types of reads used for data recovery at 3.2× sequencing coverage. (B) Substitution error rates before LDPC decoding using different types of reads. (C) Bit erasure rates before LDPC decoding using different types of reads. Error bars in (B) and (C) represent mean ± SD (standard deviation) calculated from 200 independent simulations. (D) Recovery tests under different sequencing coverages.

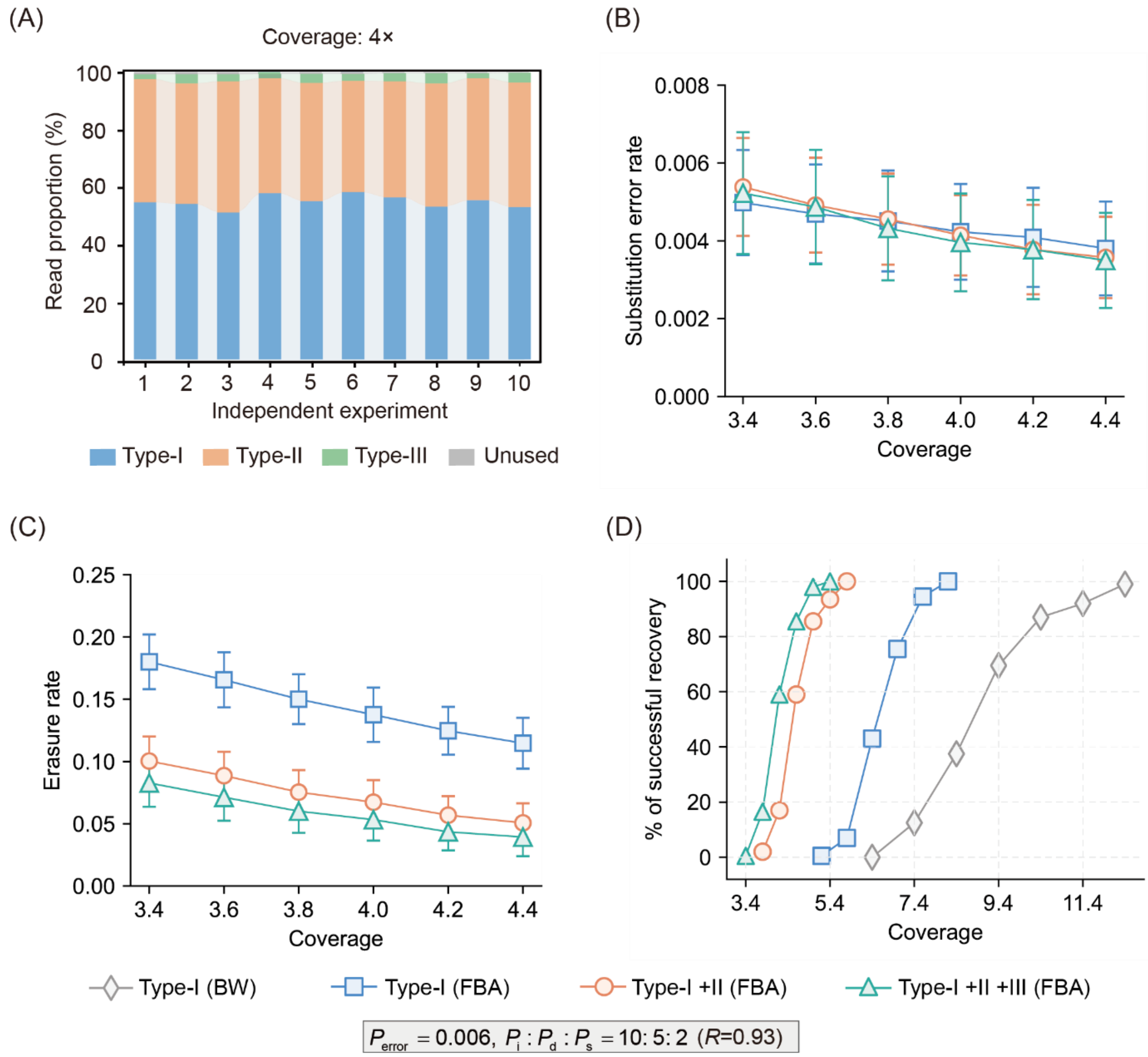

Figure S49 Evaluation of gap-filling scheme on DNA-20kb-RB-Sim-1 (R = 0.93). (A) Proportions of different types of reads used for data recovery at 4× sequencing coverage. (B) Substitution error rates before LDPC decoding using different types of reads. (C) Bit erasure rates before LDPC decoding using different types of reads. Error bars in (B) and (C) represent mean ± SD (standard deviation) calculated from 200 independent simulations. (D) Recovery tests under different sequencing coverages.

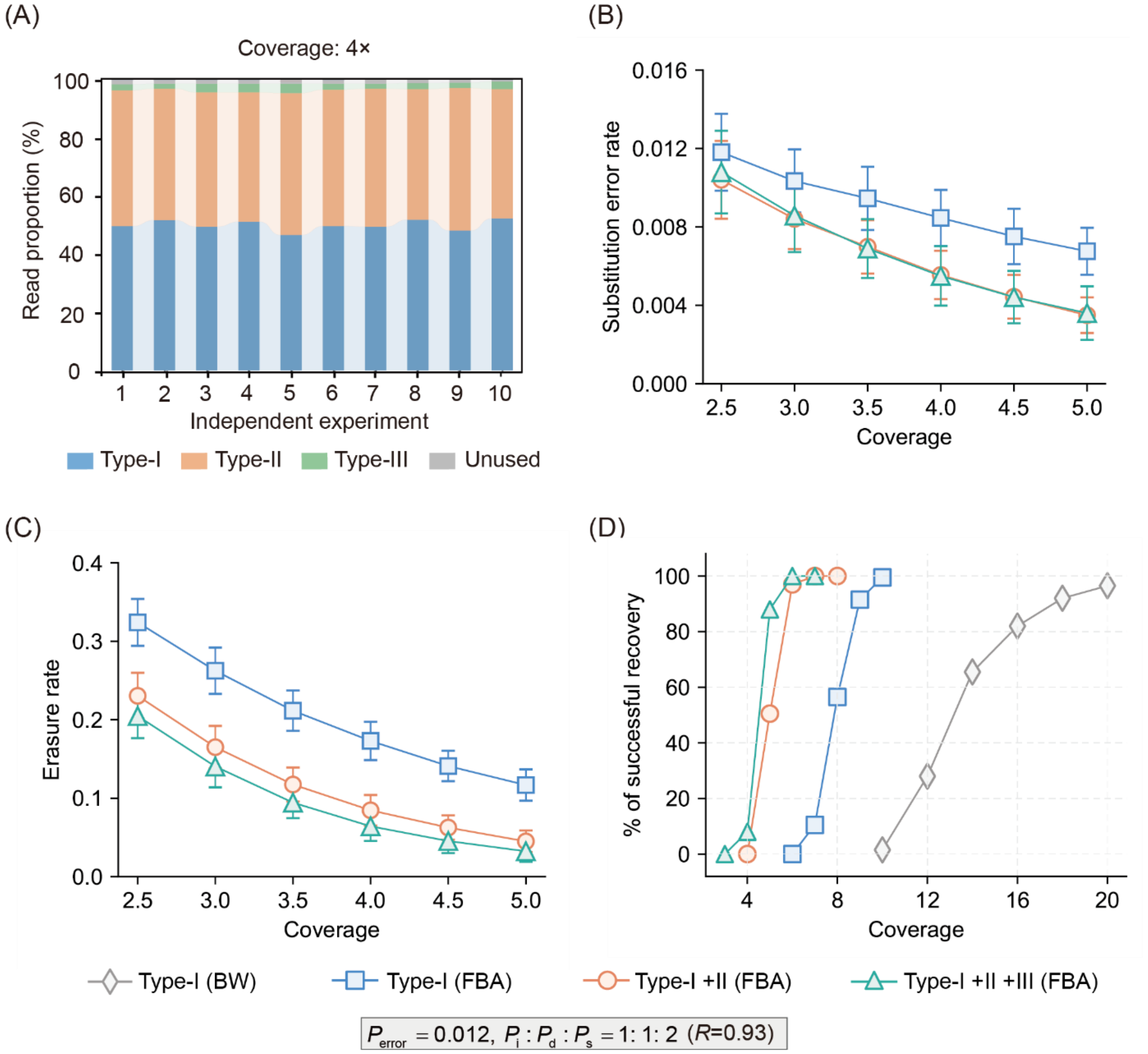

Figure S50 Evaluation of gap-filling scheme on DNA-20kb-RB-Sim-2 (*R* = 0.93). (A) Proportions of different types of reads used for data recovery at 4× sequencing coverage. (B) Substitution error rates before LDPC decoding using different types of reads. (C) Bit erasure rates before LDPC decoding using different types of reads. Error bars in (B) and (C) represent mean ± SD (standard deviation) calculated from 200 independent simulations. (D) Recovery tests under different sequencing coverages.

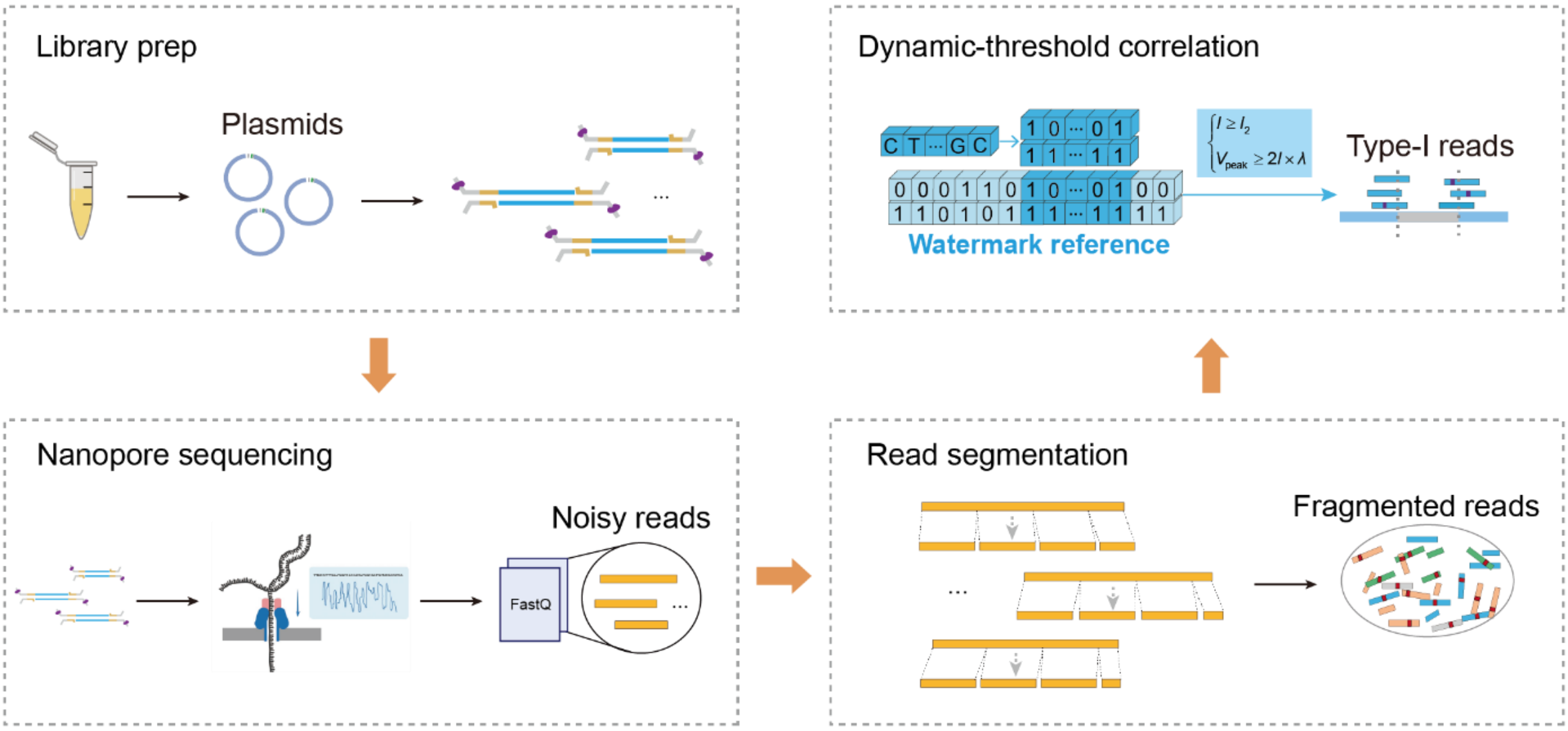

Figure S51 Workflow of nanopore sequencing with read segmentation and dynamic-threshold correlation. Long noisy reads were segmented into predefined lengths, and short residual fragments at read ends were processed with dynamically scaled correlation thresholds to accommodate variable read lengths.

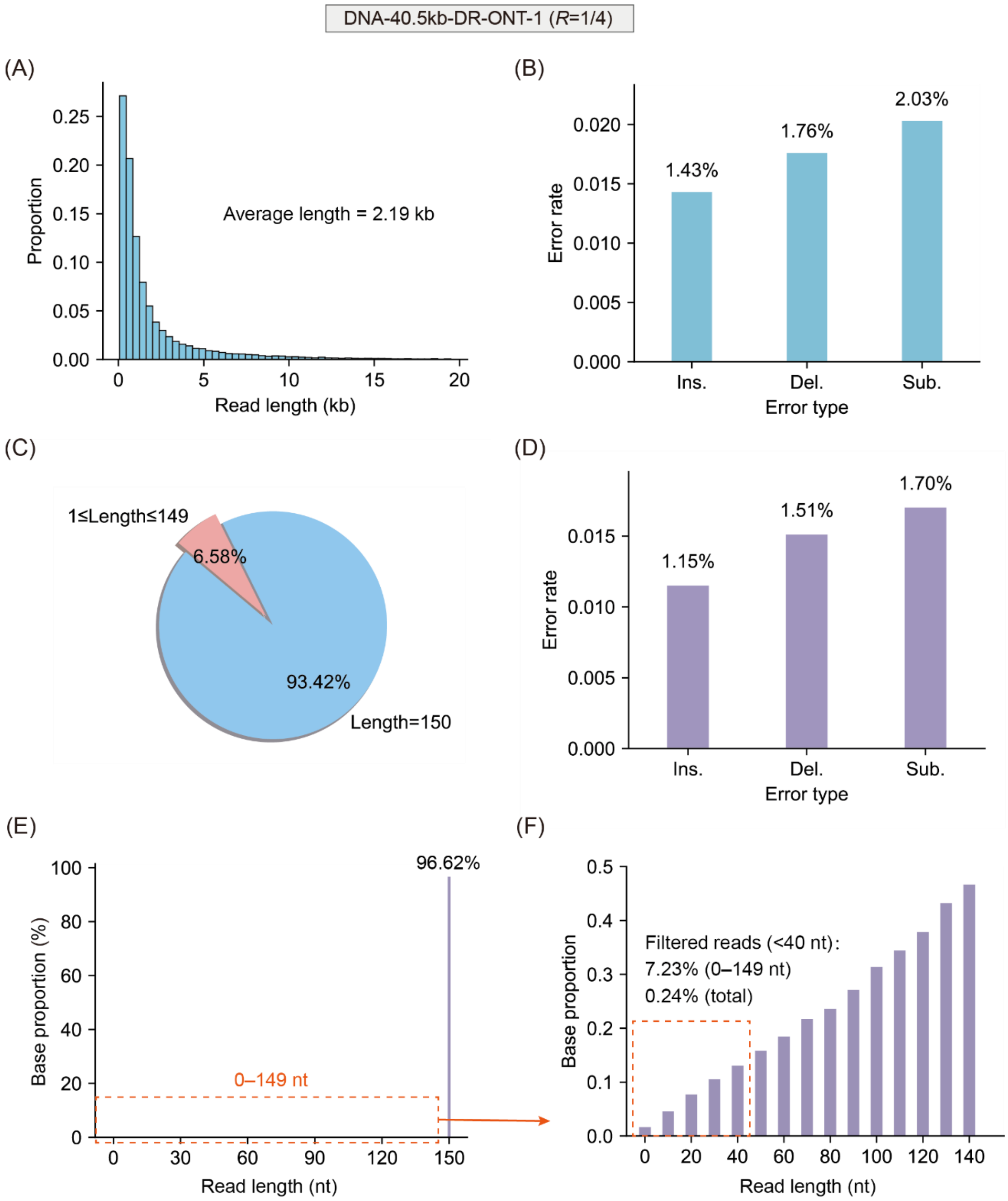

Figure S52 Read length distribution and error rates for DNA-40.5kb-DR-ONT-1 (*R* = 1/4, raw error rate = 5.2%). (A) Read length distribution of raw nanopore reads (a total of 46,000 reads). (B) Base error rates of raw nanopore reads. (C) Composition of reads after segmentation into 150-nt fragments, showing that 150-nt reads constitute 93.42% of the total, whereas shorter reads (1–149 nt) account for 6.58%. (D) Base error rates calculated after segmentation into 150-nt fragments. (E) Base proportion after segmentation into 150-nt fragments, computed using reads with lengths of 1–150 nt. (F) Base-length distribution after segmentation, computed using reads with lengths of 1–149 nt; short reads (<40 nt) accounted for 7.23% of bases, corresponding to 0.24% of the total dataset (including 150-nt fragments).

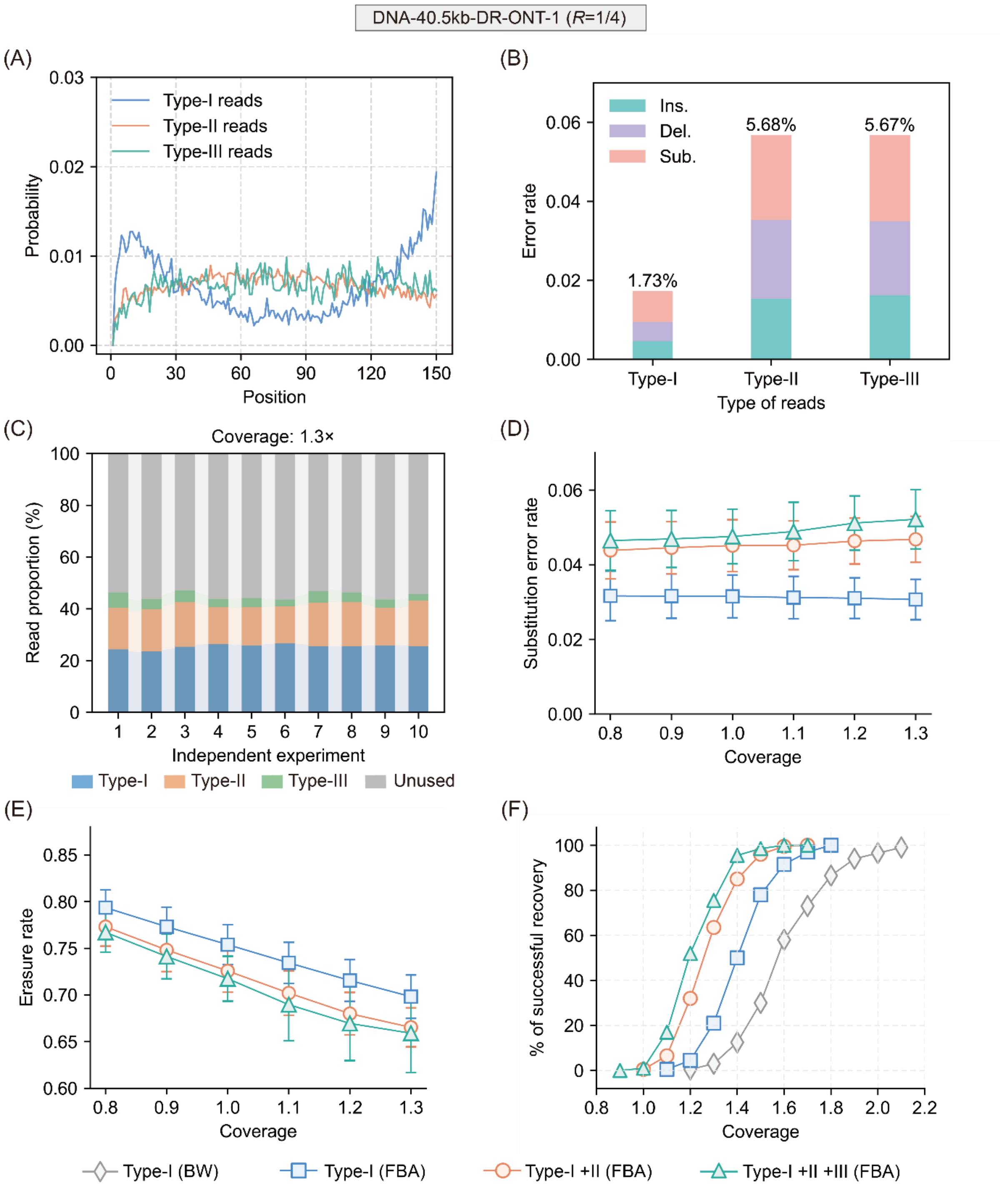

Figure S53 Evaluation of gap-filling scheme on DNA-40.5kb-DR-ONT-1 (*R* = 1/4, raw error rate = 5.2%). (A) Distribution of indel positions across different types of reads in 28 independent experiments. (B) Error profiles across different types of reads. (C) Proportions of different types of reads used for data recovery at 1.3× sequencing coverage. (D) Substitution error rates before LDPC decoding using different types of reads. (E) Bit erasure rates before LDPC decoding using different types of reads. Error bars in (D) and (E) denote standard deviations calculated from 200 independent simulations. (F) Recovery tests under different sequencing coverages.

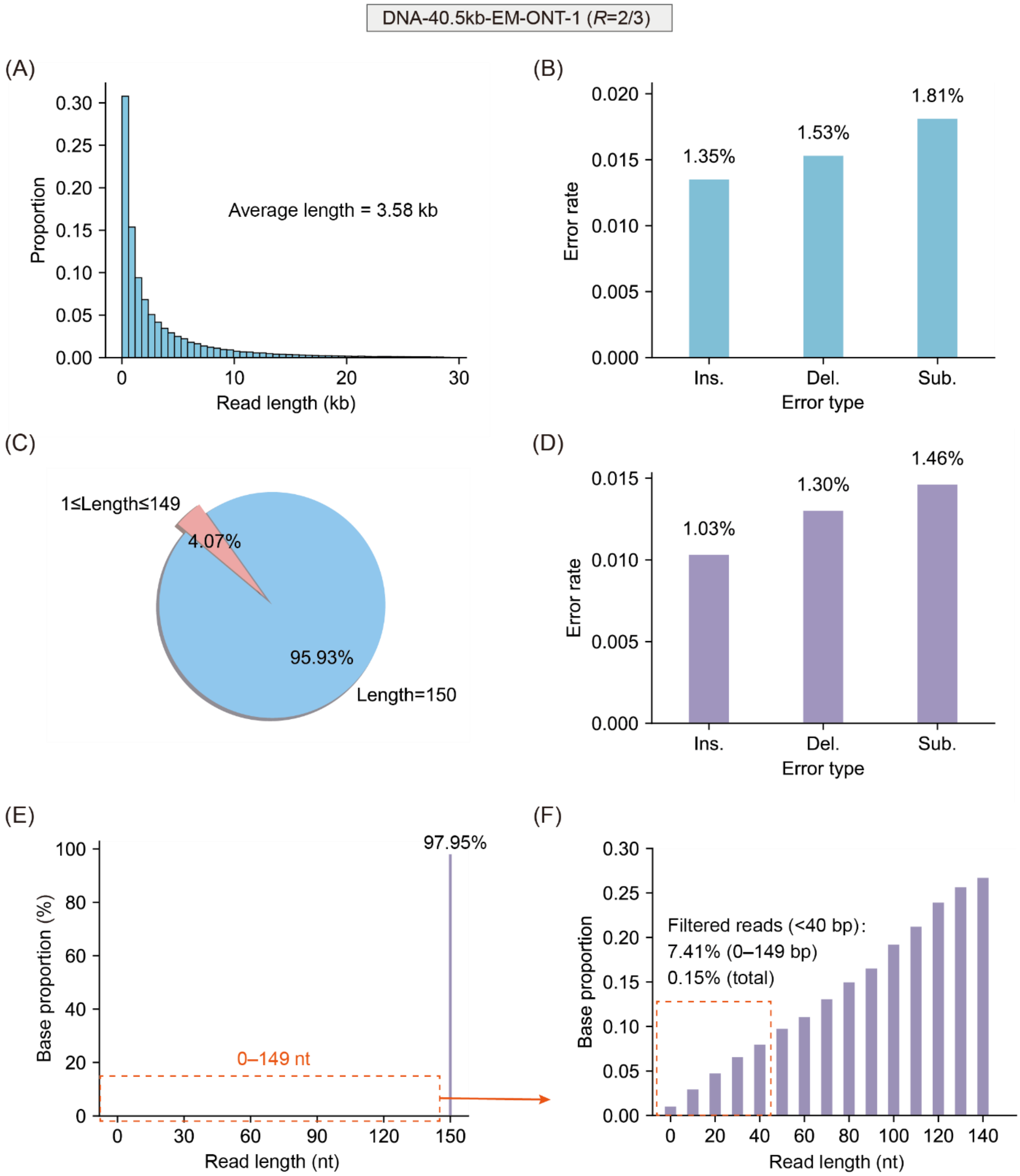

Figure S54 Read length distribution and error rates for DNA-40.5kb-EM-ONT-1 (*R* = 2/3, raw error rate = 4.7%). (A) Read length distribution of raw nanopore reads (a total of 46,000 reads). (B) Base error rates of raw nanopore reads. (C) Composition of reads after segmentation into 150-nt fragments, where 150-nt reads accounted for 95.93% of the total and shorter reads (1–149 nt) accounted for 4.07%. (D) Base error rates calculated after segmentation into 150-nt fragments. (E) Base proportion after segmentation into 150-nt fragments, computed using reads with lengths of 1–150 nt. (F) Base-length distribution after segmentation, computed using reads with lengths of 1–149 nt; short reads (<40 nt) accounted for 7.41% of bases, corresponding to 0.15% of the total dataset (including 150-nt fragments).

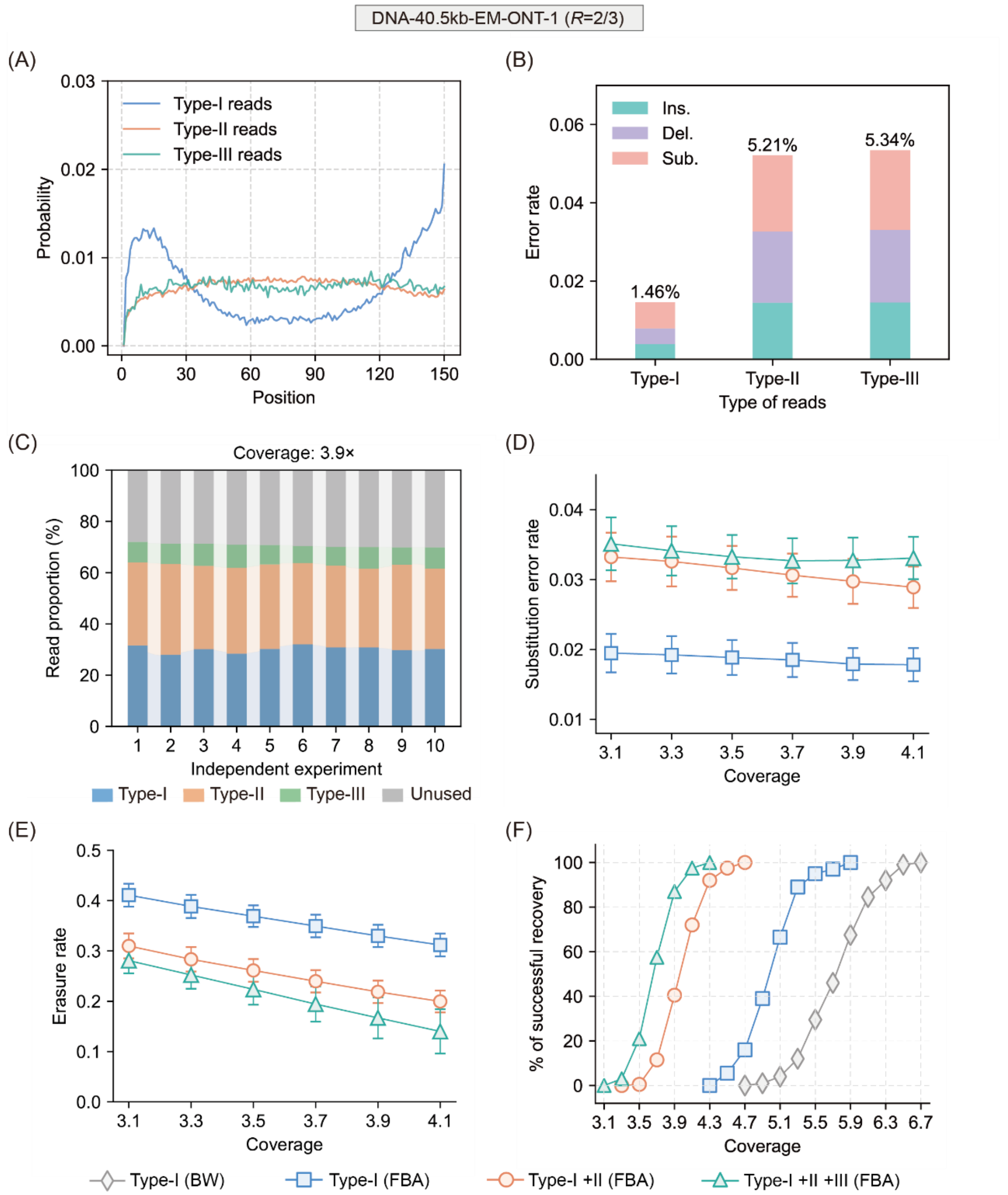

Figure S55 Evaluation of gap-filling scheme on DNA-40.5kb-EM-ONT-1 (*R* = 2/3, raw error rate = 4.7%). (A) Distribution of indel positions across different types of reads in 120 independent experiments. (B) Error profiles across different types of reads. (C) Proportions of different types of reads used for data recovery at 3.9× sequencing coverage. (D) Substitution error rates before LDPC decoding using different types of reads. (E) Bit erasure rates before LDPC decoding using different types of reads. Error bars in (D) and (E) denote standard deviations calculated from 200 independent simulations. (F) Recovery tests under different sequencing coverages.

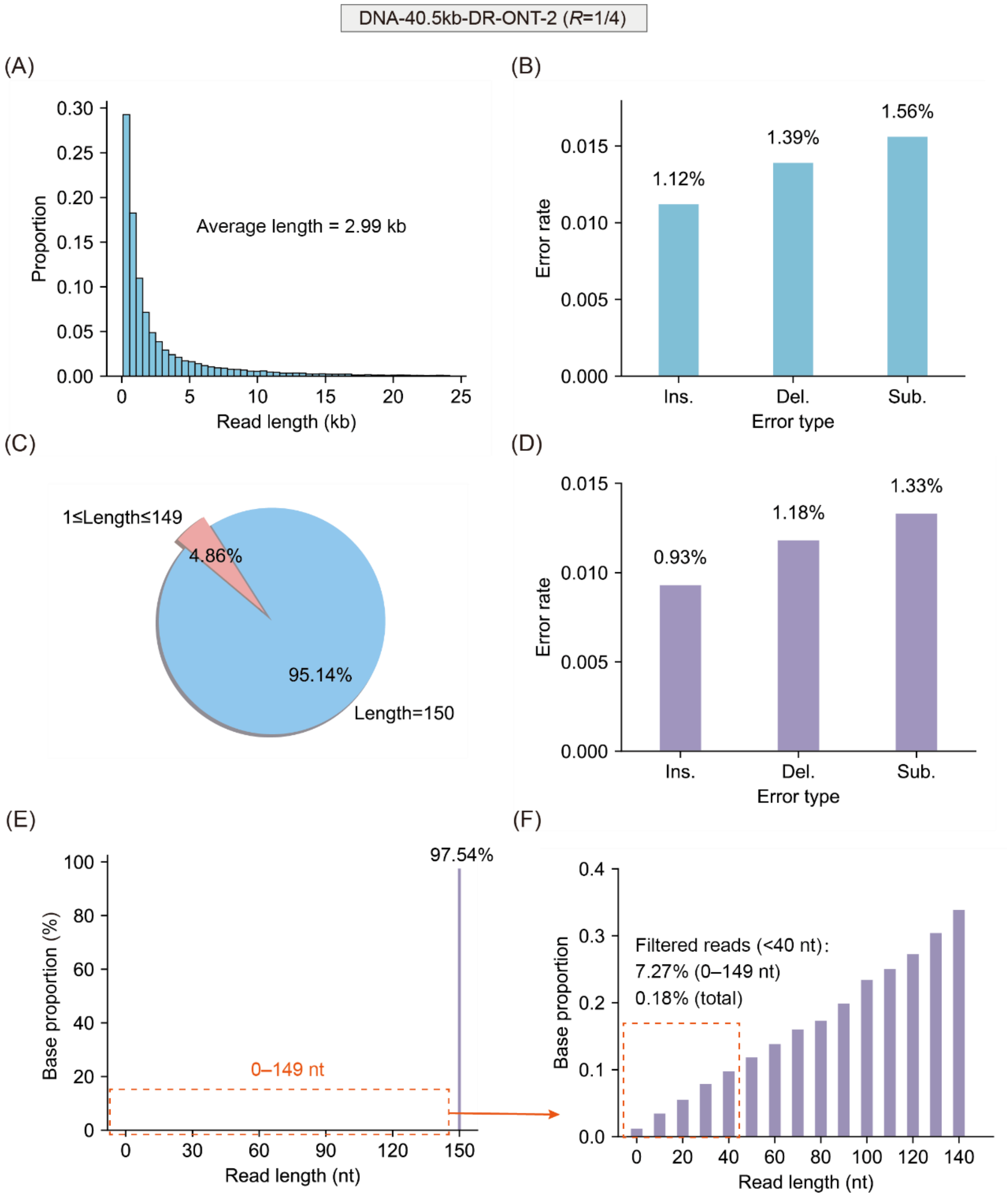

Figure S56 Read length distribution and error rates for DNA-40.5kb-DR-ONT-2 (*R* = 1/4, raw error rate = 4.1%). (A) Read length distribution of raw nanopore reads (a total of 36,000 reads). (B) Base error rates of raw nanopore reads. (C) Composition of reads after segmentation into 150-nt fragments, showing that 150-nt reads constitute 95.14% of the total, whereas shorter reads (1–149 nt) account for 4.86%. (D) Base error rates calculated after segmentation into 150-nt fragments. (E) Base proportion after segmentation into 150-nt fragments, computed using reads with lengths of 1–150 nt. (F) Base-length distribution after segmentation, computed using reads with lengths of 1–149 nt; short reads (<40 nt) accounted for 7.27% of bases, corresponding to 0.18% of the total dataset (including 150-nt fragments).

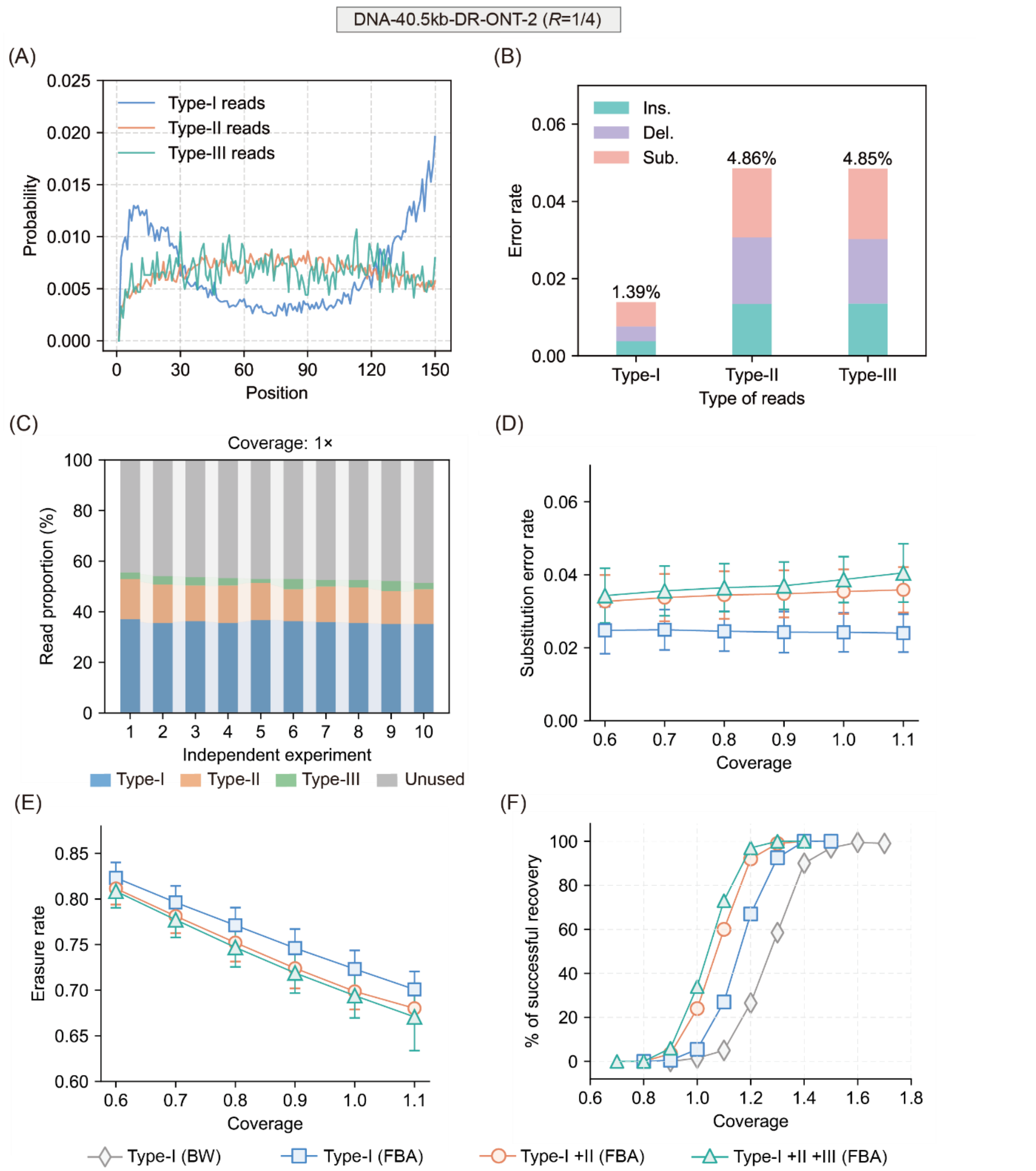

Figure S57 Evaluation of gap-filling scheme on DNA-40.5kb-DR-ONT-2 (*R* = 1/4, raw error rate = 4.1%). (A) Distribution of indel positions across different types of reads in 152 independent experiments. (B) Error profiles across different types of reads. (C) Proportions of different types of reads used for data recovery at 1× sequencing coverage. (D) Substitution error rates before LDPC decoding using different types of reads. (E) Bit erasure rates before LDPC decoding using different types of reads. Error bars in (D) and (E) denote standard deviations calculated from 200 independent simulations. (F) Recovery tests under different sequencing coverages.

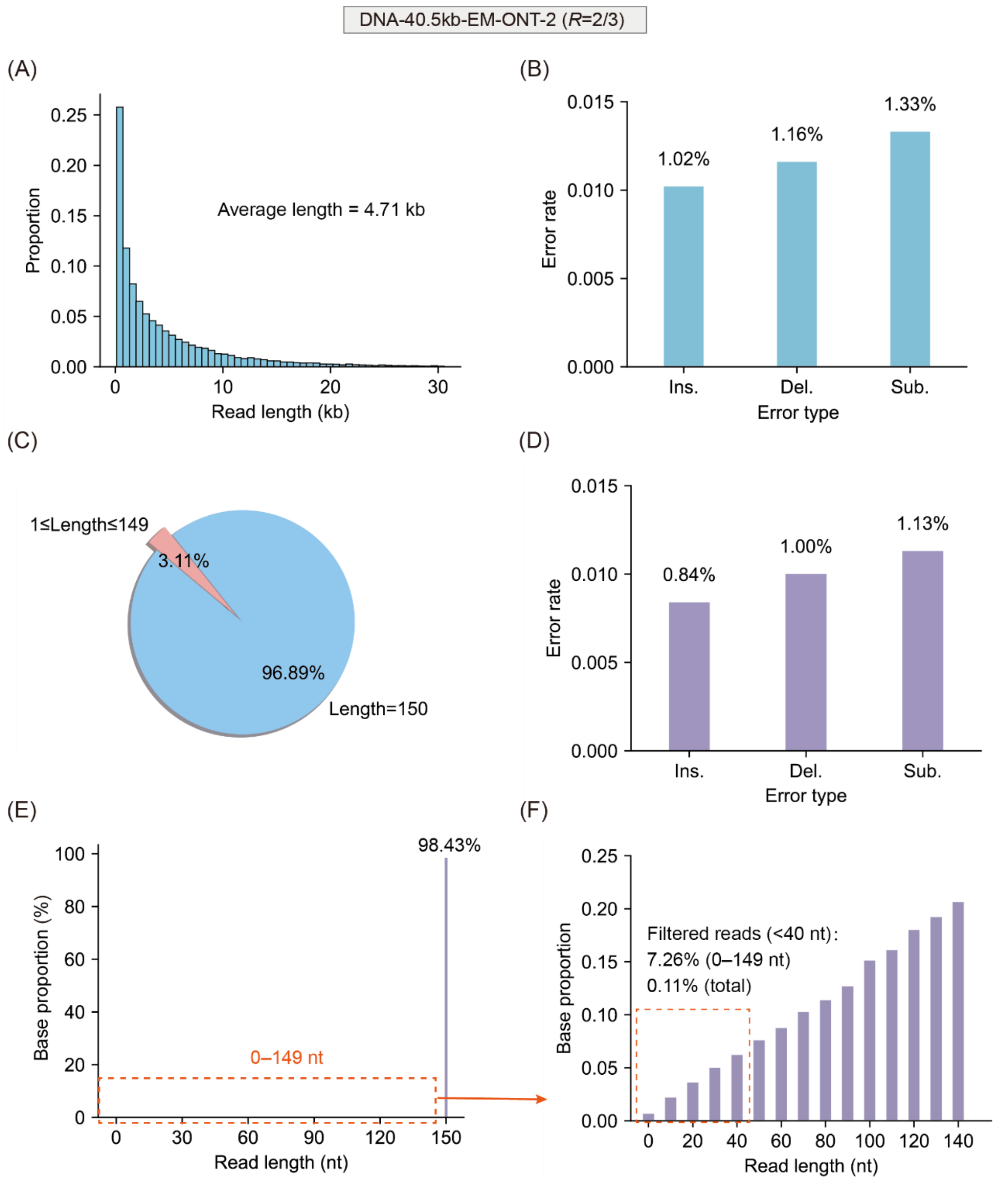

Figure S58 Read length distribution and error rates for DNA-40.5kb-EM-ONT-2 (*R* = 2/3, raw error rate = 3.5%). (A) Read length distribution of raw nanopore reads (a total of 36,000 reads). (B) Base error rates of raw nanopore reads. (C) Composition of reads after segmentation into 150-nt fragments, where 150-nt reads accounted for 96.89% of the total and shorter reads (1–149 nt) accounted for 3.11%. (D) Base error rates calculated after segmentation into 150-nt fragments. (E) Base proportion after segmentation into 150-nt fragments, computed using reads with lengths of 1–150 nt. (F) Base-length distribution after segmentation, computed using reads with lengths of 1–149 nt; short reads (<40 bp) accounted for 7.26% of bases, corresponding to 0.11% of the total dataset (including 150-nt fragments).

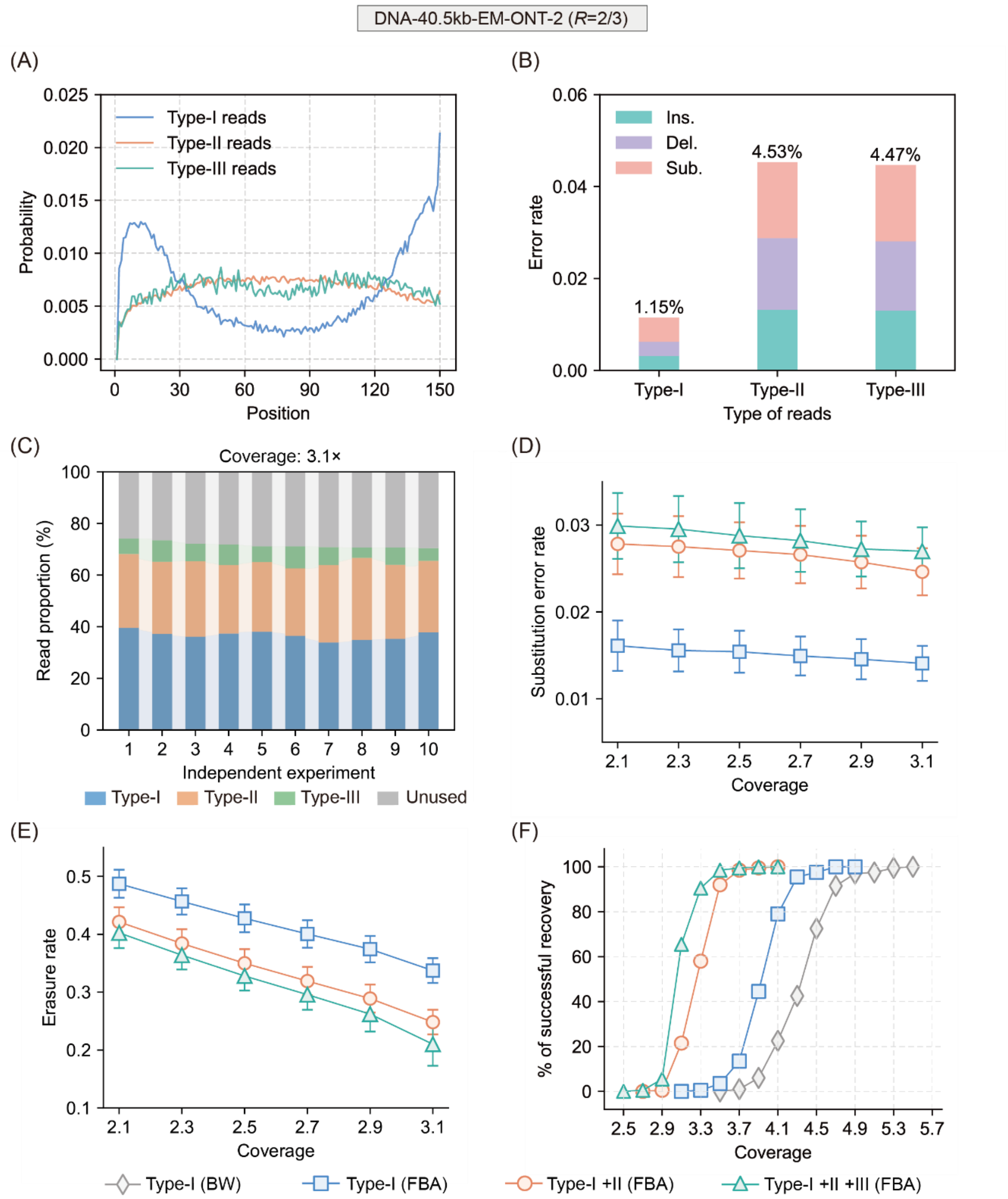

Figure S59 Evaluation of gap-filling scheme on DNA-40.5kb-EM-ONT-2 (*R* = 2/3, raw error rate = 3.5%). (A) Distribution of indel positions across different types of reads in 157 independent experiments. (B) Error profiles across different types of reads. (C) Proportions of different types of reads used for data recovery at 3.1× sequencing coverage. (D) Substitution error rates before LDPC decoding using different types of reads. (E) Bit erasure rates before LDPC decoding using different types of reads. Error bars in (D) and (E) denote standard deviations calculated from 200 independent simulations. (F) Recovery tests under different sequencing coverages.

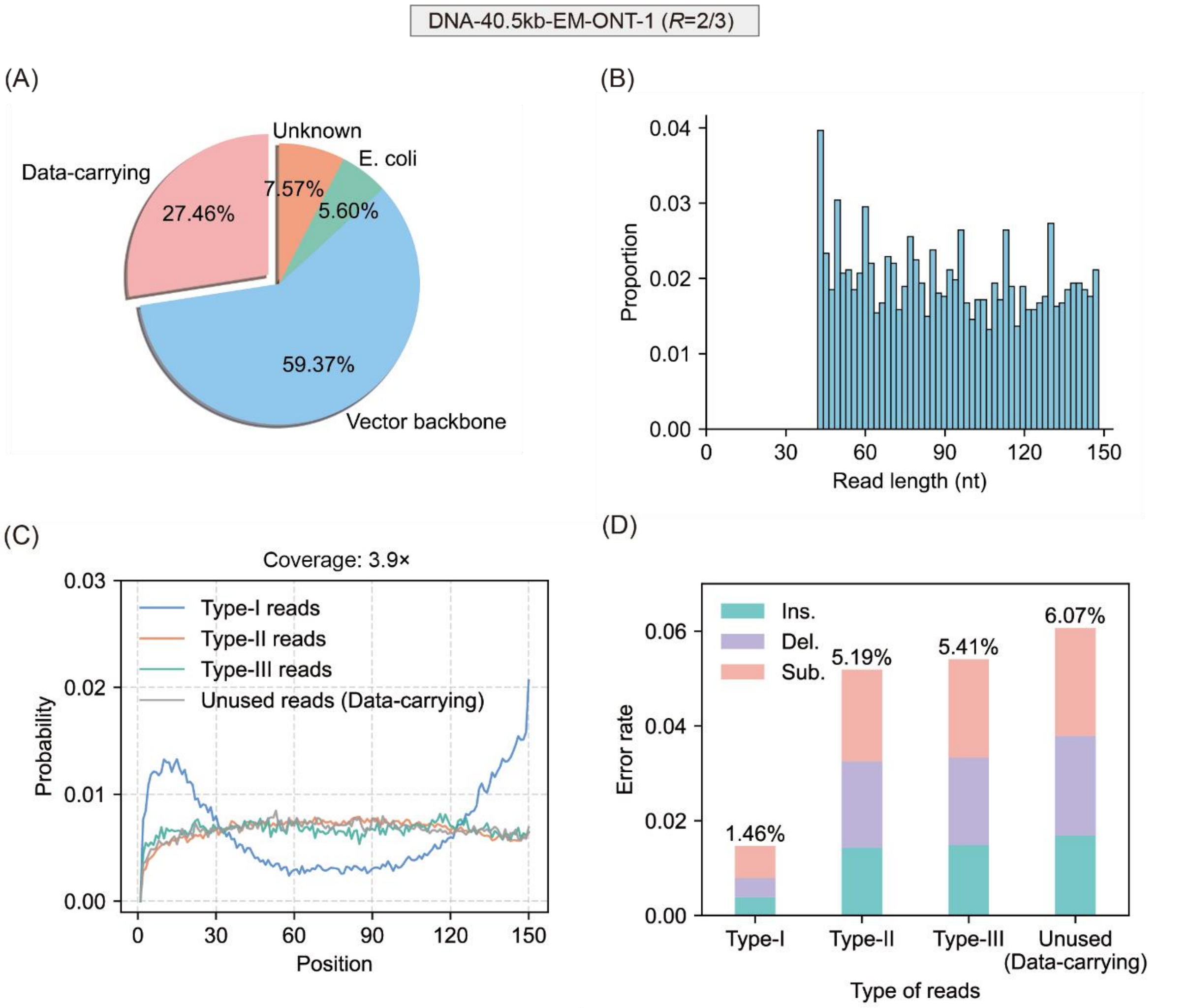

Figure S60 Read length distribution and error profile of unused reads from DNA-40.5kb-EM-ONT-1 (*R* = 2/3). (A) Read composition of unused reads. (B) Read length distribution of unused reads. (C) Distribution of indel error positions across types of reads. The results were obtained at 3.9× coverage using 119 independent experiments. (D) Error profiles showing insertion, deletion, and substitution rates across different types of reads.

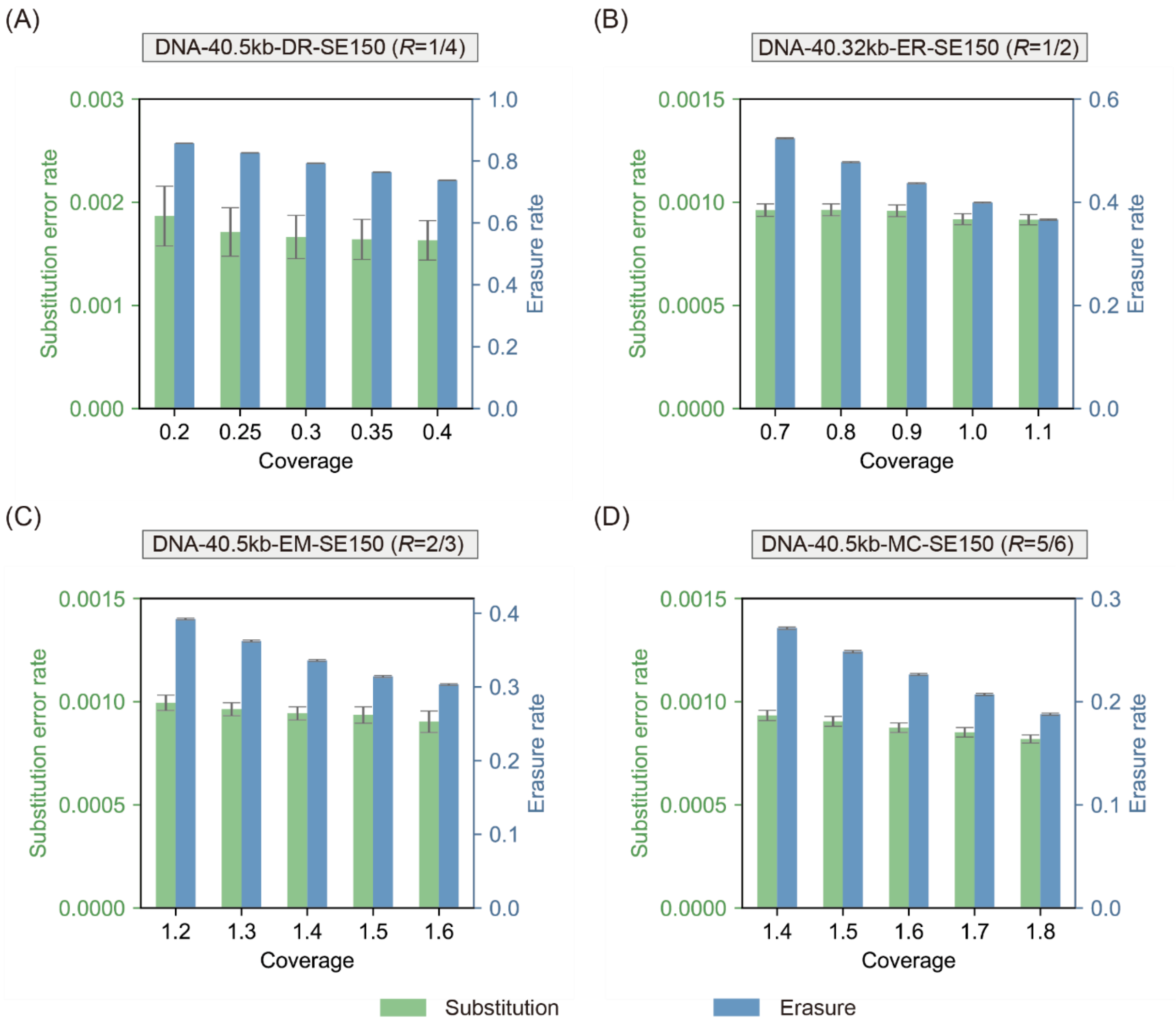

Figure S61 Base erasure rate and base substitution error rate of the scaffold reference (real Illumina data). Blue and green bars represent base erasure and substitution rates, respectively. Error bars denote standard deviations from 200 independent simulations. (A) DNA-40.5kb-DR-SE150 (R = 1/4) with a raw error rate of 0.18%. (B) DNA-40.32 kb-ER-SE150 (R = 1/2) with a raw error rate of 0.15%. (C) DNA-40.5kb-EM-SE150 (R = 2/3) with a raw error rate of 0.20%. (D) DNA-40.5kb-MC-SE150 (R = 5/6) with a raw error rate of 0.14%.

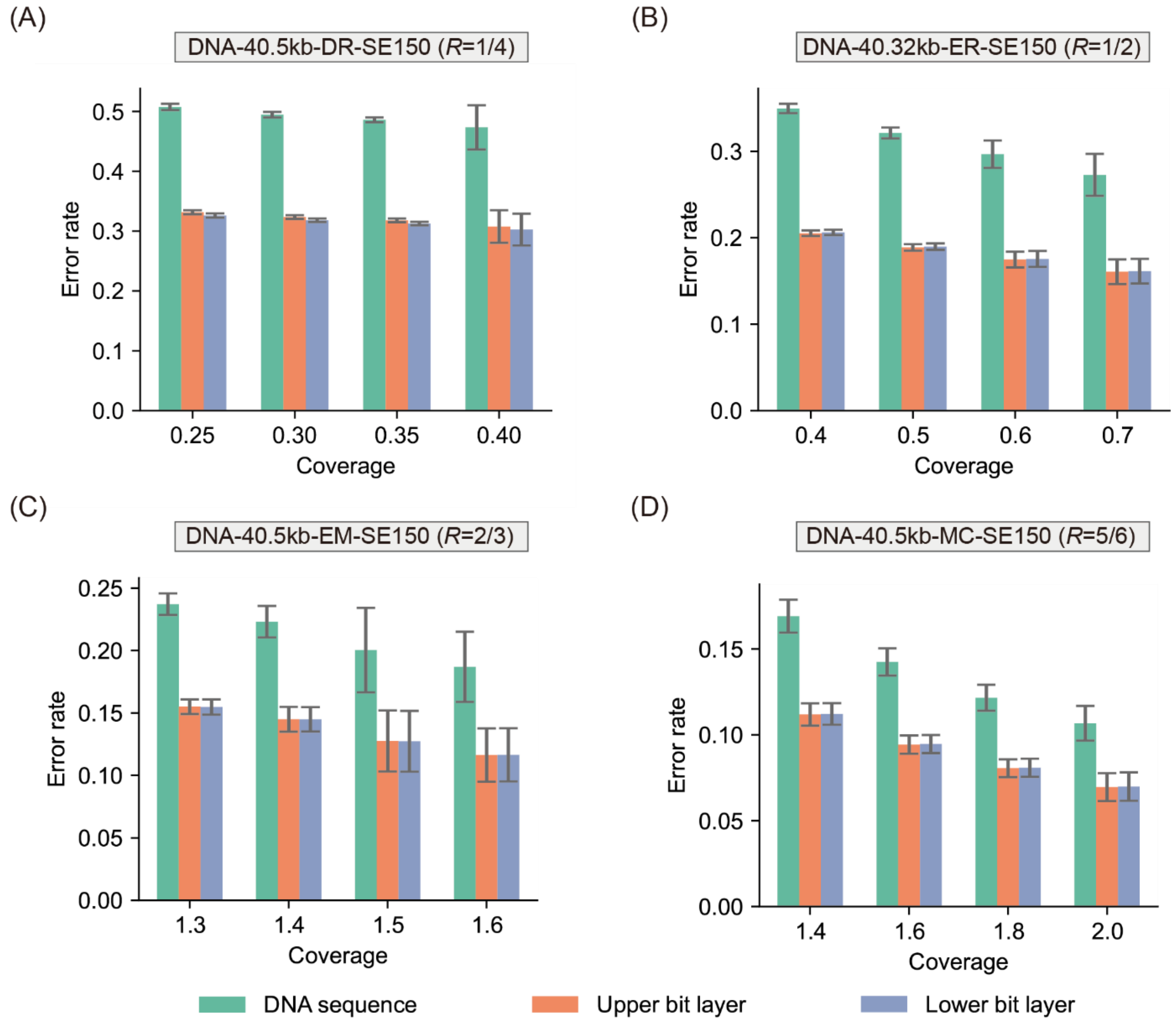

Figure S62 Error rates of the regenerative reference under varying sequencing coverages (real Illumina data). The error rates were evaluated separately for the DNA sequence layer, the upper bit layer, and the lower bit layer under various code rates across 200 trials. (A) DNA-40.5kb-DR-SE150 (R = 1/4) with a raw error rate of 0.18%. (B) DNA-40.32 kb-ER-SE150 (R = 1/2) with a raw error rate of 0.15%. (C) DNA-40.5kb-EM-SE150 (R = 2/3) with a raw error rate of 0.20%. (D) DNA-40.5kb-MC-SE150 (R = 5/6) with a raw error rate of 0.14%.

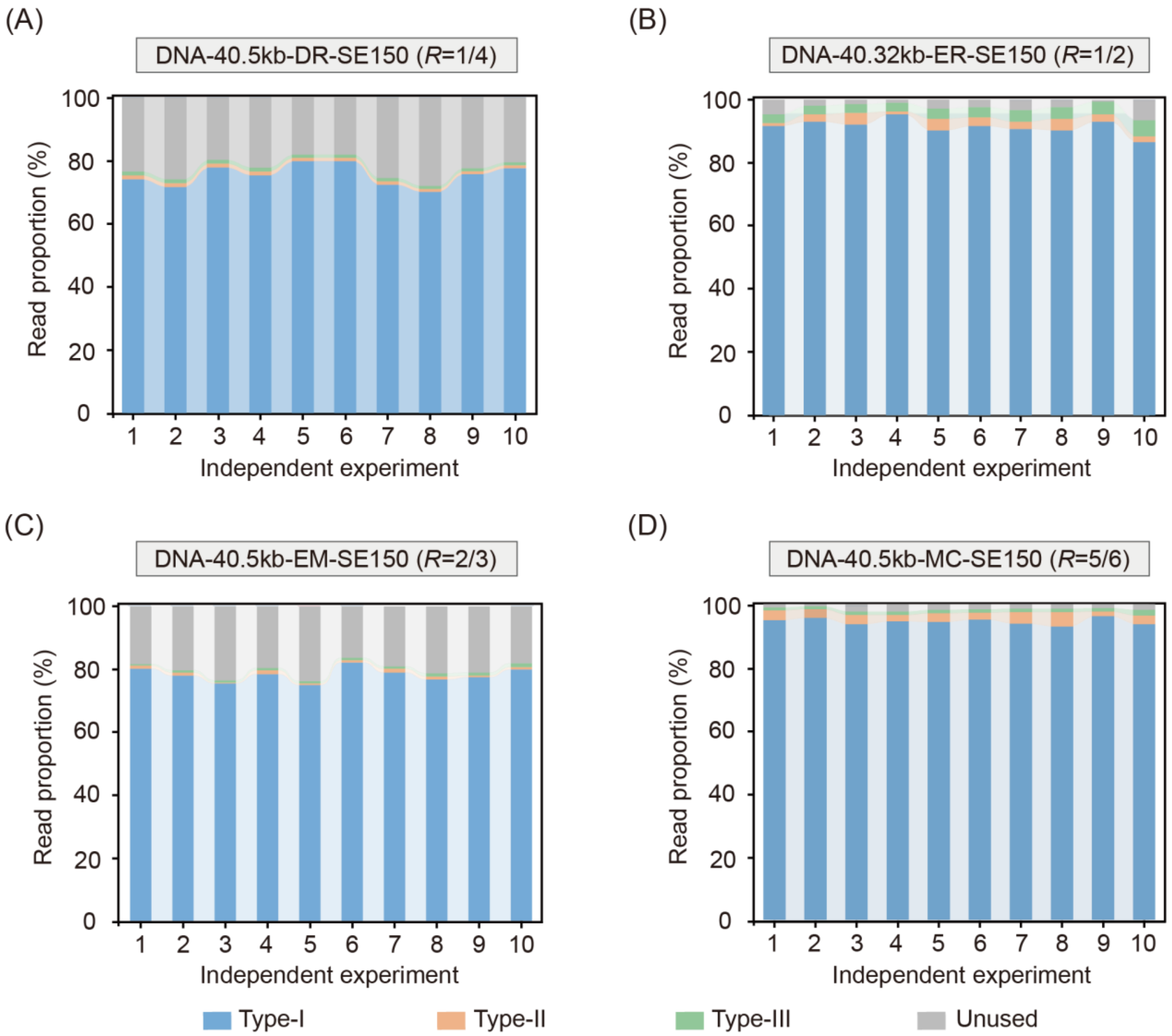

Figure S63 Proportions of different read types used in data recovery (real Illumina data). Stacked bar plots show the composition of utilized and unused reads across 10 independent experiments under two different raw error rates. Color codes indicate read categories: blue for Type-I, orange for Type-II, green for Type-III, and gray for unused reads. (A) DNA-40.5kb-DR-SE150 (R = 1/4) with a raw error rate of 0.18%. (B) DNA-40.32 kb-ER-SE150 (R = 1/2) with a raw error rate of 0.15%. (C) DNA-40.5kb-EM-SE150 (R = 2/3) with a raw error rate of 0.20%. (D) DNA-40.5kb-MC-SE150 (R = 5/6) with a raw error rate of 0.14%.

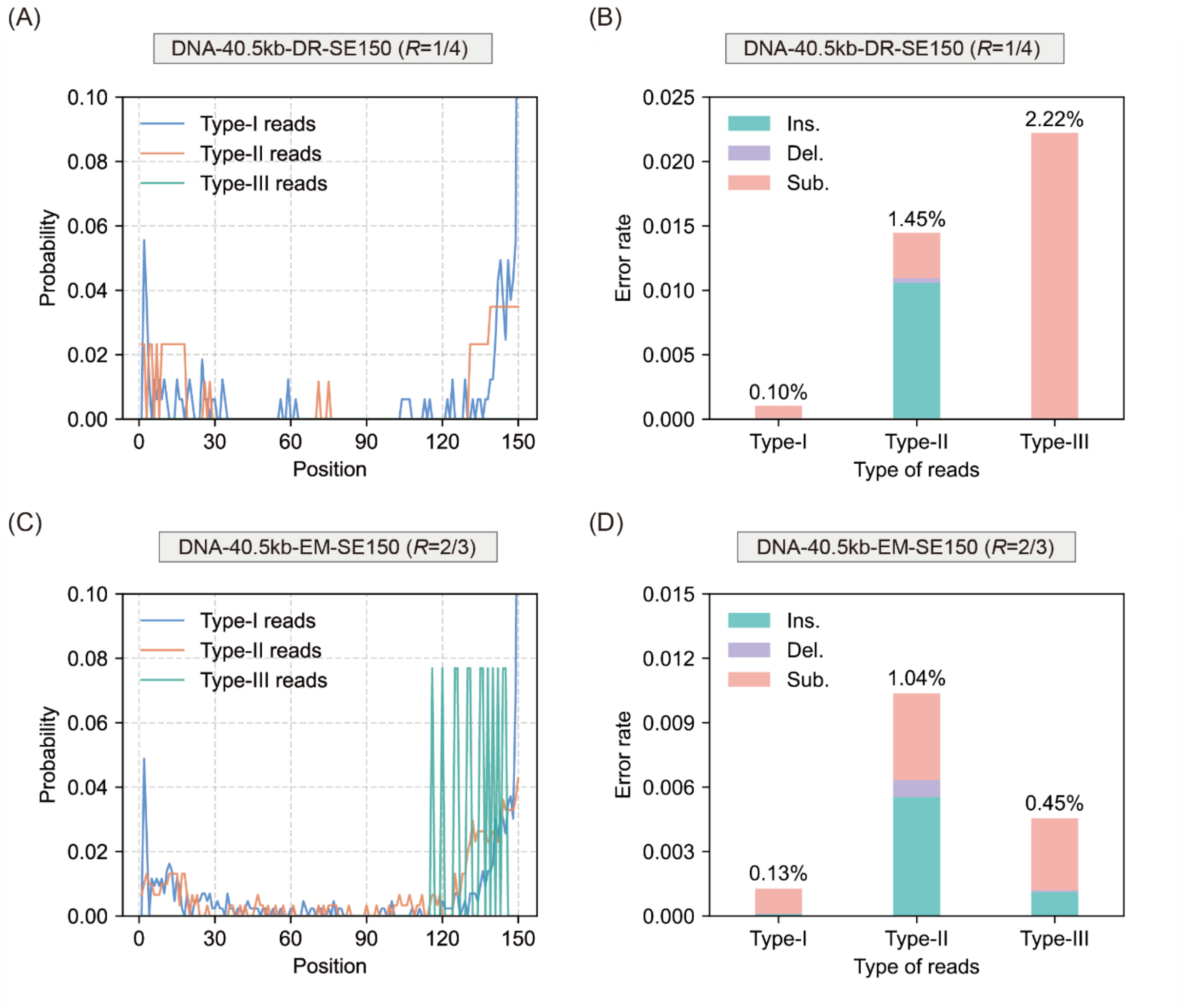

Figure S64 Distribution of indel error positions and error rates across types of reads (real Illumina data). (A) and (B) The results for DNA-40.5kb-DR-SE150 (R = 1/4) were obtained at 0.4× coverage using 183 independent experiments. (C) and (D) The results for DNA-40.5kb-EM-SE150 (R = 2/3) were obtained at 1.6× coverage using 83 independent experiments.

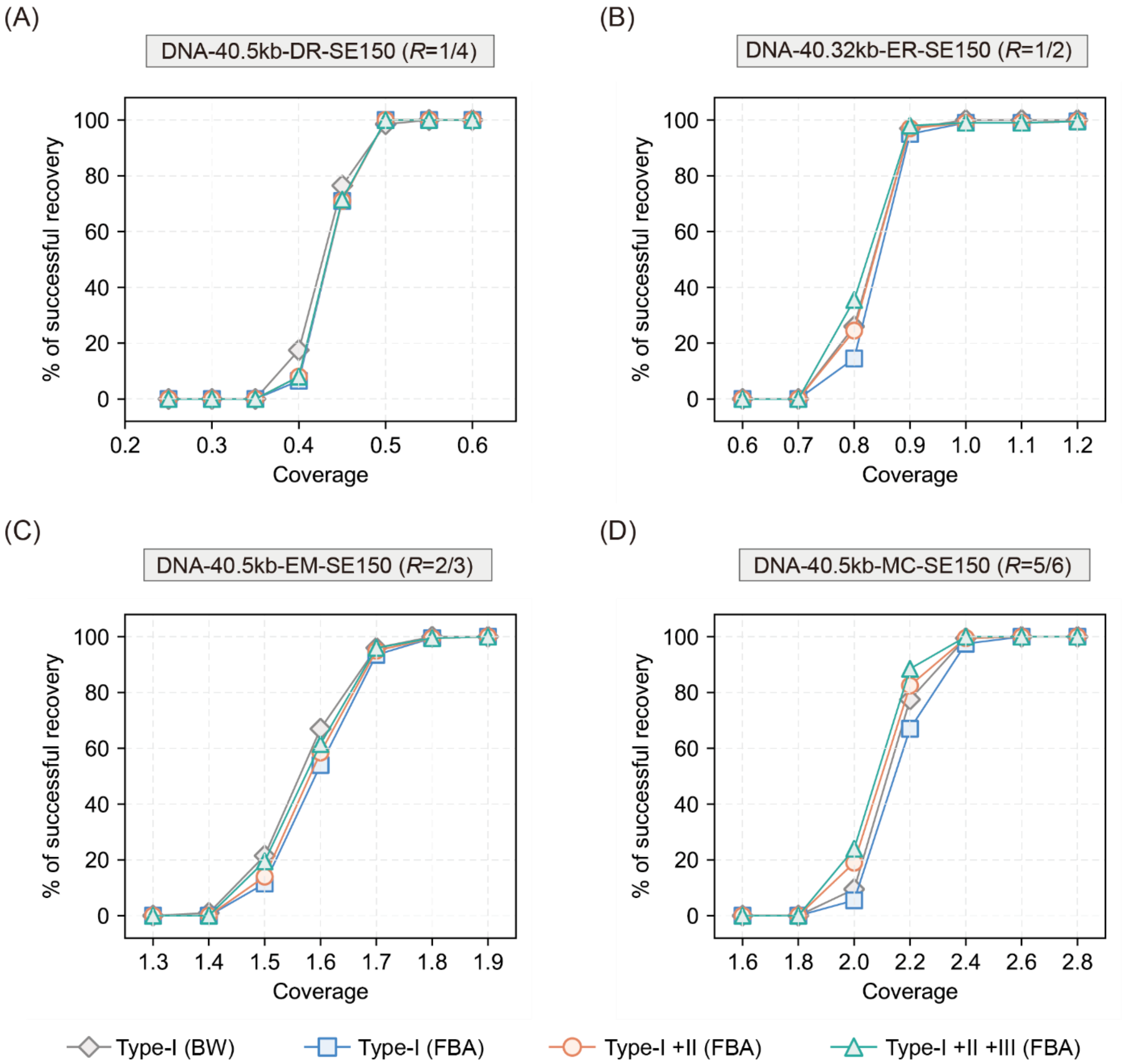

Figure S65 Data recovery using different types of reads (real Illumina data). Recovery performance was evaluated under varying code rates and raw error rates. All results are based on 200 independent retrieval trials. (A) DNA-40.5kb-DR-SE150 (R = 1/4) with a raw error rate of 0.18%. (B) DNA-40.32kb-ER-SE150 (R = 1/2) with a raw error rate of 0.15%. (C) DNA-40.5kb-EM-SE150 (R = 2/3) with a raw error rate of 0.20%. (D) DNA-40.5 kb-MC-SE150 (R = 5/6) with a raw error rate of 0.14%.

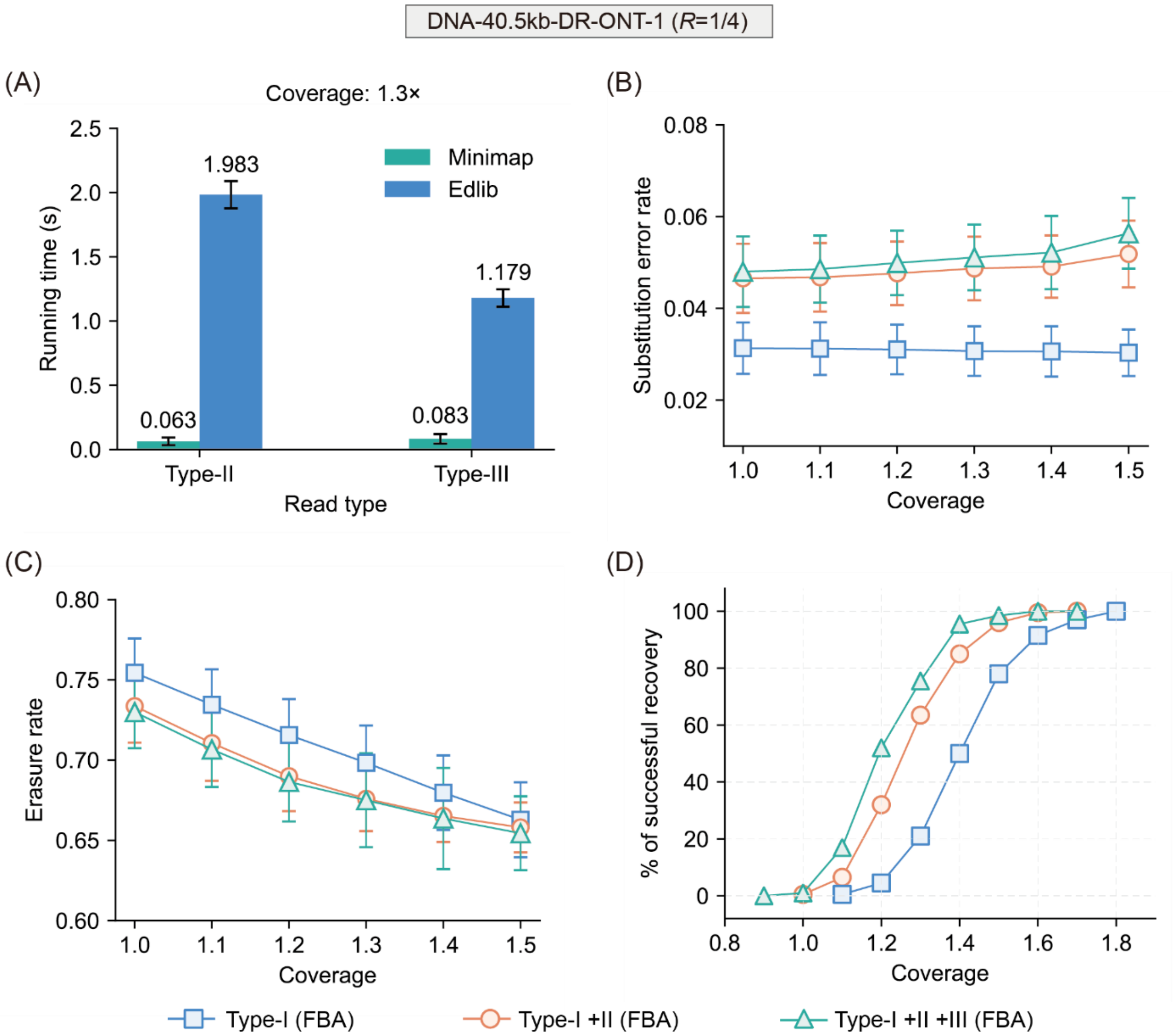

Figure S66 Evaluation of the accelerated readout scheme using Minimap2 on DNA-40.5kb-DR-ONT-1 (*R* = 1/4, raw error rate = 5.2%). (A) Running time of scaffold and regenerative reference alignment using Minimap2, evaluated at 1.3× sequencing coverage. (B) Substitution error rates before LDPC decoding for different types of reads. (C) Bit erasure rates before LDPC decoding for different types of reads. Error bars in (B) and (C) represent standard deviations calculated from 200 independent trials. (D) Recovery performance under varying sequencing coverages (200 trials).

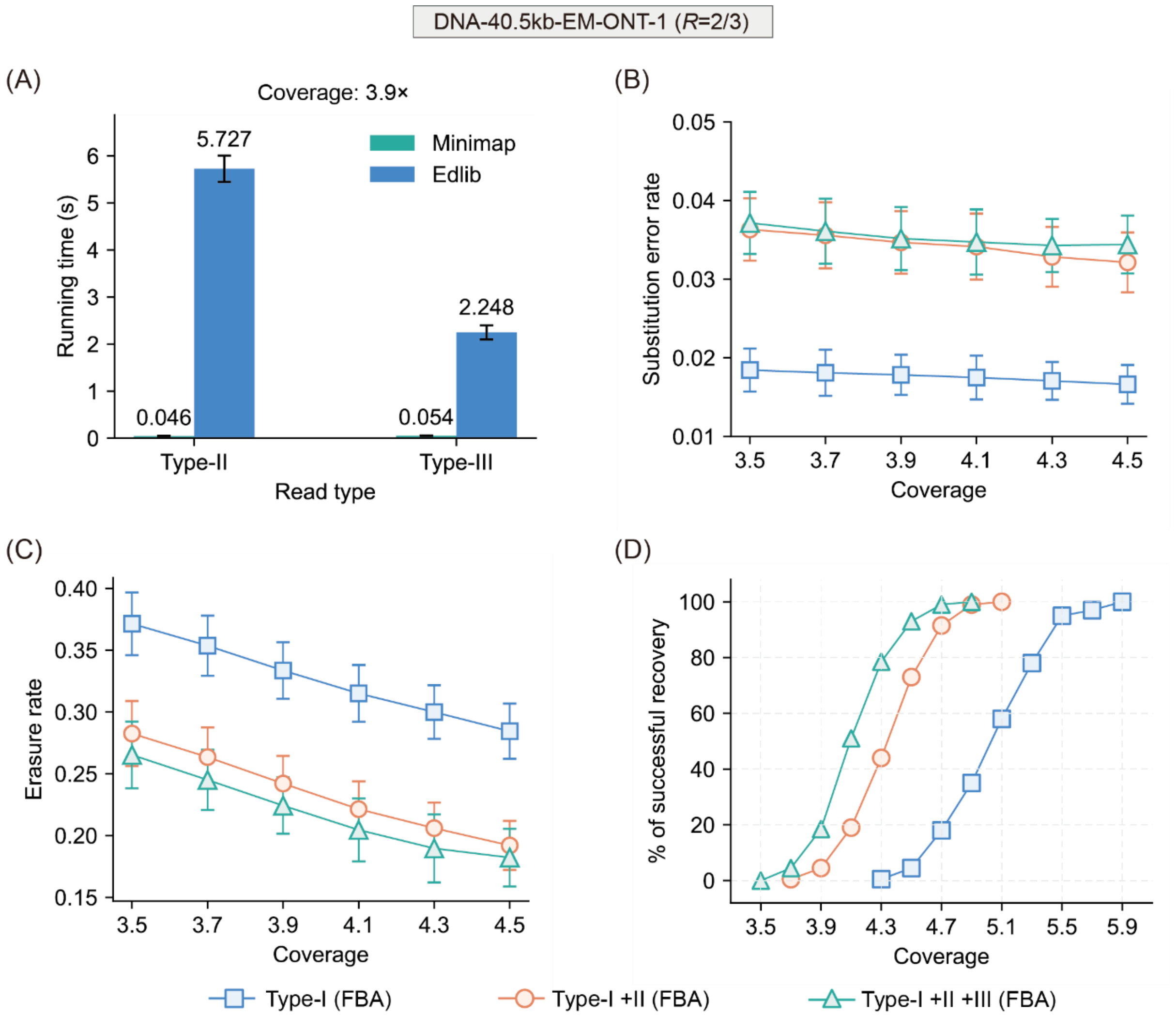

Figure S67 Evaluation of the accelerated readout scheme using Minimap2 on DNA-40.5kb-EM-ONT-1 (*R* = 2/3, raw error rate = 4.7%). (A) Running time of scaffold and regenerative reference alignment using Minimap2, evaluated at 3.9× sequencing coverage. (B) Substitution error rates before LDPC decoding for different types of reads. (C) Bit erasure rates before LDPC decoding for different types of reads. Error bars in (B) and (C) represent standard deviations calculated from 200 independent trials. (D) Recovery performance under varying sequencing coverages (200 trials).

Algorithm 1. BW-based fast readout

Input: Sequencing reads $R=\{r_1,r_2,\cdots\}$ and the known watermarks W_1 and W_2 of length $L(L>l)$.

Output: The recovered binary bitstream and image file.

for each sequence $r \in R$ of length l:

  r_rc =ReverseComplement(r)

  convert r to bit-pair sequences R_1 and R_2

  convert r_rc to bit-pair sequences Rrc_1 and Rrc_2

  for i from 0 to ($L-l$):

    calculate correlation value for r: $V_i\_\mathrm{fwd}=\sum_{j=0}^{l-1}(R_1[j]*W_1[i+j]+R_2[j]*W_2[i+j])$

    calculate correlation value for r_rc: $V_i\_\mathrm{rev}=\sum_{j=0}^{l-1}(Rrc_1[j]*W_1[i+j]+Rrc_2[j]*W_2[i+j])$

    $V_i=\max(V_i\_\mathrm{fwd},V_i\_\mathrm{rev})$

  end for

  calculate the correlation peak value $V=\max(V_0,V_1,\cdots V_{L-l})$ of each sequence

end for

for i from 0 to L:

  if $V \geq Thres$ of sequence $r \in R$:

    compute the bit-wise consensus across reads

    calculate the probabilities of bit “0” and bit “1” at each position i

  end if

end for

perform soft-decision decoding

return the recovered binary bitstream and image file.

Algorithm 2. FBA-based readout in a bootstrap manner

Input: Sequencing reads $R = \{r_1, r_2, \cdots\}$ and the known watermarks W_1 and W_2 of length $L(L > l)$.
Output: The recovered binary bitstream and image file.
for each sequence $r \in R$ of length l:
  calculate the correlation peak value $V = \max(V_0, V_1, \cdots V_{L-l})$
end for
for each sequence $r \in R$ meet $V \geq Thres$:
    classify r as Type-I read
    apply FBA to calculate the soft outputs S_{I}
end for
compute probability consensus of Type-I reads for decoding
if H·c^{T} ≠ 0
  compute the base-wise consensus across Type-I reads to generate scaffold reference
  for sequence $r \in R$ meet $V < Thres$:
    sequence r align to scaffold reference at base level
    if align success:
      classify r as Type-II read
      employ FBA calculate the soft information S_{II}
    else
      classify r as residual read
    end if
  end for
  compute probability consensus of Type-I and Type-II reads for decoding
  if H·c^{T} ≠ 0
    decoded codeword feedback to generate regenerative reference
    for residual read $r \in R$:
      sequence r align to regenerative reference at bit level
      if align success:
        classify r as Type-III read
        employ FBA calculate the soft information S_{III}
      end if
    end for
    compute probability consensus of Type-I, Type-II reads and Type-III reads for decoding
  end if
end if
return the recovered binary bitstream and image file.

Algorithm 3. Error rate computation using Edlib

Input: noisy read r with length of $L_{r,}$ and reference sequence R with length of L_R
Output: per-read error rate P_{err}

Perform pairwise alignment between r and R using Edlib
    result = edlibAlign(r, L_r, R, L_R, mode = local)

Initialize counters:
    $N_{ins} = 0$
    $N_{del} = 0$
    $N_{sub} = 0$

For each operation op in the results of alignment:
    if op == INSERTION:
        $N_{ins} = N_{ins} + 1$
    else if op == DELETION:
        $N_{del} = N_{del} + 1$
    else if op == MISMATCH:
        $N_{sub} = N_{sub} + 1$

L_{align} = result.alignmentLength
$P_{err} = (N_{ins} + N_{del} + N_{sub}) / L_{aligned}$
Return per-read error rate P_{err}
