# Supplementary material for: Fast bootstrap and reliable readout using hidden references for DNA data storage
Source: Imeta. 2026 Jan 11;5(1):e70105. doi: 10.1002/imt2.70105 (PMC13726579; doi:10.1002/imt2.70105)
Supplement: Supplementary file 2 [file imt270105-transc-0001-en.pdf]

Transcript for Video 1 – "Fast bootstrap and reliable readout using hidden references for DNA data storage"

This video demonstrates a fast bootstrap and reliable readout framework for DNA data storage based on multiple-fold hidden references. In our contribution, we employ a multi-stage alignment and error correction strategy, transforming the *de novo* readout into a resequencing-like workflow.

Readout of large DNA with shotgun sequencing faces two key challenges. On the one hand, unordered reads must be aligned based on overlapping regions. On the other hand, sequencing errors vary widely in types and error rates, and insertions and deletions are particularly difficult to correct. *De novo* assembly requires high sequencing coverage and substantial computational cost, and is further complicated by diverse sequencing errors.

The proposed scheme is inspired by resequencing pipelines in bioinformatics. Compared with de novo assembly, resequencing uses a reference genome to enable efficient genome analysis. Thus, we construct artificial DNA with hidden references for DNA data storage. Noisy reads are aligned to the hidden reference to enable assembly-free and reliable data retrieval.

The proposed scheme involves three steps: Step 1: Watermark encoding. The hidden watermark is embedded into the artificial DNA. Then, shotgun sequencing produces unordered, noisy reads. Step 2: Bootstrap filtering of sequencing reads. Multiple-fold hidden references are used to filter reads. The noisy reads fall into three types according to the different references. Step 3: Consensus & decoding. The probability consensus is generated for LDPC decoding and data recovery. Using every read through multiple-fold hidden references, the proposed scheme enables efficient and reliable decoding.

Highlight 1: Hidden watermark references facilitate rapid data recovery. Step 1: Sliding correlation to filter low-error reads. Step 2: Bit-wise consensus to generate soft information. Step 3: Soft-decision decoding to recover the original data.

Finally, we achieved error-free retrieval of original files at a coverage of 0.6–2.5×. It significantly outperforms traditional assembly-based methods.

Highlight 2: In the presence of indels, we employ the scaffold reference in combination with the forward-backward algorithm to rescue noisy reads. Step 1: Construct the scaffold references. Step 2: Filter Type-II reads. Type-II reads typically contain indels in the middle of the read. Step 3: Generate probability information. The forward-backward algorithm eliminates the effects of insertions and deletions and generates soft information. Step 4, Perform probability-level consensus and soft-decision decoding.

Read-by-read forward-backward algorithm, along with the incorporation of Type-II reads, progressively improved recovery performance, enabling error-free recovery at a coverage of 0.8–3.5×.

Highlight 3: We construct the regenerative reference to fill low-coverage regions. Step 1: Construct the regenerative reference from the decoded codeword. Step 2: Filter reads that help fill low-coverage regions. Step 3: Generate probability consensus, then apply soft-decision decoding.

Including Type-III reads significantly reduced erasures before decoding. Error-free recovery was achieved at a reduced coverage of 3.7×.

Our scheme progressively filters noisy reads, enabling fast bootstrap recovery.

The proposed bootstrap readout scheme can adapt to high error rate. At an raw error rate of ~5%, error-free recovery was achieved at a coverage of 4.3×.

The proposed scheme identifies reads with distinct features in a bootstrap manner. Compared to state-of-the-art methods, our approach achieved low-coverage readout across a wide error rate range.

In summary, we propose a multi-stage alignment and error correction strategy via multiple-fold hidden references, transforming the *de novo* readout into a resequencing-like workflow. Our solution well supports fast bootstrap and reliable readout for large DNA storage while tolerating insertions and deletions.
